# Synthesis and biological evaluation of novel ursolic acid analogues as potential α-glucosidase inhibitors

**DOI:** 10.1038/srep45578

**Published:** 2017-03-30

**Authors:** Pan-Pan Wu, Bing-Jie Zhang, Xi-Ping Cui, Yang Yang, Zheng-Yun Jiang, Zhi-Hong Zhou, Ying-Ying Zhong, Yu-Ying Mai, Zhong Ouyang, Hui-Sheng Chen, Jie Zheng, Su-Qing Zhao, Kun Zhang

**Affiliations:** 1 Department of Pharmaceutical Engineering, Faculty of Chemical Engineering and Light Industry, Guangdong University of Technology, Guangzhou, 510006, China; 2 Faculty of Chemical & Environmental Engineering, Wuyi University, Jiangmen, 529020, China; 3 International Healthcare Innovation Institute (Jiangmen), Jiangmen, 529020, China

## Abstract

Ursolic acid (UA) is a major pentacyclic triterpenoid in plants, vegetables and fruits, which has been reported to have a potential anti-diabetic activity. Despite various semi-synthetic ursolic acid derivatives already described, new derivatives still need to be designed and synthesized to further improve the anti-diabetic activity. In the present study, two series of novel UA derivatives, were synthesized and their structures were confirmed. The enzyme inhibition activities of semi-synthesized analogues against α-glucosidase were screened *in vitro*. The results indicated that most of UA derivatives showed a significant inhibitory activity, especially analogues UA-O-i with the IC_50_ values of 0.71 ± 0.27 μM, which was more potential than other analogues and the positive control. Furthermore, molecular docking studies were also investigated to verify the *in vitro* study. Structure modification at the C-3 and C-2 positions of UA was an effective approach to obtain the desired ligand from UA, whose structure was in accordance with the active pocket. Besides, suitable hydrophobic group at the position of C-2 might play an important role for the docking selectivity and binding affinity between the ligand and the homology modelling protein. These results could be helpful for designing more potential α-glucosidase inhibitors from UA in the future.

Diabetes mellitus (DM) is a chronic metabolic disorder, which is characterized by hyperglycemia^1^,^2^,^3^. It occurs either when the pancreas does not produce enough insulin or when the body cannot effectively use the insulin it produces^1^,^4^. According to the statistics, it was estimated that diabetes and high blood glucose were the main causes of about 4 million deaths in 2012. Moreover, more than 8.5% adults in the world population aged from 18 and more had diabetes in 2014, with 90% having type 2 diabetes mellitus (T2DM), comprising the majority of people with diabetes around the world^4^. World Health Organization (WHO) projected that DM will be the 7th leading cause of death in 2030^5^. More and more studies indicated that T2DM is normally associated with overly rich nutrition, as well as with the development of several comorbidities, including renal, cardiac, and hepatic disorders^6^. Some approaches are available for management of T2DM, which include hyperglycemia treatment, diabetic comorbidities prevention and metabolism adjustment^7^,^8^. However, currently available approaches have some drawbacks, such as safety concerns, limited efficacy, failure in metabolism adjustment, and the prevention of diabetic complication^9^. Therefore, DM has become a serious problem for people worldwide^10^, which necessitates the development of new therapeutic agents for T2DM, as well as in the prevention and/or treatment of diabetes and its related comorbidities.

Triterpenoids are a large and structurally diverse group of natural products that display nearly 200 distinct skeletons^11^,^12^. Ursolic acid (UA, (*1S,2R,4aS,6aS,6bR,10S,12aR) -10-hydroxy-1,2,6a,6b,9,9,12a-heptamethyl-1,2,3,4,4a,5,6,6a,6b,7,8,8a,9,10,11,12,12a,12b,13,14b-icosahydropicene-4a-carboxylic acid*, 1) is abundantly present in some medicinal herbs and fruits (Fig. 1)^13^. It possesses a large number of bioactivities, including antimicrobial^14^,^15^, antiviral^16^, antitumor^17^,^18^,^19^, anti-inflammatory^20^,^21^, antioxidative^22^ activities and so on. And it has become a well-known natural pentacyclic triterpenoid carboxylic acid. Over the past few decades, UA had been treated as an effective molecular template in developing more leading potential compounds with diverse pharmacological activities^23^,^24^,^25^. However, the potential applications of UA and its analogues for developing novel antidiabetic agent for treatment of DM and its complications are far from being comprehensively exploited.

Based on our previous studies, UA and some of its analogues have significant inhibitory activity to α-glucosidase, which might play an important role for treatment of DM and its complications^26^,^27^. In this study, two series of UA analogues have been described and synthesized. In order to explore the structure-activity relationships of the introduced acyl substituent at C-3 group, a series of new UA ester analogues were obtained by esterification with appropriate acid chlorides, in which *N, N*-dimethyl-4-aminopyridine (DMAP) was applied as a catalyst. What’s more, another series of some new triterpenic analogues were also prepared from UA involved in two steps, Jones oxidation and Claisen Schmidt condensation at the position of C-3 and C-2 of UA respectively. And the bioactivities of these new analogues against α-glucosidase were screened *in vitro*. Furthermore, molecular docking studies were also performed with an aim to develop more potential α-glucosidase inhibitory agents. This is the first study associated with the anti-diabetic properties of these new UA analogues, in which α-glucosidase (PDB: 1UOK) was chosen as the docking target.

## Results and Discussion

### Chemistry

**Figure 2:**
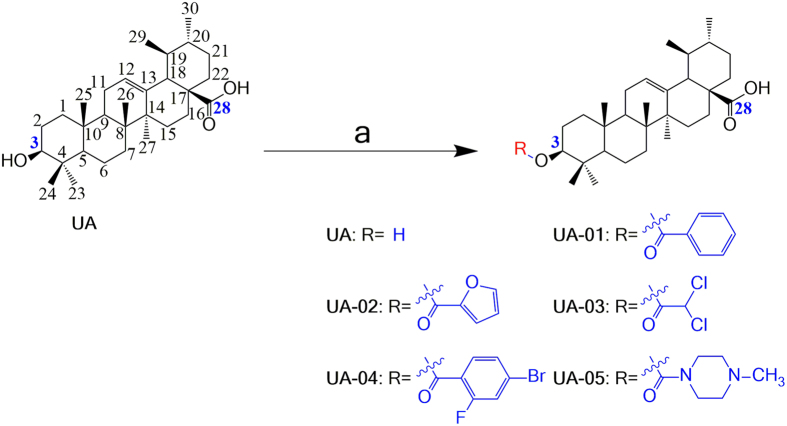
Synthesis of analogues UA-01~UA-05 from UA. Reagents and conditions: (**a**) anhydride or acid chloride, DMAP, pyridine, reflux, overnight.

UA analogue compounds UA-01~UA-05 were synthesized according to Fig. 2. The benzoic anhydride (1.2eq.) or appropriate acid chloride (1.2 eq.) was added to the stirred mixture of UA (200 mg, 0.44 mmol) and DMAP (1.0 eq.) in anhydrous pyridine, and the mixture was refluxed overnight and concentrated in vacuum under reduced pressure. Then the result mixture was washed with distilled water to remove salt and extracted with diethyl ether (30 × 3). The organic layer was dried with magnesium sulfate. And the solvent of diethyl ether was removed under reduced pressure to get the target ester UA-01~UA-05, which was purified over a chromatograph column of silica gel by using ethyl acetate-petroleum ether (1: 10, V/V) as the eluent to obtain the final compounds (Fig. 2). Their structures were characterized by the application of ^1^H NMR, ^13^C NMR, melting point (mp) and electrospray ionization mass spectrometry (ESI-MS).

**Figure 3:**
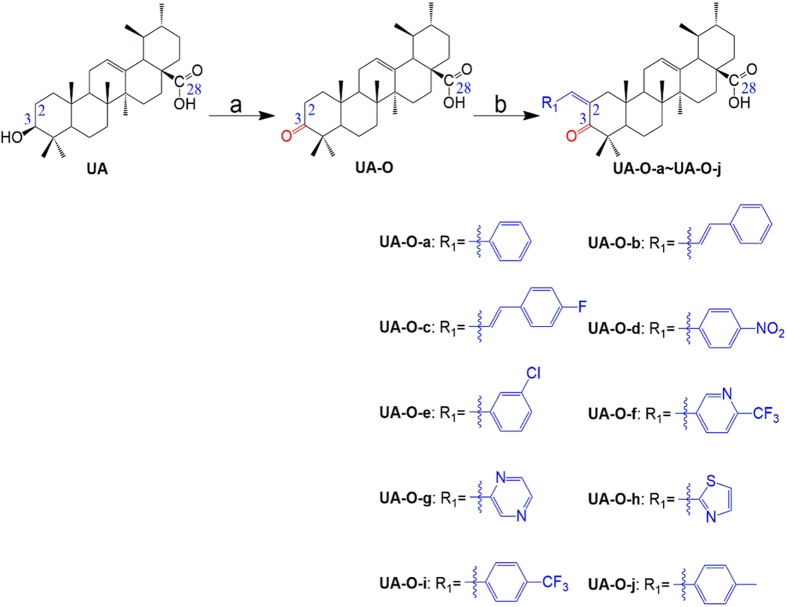
Synthesis of analogues UA-O-a~UA-O-j from UA. Reagent and condition: (**a**) CrO_3_, H_2_SO_4_, acetone, 0 °C, 1 h; (**b**) R_1_-CHO, KOH, ethanol, r.t., overnight.

UA analogue compounds UA-O-a~UA-O-j were synthesized according to Fig. 3. UA (200 mg, 0.44 mmol) was dissolved in 20 mL acetone at 0 °C; Jones reagent was added to the reaction dropwise until the colour of the solution was stable to be slight brown, indicating that the Jones reagent was sufficient to oxidize the group of hydroxyl at the C-3 position to form active intermediate UA-O. UA-O-a~UA-O-j were prepared by Claisen Schmidt condensation of UA-O with various aldehydes in the presence of ethanolic potassium hydroxide at room temperature in good yield (Fig. 3). Their structures were characterized by the application of ^1^H NMR, ^13^C NMR, melting point (mp) and electrospray ionization mass spectrometry (ESI-MS).

### *In vitro* α-glucosidase inhibition assay of the UA analogues

In this assay, α-glucosidase from baker’s yeast has been selected and used in this model according to the procedure described in the previous study with a slight modification^28^,^29^,^30^. The activity of α-glucosidase was determined by monitoring *p*-nitrophenol (PNP) released from *p*-nitrophenol-α-D-glycopyranoside (PNPG) at 405 nm. All examined samples of UA analogues were dissolved in DMSO to generate the stock solution, with the concentrations ranging from 5 μM to 4000 μM. During the enzyme inhibitory assay, each sample’s stock solution was diluted with 0.1 M phosphate buffer solution (pH = 6.8), and the final concentration of enzyme solution was 0.1 U/mL. Then 10 min pre-incubation at 37 °C was conducted. After that, the substrate (PNPG, 1 mmol/L) was added to initiate the reaction. The reaction was incubated for 30 min at 37 °C before it was terminated by adding 1 mol/L Na_2_CO_3_ solution, and the optical density value was measured at 405 nm by using a Multimodel Plate Reader (Infinite 200). Each assay was performed three times.

**Figure 4:**
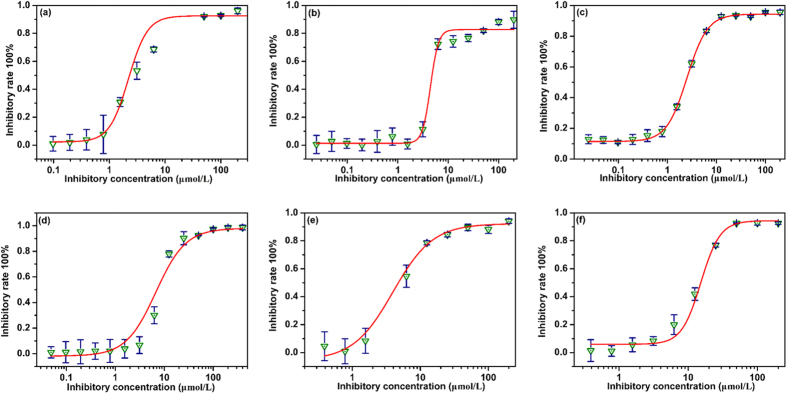
Inhibitory activities of UA analogues UA-01~UA-05 against α-glucosidase from baker’s yeast. The data reported representing the mean (n = 3) ± SD.

**Figure 5:**
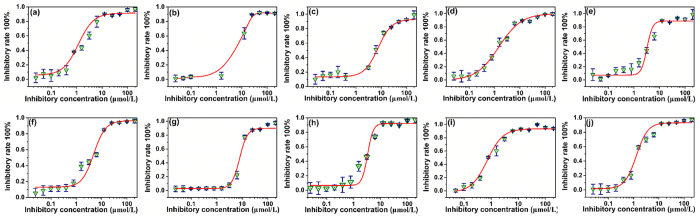
Inhibitory activities of UA analogues UA-O-a~UA-O-j against α-glucosidase from baker’s yeast. The data reported representing the mean (n = 3) ± SD.

The enzyme activity was measured at a fixed substrate concentration, in which a series of sample concentrations were introduced. The IC_50_ value was expressed as percentage inhibitions relative to a control assay with no inhibitor added. Acarbose was used as positive control in this assay for comparison. The IC_50_ values and inhibition curves were presented in Figs 4 and 5 and Tables 1 and 2. As shown in Fig. 4 and Table 1, The IC_50_ values of this series of UA ester analogues (UA-01~UA-05) ranged from 2.51 ± 0.02 μM to 15.23 ± 0.47 μM, in which UA-02 (IC_50_ = 2.51 ± 0.02 μM) and UA-04 (IC_50_ = 3.94 ± 0.10 μM) were better than the parent compound UA (IC_50_ = 5.08 ± 0.70 μM) and the positive control. And the inhibitory effect of analogue UA-01 (IC_50_ = 4.98 ± 0.21 μM) presented similarly with UA. However, the inhibition activity of analogue UA-03 (IC_50_ = 6.72 ± 0.33 μM) and UA-05 (IC_50_ = 15.23 ± 0.47 μM) were less than that of UA. What’s more, the results were in accordance with our previous study as well^26^, indicating that side chain and large ester group at the C-3 position might decrease the enzyme inhibition activity.

Each experiment was performed in quadruplicate. The data presented representing the mean (n = 4) ± SD.

^*a*^IC_50_ value representing the concentration that caused a 50% loss of activity.

^*b*^Acarbose, positive control.

As showed in Fig. 5 and Table 2, different kinds of aldehyde possessing less than one α-H could be condensed with the intermediate compound (UA-O), which has two α-H at the position of C-2, to achieve this series of UA analogues from UA-O-a to UA-O-j. And different condensation groups have different contributions to their enzyme inhibition activities. The IC_50_ of this series of analogues ranges from 0.71 ± 0.04 to 10.32 ± 0.55 μM. According to the enzyme inhibition results, it could be summarized that the bioactivities of analogues UA-O-b (IC_50_ = 10.32 ± 0.55 μM) and UA-O-c (IC_50_ = 7.49 ± 0.29 μM) are lower than that of other types of aldehyde with no α-H. Besides, analogue UA-O-g (IC_50_ = 7.87 ± 0.34 μM) with the pyrazine group at C-2 position of UA-O might decrease the α-glucosidase inhibition activity. The inhibition activities of other analogues are better than that of UA (IC_50_ = 5.08 ± 0.70 μM) and the positive control, especially the analogue UA-O-i (IC_50_ = 0.71 ± 0.04 μM) presenting the best inhibition activity than the others. What’s more, the result also implied that a proper group at the position of C-2 could increase its bioactivity of against α-glucosodase.

Each experiment was performed in quadruplicate. The data presented representing the mean (n = 4) ± SD.

^*a*^IC_50_ value representing the concentration that caused a 50% loss of activity.

^*b*^Acarbose, positive control.

### Structure activity relationship

In the study, a total of sixteen analogues of UA (UA-01~UA-05, UA-O and UA-O-a~UA-O-j) were obtained and their structure activity relationships (SAR) against α-glucosidase were deduced. The enzyme inhibition activities were decreased in varying degrees while the hydroxyl group of UA was esterified with different kinds of acyl chlorides, especially the big size of the ester group, which was according with the results of molecular docking study and our previous SAR study. This might be on account of the size of the active site of the target protein. Moreover, the α-glucosidase inhibition activity was more than doubled while the hydroxyl group of UA was oxidized into ketone (UA-O, IC_50_ = 2.47 ± 0.14 μM)^27^. And analogues from UA-O-a to UA-O-j were obtained based on this ketone. Their effects of enzyme inhibition were changed a lot along with the substituent group of aldehyde. Analogues UA-O-b (IC_50_ = 10.32 ± 0.55 μM) and UA-O-c (IC_50_ = 7.49 ± 0.29 μM) have higher IC_50_ value than UA-O, implying that the length of the substituent group might have a significant effect on its activity. The longer the substituent group, the less the inhibitory activity. Meanwhile, when the substituent groups at the C-2 position were changed into phenyl group or substituent phenyl groups, the inhibitory activity against α-glucosidase could be increased dramatically. Among them, the analogue UA-O-i (IC_50_ = 0.71 ± 0.04 μM) could be obtained with the substituent group of *p*-trifluoromethyl benzaldehyde, showing a potential inhibitory activity against α-glucosidase. This result might be attributed to both the selectivity and affinity of the target site with the target compound.

### Molecular docking mode

In order to verify and predict the enzyme inhibition potency of these UA derivatives in our study, SYBYL 2.0, a molecular docking software was applied to demonstrate the relationship between the theory and practice. Molecular docking study can not only expound how these UA derivatives conjugate with the target protein, but also can be treated as a guidance for the design of enzyme inhibitors. So the docking studies were performed according to our previous study, and the binding models of UA derivatives with the binding pocket of α-glucosidase were investigated to give insight into the inhibition mechanism and to understand their structure-relationship activity.

In this molecular docking model, the reasonable binding mode was identified by applying the homology modelled structure of α-glucosidase, in which the structure of oligo-1, 6-glucosidase from *Saccharomyces cerevisiae* (PDB: 1UOK) was chosen as the target protein for this docking model. And the sequence similarity is around 62.0% and the sequence identity is around 38.0%, as compared with α-glucosidase^31^.

**Figure 6:**
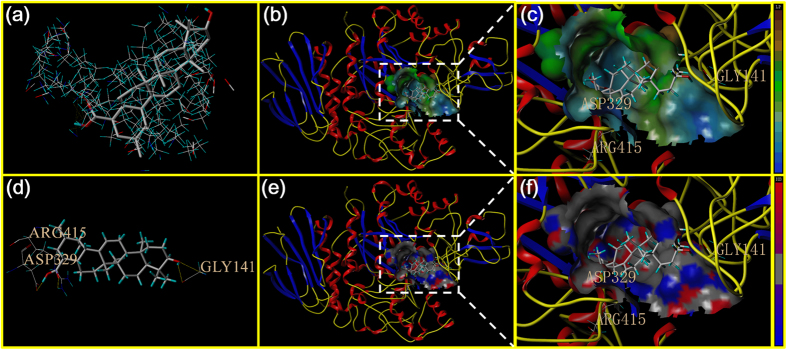
Homology model of the yeast α-glucosidase with analogue UA. (**a**) The binding mode of UA docked with the prototype molecular of the active site. (**b**) and (**c**) Active site MOLCAD surface representation of lipophilic potential. (**d**) The active site was surrounded and interacted with the amino acid. (**e**) and (**f**) Active site MOLCAD surface representation of hydrogen bonding.

As is indicated in Fig. 6(a), the parent compound UA could be inserted into the target protein protomol. As shown in Fig. 6(d), UA could be interacted with three amino residues, including ARG415, ASP329 and GLY141. As is depicted in Fig. 6(b,c), the lipophilic potential interaction between UA and the catalytic pocket was studied. The hydroxyl group at C-3 position of UA was closed to the hydrophobic region of the active pocket. Similarly, hydrogen bonding interaction between UA and surface of the catalytic pocket was also presented in Fig. 6(e,f), in which the hydrogen bonds could be formed to increase the affinity between the target site and UA. And the binding free energy of this docking model was −3.007 kcal/mol. However, a small lipophilic potential channel existed inside of the active pocket, and the parent compound could not be inserted inside to form hydrogen bond or other interactions.

**Figure 7:**
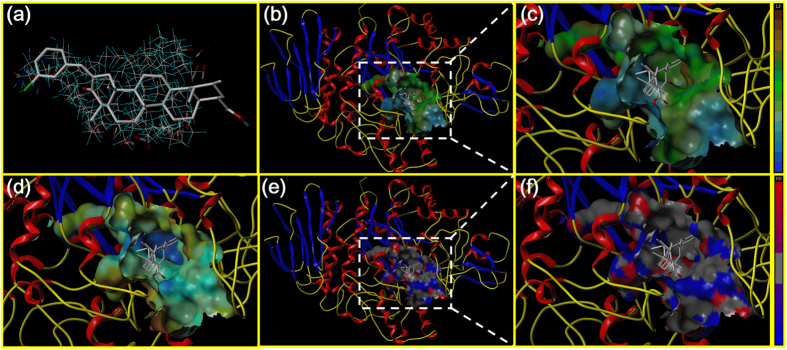
Homology model of the yeast α-glucosidase with analogue UA-O-e. (**a**) The binding mode of UA-O-e docked with the prototype molecular of the active site. (**b**) and (**c**) Active site MOLCAD surface representation of lipophilic potential. (**d**) Active site MOLCAD surface representation of electrostatic potential. (**e**) and (**f**) Active site MOLCAD surface representation of hydrogen bonding.

All of the synthesized UA derivatives were docked with the developed homology model of α-glucosidase (PDB: 1UOK). In the study, one potential analogue (UA-O-e) against α-glucosidase was presented in Fig. 7. The binding free energy of analogue UA-O-e was calculated as −4.084 kcal/mol, which was much lower than that of UA and other analogues. As shown in Fig. 7(a), the structure of UA-O-e was in accordance with the structure of the active site. Besides, the substituent group could be inserted and fit into the channel, which might be the most important contribution to the lower binding free energy. The lipophilic potential interaction between UA-O-e and the catalytic pocket was presented in Fig. 7(b,c), in which the ligand of UA-O-e could be completely inserted into the site pocket. It could be concluded that the lipophilic pocket interacted with the hydrophobic portion of UA-O-e was the major contribution to the docking study. Besides, the electrostatic potential interaction was presented in Fig. 7(d). And the hydrogen bonding of active site MOLCAD surface interacted with the ligand was also performed, shown in Fig. 7(e) and (f). However, there is no amino residue that could be interacted with the ligand to form the hydrogen bond for the increase of the docking affinity. Therefore, the ligand possessing a suitable structure together with some essential groups may be contributed to its α-glucosidase inhibition potential.

**Figure 8:**
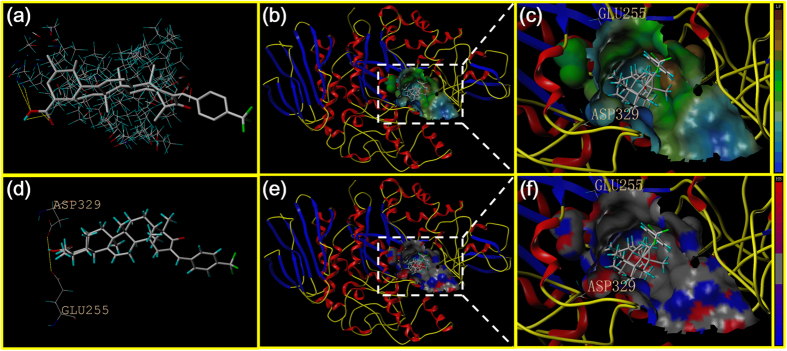
Homology model of the yeast α-glucosidase with analogue UA-O-i. (**a**) The binding mode of analogue UA-O-i docked with the prototype molecular of the active site. (**b**) and (**c**) Active site MOLCAD surface representation of lipophilic potential. (**d**) The active site was surrounded and interacted with the amino acid. (**e**) and (**f**) Active site MOLCAD surface representation of hydrogen bonding.

The docking study of analogue UA-O-i was depicted in Fig. 8. As shown in Fig. 8(a,d), the hydrophilic portion could be inserted into the catalytic pocket, which was mainly surrounded by two amino residues, that is residue GLU255 and residue ASP329. As illustrated in Fig. 8(b,c), the study of MOLCAD lipophilic potential suggested that the free carboxyl group of analogue UA-O-i was closed to the hydrophobic region of the active site. This might be attributed to the structure of *p*-trifluoromethyl benzene group and the selectivity of the active site. Furthermore, the MOLCAD hydrogen bonding study of the binding surface revealed that two hydrogen bond donors were presented in the hydrophobic pocket while analogue UA-O-i served as an acceptor by forming two hydrogen bonds, showed in Fig. 8(e) and (f).

**Figure 9:**
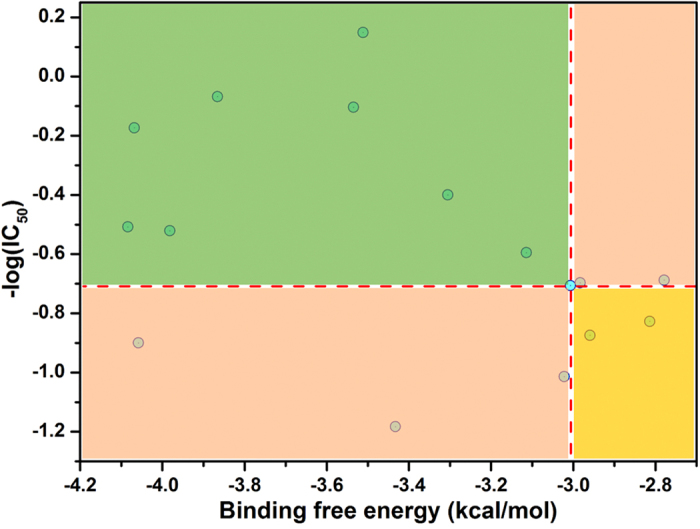
Correlation of binding free energies with inhibitory activities for UA and its analogues.

In order to gain an insight into the relationship between the modelling study and the *in vitro* α-glucosidase inhibition activity, the predicted binding free energies of all ligands docking with the target protein of α-glucosidase were calculated and presented in Tables 1 and 2. The correlation of the predicted binding free energy and the enzyme inhibition activity was illustrated in Fig. 9. According to the activity of UA, the correlation was divided into four parts. Most of the examined analogues were presented in the green area, in which the enzyme inhibition *in vitro* and the binding free energy were lower than that of UA. Furthermore, the result also indicated that the practise inhibition activity was in accordance with the theoretical results. It also suggested that the homology protein of α-glucosidase could be applied in the docking model to provide some guidance for the development of α-glucosidase inhibitor.

## Conclusion

In summary, two series of UA derivatives were synthesized at the positions of C-2 and C-3, and their enzyme inhibitory activity assays against α-glucosidase *in vitro* have been studied. Most of the UA derivatives showed better potency than UA and the positive control, acarbose. However, the bigger-sized substituent group at the position of C-3 showed a negative effect for their bioactivity. Besides, the heterocyclic substituent group condensed at the C-2 position also decreased their α-glucosidase inhibitory activity. As to analogue UA-O-i, which could be obtained by coupling UA-O with *p*-trifluoromethyl benzaldehyde, presented a great improvement of enzyme inhibition activity (IC_50_ = 0.71 ± 0.04 μM). These results could also be confirmed by the molecular docking study. The structures of the ligand and the docking pocket were the key factors for the docking binding model and affinity, which played an important role for their potential activity. In conclusion, UA new derivative condensed with a suitable aldehyde at the position of C-2 with a stable docking model could be a promising agent as potential α-glucosidase inhibitor.

## Materials and Methods

### General Remarks

UA was supplied by Nanjing Zelang Medical Technology Co., Ltd., with a purity of over 98%. Silica gel (100–200 or 200–300 mesh) used in column chromatography was bought from Adamas Reagent Ltd. (Shanghai China) or Tsingtao Marine Chemistry Co., Ltd. Other reagents and solvents were purchased from Adamas Reagent Ltd. (Shanghai China) or other commercial suppliers in their analytically or chemically pure forms and used without purification. TLC was performed on pre-coated silica gel F_254_ plates (0.25 mm; E. Merck); the starting material and product were detected by either viewed under UV light or treated with an ethanolic solution of *p*-anisaldehyde spray followed by heating.

^1^H NMR and ^13^C NMR spectra were recorded on a Bruker AVANCE 400 NMR spectrometers under a standard condition; chemical shifts were measured in ppm downfield from TMS as internal standard. Melting point were tested by microscopic melting point apparatus of X-4 from Beijing Tech Instrument Co., LTD. Mass spectra were determined on an apparatus of LC-MS-2010A and the results were presented as m/z. The enzyme inhibition activity was measured by using a Multimodel Plate Reader (Infinite 200).

### General procedure for the preparation of UA analogues (UA-01~UA-05)

According to our previous study^26^, analogues UA-01~UA-05 in Fig. 2 could be prepared after UA was esterified with different anhydrides or chloride acids. Analogues UA-01~UA-05 were purified on silica gel column, in which petroleum ether/ethyl acetate was chosen as the eluent. Five UA analogues were synthesized and characterized.

(*1S,2R,4R,6aS,6bR,10S,12aR*)-*10*-(*benzoyloxy*)-*1,2,6a,6b,9,9,12a-heptamethyl*-*1,2,3,4,4a,5,6,6a,6b,7,8,8a,9,10,11,12,12a,12b,13,14b*-*icosahydropicene-4-carboxylic acid* (**UA-01**, C_37_H_52_O_4_). According to the general procedure, UA was treated with benzoic anhydride at room temperature overnight, and then purified on a silica gel column with petroleum ether/ethyl acetate (v/v 10:1) as the eluent to obtain compound UA-01(R_f_ = 0.57). Yield: 60%; white powder; mp: 266–268 °C; ^1^H NMR (400 MHz, CDCl_3_) δ 7.98 (d, J = 7.6 Hz, 2 H), 7.48 (t, J = 7.3 Hz, 1 H), 7.37 (t, J = 7.6 Hz, 2 H), 7.19 (s, 1 H), 5.19 (s, 1 H), 4.68 (dd, J = 10.7, 5.3 Hz, 1 H), 2.13 (d, J = 11.3 Hz, 1 H), 2.02–1.76 (m, 4 H), 1.76–1.55 (m, 6 H), 1.54–1.40 (m, 4 H), 1.39–1.15 (m, 7 H), 1.03 (s, 3 H), 0.95 (s, 6 H), 0.89 (d, J = 5.0 Hz, 6 H), 0.85–0.77 (m, 4 H), 0.73 (s, 3 H).^13^C NMR (100 MHz, CDCl_3_) δ 182.8, 166.5, 138.2, 132.9, 131.2, 129.7, 128.47, 126.0, 81.8, 55.6, 52.8, 48.1, 47.7, 42.2, 39.7, 39.2, 39.0, 38.5, 38.3, 37.1, 36.9, 33.1, 30.8, 29.9, 28.4, 28.2, 24.3, 23.8, 23.5, 21.3, 18.4, 17.3, 17.2, 17.2, 15.7. ESI-MS m/z 559.4 [M-H]^−^.

(*1S,2R,4R,6aS,6bR,10S,12aR*)-*10*-((*furan-2-carbonyl) oxy*)-*1,2,6a,6b,9,9,12a-heptamethyl-1,2,3,4,4a,5,6,6a,6b,7,8,8a,9,10,11,12,12a,12b,13,14b-icosahydropicene-4-carboxylic acid* (**UA-02**, C_35_H_50_O_5_). According to the general procedure, UA was treated with 2-Furoyl chloride at room temperature overnight, and then purified on a silica gel column with petroleum ether/ethyl acetate (v/v 10:1) as the eluent to obtain compound UA-02 (R_f_ = 0.55). Yield: 54%; white powder; mp: 227–228 °C; ^1^H NMR (400 MHz, CDCl_3_) δ 7.57 (s, 1 H), 7.13 (d, J = 2.9 Hz, 1 H), 6.49 (d, J = 1.0 Hz, 1 H), 5.23 (s, 1 H), 4.72 (t, 1 H), 2.18 (d, J = 11.1 Hz, 1 H), 2.06–1.81 (m, 4 H), 1.80–1.61 (m, 6 H), 1.59–1.45 (m, 4 H), 1.43–1.24 (m, 7 H), 1.08 (s, 4 H), 0.99 (s, 3 H), 0.94 (d, J = 12.9 Hz, 9 H), 0.86 (d, J = 6.3 Hz, 4 H), 0.77 (s, 3 H). ^13^C NMR (100 MHz, CDCl_3_) δ 184.4, 158.8, 146.2, 145.4, 138.1, 125.8, 117.5, 111.8, 81.8, 55.5, 52.6, 48.1, 47.6, 42.0, 39.7, 39.1, 39.0, 38.4, 38.2, 37.1, 33.0, 30.7, 29.8, 28.3, 28.1, 24.2, 23.8, 23.7, 23.4, 21.3, 18.3, 17.2, 17.1, 16.9, 15.7. ESI-MS m/z 549.3 [M-H]^−^.

(*1S,2R,4R,6aS,6bR,10S,12aR*)-*10*-(*2,2-dichloroacetoxy*)-*1,2,6a,6b,9,9,12a-heptamethyl-1,2,3,4,4a,5,6,6a,6b,7,8,8a,9,10,11,12,12a,12b,13,14b-icosahydropicene-4-carboxylic acid* (**UA-03**, C_32_H_48_Cl_2_O_4_). According to the general procedure, UA was treated with 2,2-Dichloroacetyl chloride at room temperature overnight, and then purified on a silica gel column with petroleum ether/ethyl acetate (v/v 10:1) as the eluent to obtain compound UA-03 (R_f_ = 0.45). Yield: 66%; white powder; mp: 235–237 °C; ^1^H NMR (400 MHz, CDCl_3_) δ 5.94 (s, 1 H), 5.24 (s, 1 H), 4.62 (dd, J = 10.6, 4.7 Hz, 1 H), 2.19 (d, J = 11.1 Hz, 1 H), 2.06–1.87 (m, 4 H), 1.76–1.65 (m, 6 H), 1.59–1.48 s (m, 4 H), 1.36–1.24 (m, 10 H), 1.08 (s, 3 H), 0.97 (d, J = 8.3 Hz, 4 H), 0.92 (d, J = 7.8 Hz, 6 H), 0.86 (d, J = 6.0 Hz, 4 H), 0.78 (s, 3 H). ^13^C NMR (100 MHz, CDCl_3_) δ 183.3, 164.5, 138.2, 125.8, 85.1, 65.0, 55.5, 52.7, 48.1, 47.6, 42.1, 39.7, 39.2, 39.0, 38.3, 37.1, 36.9, 33.0, 30.8, 29.9, 28.1, 24.2, 23.8, 23.5, 23.3, 22.8, 21.3, 18.2, 17.3, 17.2, 16.7, 15.7. ESI-MS m/z 565.2 [M-H]^−^.

(*1S,2R,4R,6aS,6bR,10S,12aR*)-*10*-((*4-bromo-2-fluorobenzoyl) oxy*)-*1,2,6a,6b,9,9,12a-heptamethyl-1,2,3,4,4a,5,6,6a,6b,7,8,8a,9,10,11,12,12a,12b,13,14b-icosahydropicene-4-carboxylic acid* (**UA-04**, C_37_H_50_BrFO_4_). According to the general procedure, UA was treated with 4-Bromo-2-fluorobenzoylchloride at room temperature overnight, and then purified on a silica gel column with petroleum ether/ethyl acetate (v/v 10:1) as the eluent to obtain compound UA-04 (R_f_ = 0.48). Yield: 58%; white powder; mp: 276–277 °C; ^1^H NMR (400 MHz, CDCl_3_) δ 7.81 (t, J = 7.9 Hz, 1 H), 7.34 (t, J = 9.8 Hz, 2 H), 5.25 (s, 1 H), 4.74 (dd, J = 11.0, 4.6 Hz, 1 H), 2.20 (d, J = 11.2 Hz, 1 H), 2.07–1.98 (m, 1 H), 1.97–1.86 (m, 3 H), 1.81–1.67 (m, 5 H), 1.60–1.46 (m, 4 H), 1.42 (d, J = 5.7 Hz, 1 H), 1.39–1.29 (m, 4 H), 1.26 (s, 5 H), 1.10 (s, 3 H), 1.00 (s, 3 H), 0.96 (d, J = 5.9 Hz, 8 H), 0.87 (d, J = 6.3 Hz, 4 H), 0.80 (s, 3 H). ^13^C NMR (100 MHz, CDCl_3_) δ 183.2, 163.8, 163.8, 138.2, 133.3, 127.7, 125.9, 121.0, 120.7, 83.0, 55.5, 52.8, 48.1, 47.7, 42.1, 39.7, 39.2, 39.0, 38.5, 38.1, 37.1, 36.9, 33.1, 30.8, 29.9, 28.3, 28.2, 24.3, 23.8, 23.7, 23.5, 21.3, 18.4, 17.3, 17.2, 17.0, 15.7. ESI-MS m/z 655.2 [M-H]^−^.

(*1S,2R,4R,6aS,6bR,10S,12aR*)-*1,2,6a,6b,9,9,12a-heptamethyl*-*10*-((*4-methylpiperazine-1-carbonyl) oxy*)-*1,2,3,4,4a,5,6,6a,6b,7,8,8a,9,10,11,12,12a,12b,13,14b-icosahydropicene-4-carboxylic acid* (**UA-05**, C_36_H_58_N_2_O_4_). According to the general procedure, UA was treated with 4-Methyl-1-piperazinecarbonylchloride at room temperature overnight, and then purified on a silica gel column with petroleum ether/ethyl acetate (v/v 10:1) as the eluent to obtain compound UA-04 (R_f_ = 0.40). Yield: 45%; white powder; mp: 127–128 °C; ^1^H NMR (400 MHz, CDCl_3_) δ 5.26 (s, 1 H), 3.68–3.47 (m, 2 H), 3.47–3.32 (m, 2 H), 3.18 (dd, J = 10.1, 5.2 Hz, 1 H), 2.49–2.31 (m, 4 H), 2.28 (s, 3 H), 2.18 (d, J = 11.2 Hz, 1 H), 2.14–1.97 (m, 2 H), 1.96–1.82 (m, 3 H), 1.80–1.43 (m, 10 H), 1.39–1.22 (m, 4 H), 1.18–1.04 (m, 4 H), 0.98–0.91 (m, 7 H), 0.89 (s, 3 H), 0.84 (d, J = 6.3 Hz, 6 H), 0.75 (s, 3 H). ^13^C NMR (100 MHz, CDCl_3_) δ 173.2, 149.6, 137.9, 126.3, 79.0, 55.4, 54.7, 54.3, 52.9, 49.2, 47.7, 46.0, 45.1, 44.2, 42.3, 39.8, 39.2, 38.9, 38.8, 37.1, 36.0, 33.3, 30.6, 28.3, 28.0, 27.3, 24.4, 23.5, 23.4, 21.2, 18.4, 17.5, 17.0, 15.7, 15.6. ESI-MS m/z 581.4 [M-H]^−^.

### General procedure for the preparation of UA derivatives (UA-O-a~UA-O-j)

UA analogue compounds UA-O-a~UA-O-j were synthesized according to Fig. 3. UA was dissolved in acetone at 0 °C; Jones reagent was added to the reaction dropwise until the colour of the solution was stable to be slight brown, indicating that the Jones reagent was sufficient to oxidize the group of hydroxyl at the C-3 position to form active intermediate UA-O. UA-O-a~UA-O-j were prepared by Claisen Schmidt condensation of UA-O with various aldehydes in the presence of ethanolic potassium hydroxide at room temperature in good yield.

(*1S,2R,4R,6aS,6bR,12aR*)-*11*-((*Z*)-*benzylidene*)-*1,2,6a,6b,9,9,12a-heptamethyl-10-oxo-1,2,3,4,4a,5,6,6a,6b,7,8,8a,9,10,11,12,12a,12b,13,14b-icosahydropicene-4-carboxylic acid* (**UA-O-a**, C_37_H_50_O_3_). According to the general procedure, UA-O-a were prepared by Claisen Schmidt condensation of UA-O with benzaldehyde in the presence of ethanolic potassium hydroxide at room temperature in good yield. mp: 160–162 °C; ^1^H NMR (400 MHz, CDCl_3_) δ 7.61 (t, J = 12.0 Hz, 1 H), 7.29 (d, J = 11.7 Hz, 1 H), 7.23–7.13 (m, 4 H), 6.88 (dd, J = 14.9, 11.9 Hz, 1 H), 5.36 (s, 1 H), 2.92 (d, J = 24.9 Hz, 1 H), 2.35 (s, 3 H), 2.25 (d, J = 11.4 Hz, 1 H), 2.19–1.95 (m, 4 H), 1.88 (t, J = 12.0 Hz, 1 H), 1.78–1.64 (m, 4 H), 1.53 (d, J = 13.1 Hz, 2 H), 1.47–1.33 (m, 6 H), 1.29–1.20 (m, 3 H), 1.12 (t, 9 H), 0.98–0.89 (m, 9 H), 0.86 (s, 3 H).^13^C NMR (100 MHz, CDCl_3_) δ 207.3, 183.6, 138.5, 138.3, 137.7, 136.6, 135.8, 132.8, 130.8, 128.8, 126.3, 125.8, 125.7, 124.6, 53.4, 53.0, 48.3, 45.4, 45.3, 42.7, 42.4, 39.6, 39.3, 39.0, 36.9, 36.2, 32.4, 30.8, 29.7, 28.2, 24.3, 23.8, 23.6, 22.8, 21.3, 20.4, 19.9, 17.2, 17.0, 15.7. ESI-MS m/z 541.3 [M-H]^−^.

(*1S,2R,4R,6aS,6bR,12aR,Z*)-*1,2,6a,6b,9,9,12a*-*heptamethyl*-*10-oxo-11-*((*E) -3-phenylallylidene)-1,2,3,4,4a,5,6,6a,6b,7,8,8a,9,10,11,12,12a,12b,13,14b-icosahydropicene-4-carboxylic acid* (**UA-O-b**, C_39_H_52_O_3_). According to the general procedure, UA-O-b were prepared by Claisen Schmidt condensation of UA-O with phenylacrolein in the presence of ethanolic potassium hydroxide at room temperature in good yield. mp: 170–172 °C; ^1^H NMR (400 MHz, CDCl_3_) δ 7.68 (d, J = 7.5 Hz, 1 H), 7.50–7.05 (m, 6 H), 6.94 (dd, J = 15.1, 11.9 Hz, 1 H), 5.34 (s, 1 H), 2.95 (d, J = 16.4 Hz, 1 H), 2.24 (d, J = 11.3 Hz, 1 H), 2.16 (d, J = 16.9 Hz, 1 H), 2.11–1.96 (m, 3 H), 1.88 (t, J = 11.8 Hz, 1 H), 1.79–1.62 (m, 4 H), 1.53 (d, J = 14.4 Hz, 2 H), 1.47–1.30 (m, 6 H), 1.28–1.21 (m, 2 H), 1.16–1.08 (m, 9 H), 1.00–0.88 (m, 9 H), 0.85 (s, 3 H).^13^C NMR (100 MHz, CDCl_3_) δ 207.3, 183.8, 138.3, 137.0, 136.3, 134.9, 134.1, 134.0, 130.2, 129.7, 127.0, 125.8, 125.7, 53.4, 52.9, 48.3, 45.4, 45.3, 42.8, 42.4, 39.6, 39.3, 39.0, 36.8, 36.2, 32.3, 30.8, 29.7, 28.2, 24.3, 23.8, 23.6, 22.8, 21.3, 20.4, 17.2, 17.0, 15.7. ESI-MS m/z 567.3 [M-H]^−^.

(*1S,2R,4R,6aS,6bR,12aR,Z*)-*11*-((*E*)-*3*-(*4-fluorophenyl) allylidene*)-*1,2,6a,6b,9,9,12a-heptamethyl-10-oxo-1,2,3,4,4a,5,6,6a,6b,7,8,8a,9,10,11,12,12a,12b,13,14b-icosahydropicene-4-carboxylic acid* (**UA-O-c**, C_39_H_51_FO_3_). According to the general procedure, UA-O-c were prepared by Claisen Schmidt condensation of UA-O with (E)-3-(4-fluorophenyl)acrylaldehyde in the presence of ethanolic potassium hydroxide at room temperature in good yield. mp: 172–173 °C; ^1^H NMR (400 MHz, CDCl_3_) δ 7.47 (dt, J = 14.5, 7.3 Hz, 2 H), 7.22 (t, J = 10.9, 5.2 Hz, 1 H), 7.04 (t, J = 8.6 Hz, 2 H), 6.90–6.84 (m, 2 H), 5.33 (t, J = 14.7 Hz, 1 H), 2.91 (d, J = 17.9 Hz, 1 H), 2.25 (d, J = 11.1 Hz, 1 H), 2.14 (d, J = 15.5 Hz, 1 H), 2.11–1.99 (m, 3 H), 1.92–1.83 (m, 1 H), 1.75–1.66 (m, 4 H), 1.53 (d, J = 12.5 Hz, 2 H), 1.45–1.36 (m, 5 H), 1.29–1.23 (m, 3 H), 1.14–1.09 (m, 10 H), 0.97 (d, J = 6.2 Hz, 3 H), 0.92 (d, J = 8.9 Hz, 6 H), 0.85 (s, 3 H). ^13^C NMR (100 MHz, CDCl_3_) δ 207.3, 183.6, 163.1, 139.4, 138.3, 137.2, 133.1, 132.9, 129.0, 128.9, 125.7, 123.3, 116.1, 115.9, 53.3, 53.0, 48.3, 45.3, 45.2, 42.7, 42.4, 39.6, 39.3, 39.0, 36.8, 36.2, 32.4, 30.8, 29.7, 28.1, 24.3, 23.8, 23.6, 22.8, 21.3, 20.4, 17.2, 17.0, 15.7. ESI-MS m/z 585.4 [M-H]^−^.

(*1S,2R,4R,6aS,6bR,12aR*)-*1,2,6a,6b,9,9,12a*-*heptamethyl*-*11*-((*Z*)-*4-nitrobenzylidene*)-*10-oxo-1,2,3,4,4a,5,6,6a,6b,7,8,8a,9,10,11,12,12a,12b,13,14b-icosahydropicene-4-carboxylic acid* (**UA-O-d**, C_37_H_49_NO_5_). According to the general procedure, UA-O-d were prepared by Claisen Schmidt condensation of UA-O with p-nitrobenzaldehyde in the presence of ethanolic potassium hydroxide at room temperature in good yield. mp: 172–174 °C; ^1^H NMR (400 MHz, CDCl_3_) δ 8.27 (d, J = 8.5 Hz, 2 H), 7.56 (s, 1 H), 7.53 (s, 2 H), 5.27 (s, 1 H), 2.96 (d, J = 16.3 Hz, 1 H), 2.29 (d, J = 16.7 Hz, 1 H), 2.21 (d, J = 11.3 Hz, 1 H), 2.07–1.80 (m, 4 H), 1.78–1.60 (m, 4 H), 1.60–1.46 (m, 4 H), 1.45–1.23 (m, 6 H), 1.19–1.07 (m, 10 H), 0.98–0.93 (m, 3 H), 0.90 (d, J = 6.2 Hz, 3 H), 0.87 (s, 3 H), 0.82 (d, J = 6.8 Hz, 3 H).^13^C NMR (100 MHz, CDCl_3_) δ 207.4, 183.4, 147.4, 142.6, 138.4, 137.4, 134.8, 130.8, 125.4, 123.8, 53.4, 52.9, 48.2, 45.6, 45.4, 44.2, 42.4, 39.6, 39.3, 39.0, 36.8, 36.6, 32.2, 30.8, 29.7, 28.1, 24.2, 23.7, 23.6, 22.9, 21.3, 20.4, 17.2, 16.9, 15.6. ESI-MS m/z 586.4 [M-H]^−^.

(*1S,2R,4R,6aS,6bR,12aR*)-*11*-((*Z*)-*3*-*chlorobenzylidene*)-*1,2,6a,6b,9,9,12a-heptamethyl*-*10*-*oxo*-*1,2,3,4,4a,5,6,6a,6b,7,8,8a,9,10,11,12,12a,12b,13,14b-icosahydropicene-4-carboxylic acid* (**UA-O-e**, C_37_H_49_ClO_3_). According to the general procedure, UA-O-e were prepared by Claisen Schmidt condensation of UA-O with 3-Chlorobenzaldehyde in the presence of ethanolic potassium hydroxide at room temperature in good yield. mp: 150–151 °C; ^1^H NMR (400 MHz, CDCl_3_) δ 7.46–7.39 (m, 1 H), 7.39–7.32 (m, 2 H), 7.32–7.27 (m, 2 H), 5.29 (t, 1 H), 2.97 (d, J = 16.2 Hz, 1 H), 2.30–2.17 (m, 2 H), 2.08–1.83 (m, 4 H), 1.79–1.61 (m, 4 H), 1.59–1.23 (m, 10 H), 1.13 (s, 9 H), 1.07–1.00 (m, 1 H), 0.99–0.93 (m, 3 H), 0.90 (d, J = 7.3 Hz, 3 H), 0.86 (s, 3 H), 0.82 (s, 3 H).^13^C NMR (100 MHz, CDCl_3_) δ 207.6, 183.8, 138.1, 135.9, 135.2, 134.4, 131.2, 130.1, 129.7, 128.4, 127.9, 125.5, 53.2, 52.7, 48.1, 45.3, 45.3, 43.8, 42.2, 39.5, 39.1, 38.9, 36.7, 36.5, 32.1, 30.6, 29.5, 28.0, 24.1, 23.6, 23.5, 22.7, 21.1, 20.3, 17.1, 16.7, 15.4. ESI-MS m/z 575.2 [M-H]^−^.

(*1S,2R,4R,6aS,6bR,12aR,Z*)-*1,2,6a,6b,9,9,12a*-*heptamethyl*-*10*-*oxo-11*-((*6*-(*trifluoromethyl) pyridin-3-yl) methylene*)-*1,2,3,4,4a,5,6,6a,6b,7,8,8a,9,10,11,12,12a,12b,13,14b-icosahydropicene-4-carboxylic acid* (**UA-O-f**, C_37_H_48_F_3_NO_3_). According to the general procedure, UA-O-f were prepared by Claisen Schmidt condensation of UA-O with 6-(Trifluoromethyl)nicotinaldehydein the presence of ethanolic potassium hydroxide at room temperature in good yield. mp: 168–169 °C; ^1^H NMR (400 MHz, CDCl_3_) δ 8.83 (s, 1 H), 7.90 (d, J = 8.1 Hz, 1 H), 7.76 (d, J = 8.1 Hz, 1 H), 7.52 (s, 1 H), 5.30 (t, J = 3.2 Hz, 1 H), 3.00 (d, J = 15.4 Hz, 1 H), 2.34 (d, J = 17.3 Hz, 1 H), 2.24 (d, 1 H), 2.10–1.93 (m, 3 H), 1.88 (td, J = 13.3, 3.6 Hz, 1 H), 1.80–1.64 (m, 4 H), 1.59–1.48 (m, 4 H), 1.48–1.26 (m, 6 H), 1.19 (d, J = 6.2 Hz, 6 H), 1.15 (s, 3 H), 1.09–1.03 (m, 1 H), 0.98 (d, J = 6.0 Hz, 3 H), 0.93 (d, J = 6.3 Hz, 3 H), 0.90 (s, 3 H), 0.84 (s, 3 H).^13^C NMR (100 MHz, CDCl_3_) δ 207.1, 183.6, 150.8, 147.2, 138.5, 138.4, 138.1, 134.9, 131.9, 125.4, 121.6, 120.4, 53.3, 52.9, 48.3, 45.5, 45.4, 44.5, 42.4, 39.6, 39.3, 39.0, 36.8, 36.5, 32.2, 30.8, 29.7, 28.1, 24.2, 23.8, 23.6, 22.8, 21.3, 20.4, 17.2, 16.9, 15.7. ESI-MS m/z 610.3 [M-H]^−^.

(*1S,2R,4R,6aS,6bR,12aR,Z*)-*1,2,6a,6b,9,9,12a*-*heptamethyl*-*10*-*oxo*-*11*-(*pyrazin-2-ylmethylene*)-*1,2,3,4,4a,5,6,6a,6b,7,8,8a,9,10,11,12,12a,12b,13,14b-icosahydropicene-4-carboxylic acid* (**UA-O-g**, C_35_H_48_N_2_O_3_). According to the general procedure, UA-O-g were prepared by Claisen Schmidt condensation of UA-O with Pyrazine-2-carbaldehyde in the presence of ethanolic potassium hydroxide at room temperature in good yield. mp: 162–163  °C; ^1^H NMR (400 MHz, CDCl_3_) δ 8.72–8.60 (m, 2 H), 8.43 (d, J = 2.2 Hz, 1 H), 7.40 (s, 1 H), 5.29 (t, J = 11.1 Hz, 1 H), 3.54 (d, J = 15.0 Hz, 1 H), 2.43 (d, J = 14.8 Hz, 1 H), 2.23 (d, 1 H), 2.13–1.95 (m, 3 H), 1.94–1.83 (m, 1 H), 1.79–1.62 (m, 4 H), 1.58–1.46 (m, 4 H), 1.43–1.35 (m, 3 H), 1.34–1.23 (m, 4 H), 1.17 (s, 3 H), 1.13 (s, 6 H), 0.96 (d, J = 6.2 Hz, 3 H), 0.91 (d, J = 5.3 Hz, 3 H), 0.89 (s, 3 H), 0.83 (s, 3 H).^13^C NMR (100 MHz, CDCl_3_) δ 208.2, 183.0, 151.8, 147.6, 144.3, 142.7, 141.3, 138.2, 130.4, 125.8, 53.3, 53.0, 48.2, 45.6, 45.3, 44.9, 42.4, 39.6, 39.3, 39.0, 36.9, 36.3, 32.3, 30.8, 29.7, 28.2, 24.3, 23.8, 23.6, 22.7, 21.3, 20.5, 17.2, 16.9, 15.8. ESI-MS m/z 543.3 [M-H]^−^.

(*1S,2R,4R,6aS,6bR,12aR,Z*)-*1,2,6a,6b,9,9,12a*-*heptamethyl-*1*0-oxo-11*-(*thiazol-2-ylmethylene*)-*1,2,3,4,4a,5,6,6a,6b,7,8,8a,9,10,11,12,12a,12b,13,14b-icosahydropicene-4-carboxylic acid* (**UA-O-h**, C_34_H_47_NO_3_S). According to the general procedure, UA-O-h were prepared by Claisen Schmidt condensation of UA-O with thiazole-2-carbaldehyde in the presence of ethanolic potassium hydroxide at room temperature in good yield. mp: 148–149 °C; ^1^H NMR (400 MHz, CDCl_3_) δ 8.01 (d, J = 3.2 Hz, 1 H), 7.64 (s, 1 H), 7.50 (d, J = 5.3 Hz, 1 H), 5.33 (s, 1 H), 3.37 (d, J = 17.8 Hz, 1 H), 2.34 (d, J = 17.2 Hz, 1 H), 2.24 (d, J = 11.3 Hz, 1 H), 2.20–2.10 (m, 1 H), 2.09–1.96 (m, 2 H), 1.88 (td, J = 13.6, 3.9 Hz, 1 H), 1.81–1.65 (m, 4 H), 1.59–1.48 (m, 4 H), 1.44–1.24 (m, 7 H), 1.17 (s, 3 H), 1.12 (d, J = 7.2 Hz, 6 H), 0.96 (d, J = 6.1 Hz, 3 H), 0.92 (d, J = 3.5 Hz, 6 H), 0.84 (s, 3 H).^13^C NMR (100 MHz, CDCl_3_) δ 207.6, 183.6, 163.9, 144.6, 138.2, 137.7, 127.9, 125.8, 121.6, 53.1, 52.9, 48.2, 45.5, 45.4, 45.4, 42.4, 39.6, 39.3, 39.0, 36.9, 36.3, 32.2, 30.8, 29.9, 28.2, 24.3, 23.8, 23.6, 22.6, 21.3, 20.5, 17.2, 16.8, 16.1. ESI-MS m/z 548.3 [M-H]^−^.

(*1S,2R,4R,6aS,6bR,12aR*)-*1,2,6a,6b,9,9,12a*-*heptamethyl*-*10*-*oxo-11*-((*Z*)-*4*-(*trifluoromethyl) benzylidene*)-*1,2,3,4,4a,5,6,6a,6b,7,8,8a,9,10,11,12,12a,12b,13,14b-icosahydropicene-4-carboxylic acid* (**UA-O-i**, C_38_H_49_F_3_O_3_). According to the general procedure, UA-O-i were prepared by Claisen Schmidt condensation of UA-O with p-Trifluoromethylbenzaldehyde in the presence of ethanolic potassium hydroxide at room temperature in good yield. mp: 171–172 °C; ^1^H NMR (400 MHz, CDCl_3_) δ 7.67 (d, J = 8.2 Hz, 2 H), 7.51 (d, J = 8.5 Hz, 3 H), 5.27 (t, J = 3.2 Hz, 1 H), 2.98 (d, J = 16.3 Hz, 1 H), 2.34–2.14 (m, 2 H), 2.09–1.97 (m, 1 H), 1.94 (dd, J = 8.6, 3.2 Hz, 2 H), 1.86 (td, J = 13.7, 4.0 Hz, 1 H), 1.78–1.61 (m, 4 H), 1.59–1.46 (m, 4 H), 1.46–1.18 (m, 6 H), 1.14 (d, J = 7.9 Hz, 9 H), 1.08–0.99 (m, 1 H), 0.96 (d, J = 6.1 Hz, 3 H), 0.90 (d, J = 5.7 Hz, 3 H), 0.87 (s, 3 H), 0.81 (s, 3 H). ^13^C NMR (100 MHz, CDCl_3_) δ 207.6, 183.7, 139.6, 139.6, 138.3, 136.0, 135.8, 130.4, 125.6, 125.50, 125.46, 53.4, 52.8, 48.2, 45.5, 45.4, 44.1, 42.4, 39.6, 39.3, 39.0, 36.8, 36.6, 32.2, 30.8, 29.7, 28.1, 24.2, 23.7, 23.6, 22.9, 21.3, 20.4, 17.2, 16.9, 15.6. ESI-MS m/z 609.4 [M-H]^−^.

(*1S,2R,4R,6aS,6bR,12aR*)-*1,2,6a,6b,9,9,12a*-*heptamethyl-11*-((*Z*)-*4*-*methylbenzylidene*)-*10*-*oxo*-*1,2,3,4,4a,5,6,6a,6b,7,8,8a,9,10,11,12,12a,12b,13,14b-icosahydropicene-4-carboxylic acid* (**UA-O-j**, C_38_H_52_O_3_). According to the general procedure, UA-O-j were prepared by Claisen Schmidt condensation of UA-O with p-methyl benzaldehyde in the presence of ethanolic potassium hydroxide at room temperature in good yield. mp: 142–143 °C; ^1^H NMR (400 MHz, CDCl_3_) δ 7.53 (s, 1 H), 7.35 (d, J = 8.0 Hz, 2 H), 7.23 (d, J = 7.9 Hz, 2 H), 5.28 (s, 1 H), 3.03 (d, J = 16.2 Hz, 1 H), 2.38 (s, 3 H), 2.31–2.24 (m, 1 H), 2.22 (d, J = 11.4 Hz, 1 H), 2.09–1.93 (m, 3 H), 1.86 (td, J = 13.4, 3.8 Hz, 1 H), 1.78–1.61 (m, 4 H), 1.59–1.45 (m, 4 H), 1.45–1.33 (m, 4 H), 1.26 (s, 3 H), 1.14 (d, J = 3.3 Hz, 9 H), 0.96 (d, J = 6.0 Hz, 3 H), 0.91 (d, J = 6.4 Hz, 3 H), 0.87 (s, 3 H), 0.81 (s, 3 H). ^13^C NMR (100 MHz, CDCl_3_) δ 207.9, 183.8, 138.8, 138.2, 137.8, 133.3, 133.1, 130.6, 129.4, 125.8, 53.3, 52.9, 48.2, 45.5, 45.3, 44.3, 42.4, 39.6, 39.3, 39.0, 36.9, 36.4, 32.3, 30.8, 29.8, 28.2, 24.3, 23.8, 23.6, 22.8, 21.5, 21.3, 20.5, 17.2, 16.9, 15.6. ESI-MS m/z 555.4 [M-H]^−^.

### α-glucosidase inhibitory activity

The α-glucosidase inhibition assay was performed according to the method of Worawalai^32^ with a slight modification. The α-glucosidase enzyme (0.1 U/mL) and substrate (1 mM *p*-nitrophenyl-α-D-glucopyranoside) were dissolved in 0.1 M phosphate buffer, pH 6.8. 10 μL of each synthesized analogue (1 mg/mL in DMSO) was pre-incubated with 8 μL of α-glucosidase at 37 °C for 10 min. A 100 μL of substrate solution was then added to the reaction mixture, which was further incubated at 37 °C for 30 min. Then, the reaction was terminated by adding 100 μL of 1 M Na_2_CO_3_ solution. Enzymatic activity was quantified by measuring the absorbance at 405 nm with a use of a Multimodel Plate Reader (Infinite 200). The percentage of inhibition was calculated by using [(*A*_*0*_ − *A*_*1*_)/*A*_*0*_] × 100%, where *A*_*0*_ was the absorbance without the sample, and *A*_*1*_ was the absorbance with the sample. The IC_50_ value was determined from a plot of the percentage of inhibition versus the sample concentration. Acarbose was used as the standard control and the experiment was performed in duplicate.

### Molecular modeling

The molecular minimization of UA analogues were built by use of the Sybyl molecular modelling package, version 2.0 (Tripos, shanghai, China). All structures of tested analogues were minimized with the Tripos force field, and the hydrogen atoms were added. Powel optimized the energy gradient with the maximum times to be 1000 times when the energy convergence criterion reached 0.005 kcal/mol by using Gasteiger–Hückle charges. Ligand-protein docking was performed by the Surflex-Dock in Sybyl 2.0. The crystal structure of α-glucosidase was retrieved from RCSB Protein Data Bank (PDB: 1UOK). Biopolymer module was then used to repair the crystal structure of the protein termini treatment, fix side chain amides, residues and add charges. The potent UA analogues docking with the α-glucosidase selected catalytic pocket of acarbose as active site. The active pocket was formed by computing, the others being the default settings.

## Additional Information

**How to cite this article**: Wu, P.-P. *et al*. Synthesis and biological evaluation of novel ursolic acid analogues as potential α-glucosidase inhibitors. *Sci. Rep.* **7**, 45578; doi: 10.1038/srep45578 (2017).

**Publisher's note:** Springer Nature remains neutral with regard to jurisdictional claims in published maps and institutional affiliations.

## Supplementary Material

Supplementary Information

## Figures and Tables

**Table 1:** Inhibitory effects of UA ester derivatives against α-glucosidase from baker’s yeast.

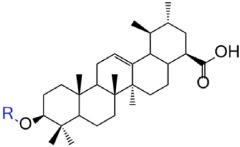			
Compound code	R	IC_50_^*a*^ (μM)	Binding free energy (kcal/mol)
**UA**	H	5.08 ± 0.70	−3.007
**UA-01**	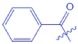	4.98 ± 0.21	−2.983
**UA-02**	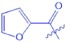	2.51 ± 0.02	−3.305
**UA-03**	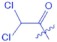	6.72 ± 0.33	−2.814
**UA-04**	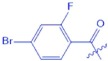	3.94 ± 0.10	−3.114
**UA-05**	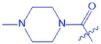	15.23 ± 0.47	−3.433
**Acarbose**^***b***^	—	569.43 ± 9.98	−9.134

**Table 2:** Inhibitory effects of UA analogues UA-O-a~UA-O-j against α-glucosidase from baker’s yeast.

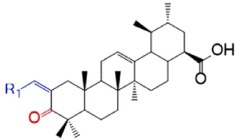			
Compound code	R_1_	IC_50_ (μM)	Binding free energy (kcal/mol)
**UA-O**	—	2.47 ± 0.14	—
**UA-O-a**	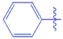	1.17 ± 0.09	−3.866
**UA-O-b**	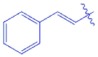	10.32 ± 0.55	−3.022
**UA-O-c**	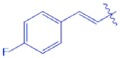	7.49 ± 0.29	−2.959
**UA-O-d**	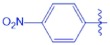	1.49 ± 0.14	−4.068
**UA-O-e**	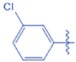	3.22 ± 0.78	−4.084
**UA-O-f**	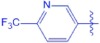	4.87 ± 0.41	−2.779
**UA-O-g**	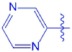	7.87 ± 0.34	−4.058
**UA-O-h**	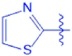	3.32 ± 0.17	−3.982
**UA-O-i**	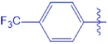	0.71 ± 0.04	−3.512
**UA-O-j**	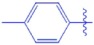	1.27 ± 0.20	−3.535

**Figure 1:**
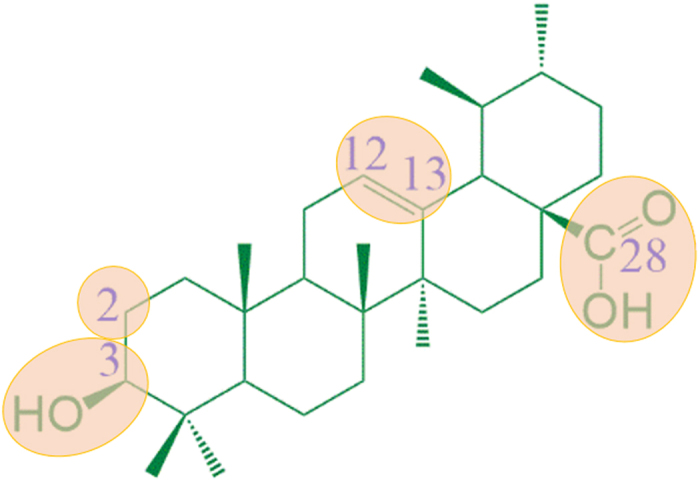
Chemical structure of ursolic acid.
