# Supplementary material for: Synthesis and biological evaluation of novel ursolic acid analogues as potential α-glucosidase inhibitors
Source: Sci Rep. 2017 Mar 30;7:45578. doi: 10.1038/srep45578 (PMC5372089; doi:10.1038/srep45578)

Synthesis and biological evaluation of novel ursolic acid analogues as potential α-glucosidase inhibitors

Pan-Pan Wu[1, 2, 3], Bing-Jie Zhang[1], Xi-Ping Cui[1], Yang Yang[1], Zheng-Yun Jiang[1], Zhi-Hong Zhou[1], Ying-Ying Zhong[1], Yu-Ying Mai[1], Zhong Ouyang[1], Hui-Sheng Chen[1], Jie Zheng[1], Su-Qing Zhao[1, 3*], Kun Zhang[1, 2, 3*]

1 *Department of Pharmaceutical Engineering, Faculty of Chemical Engineering and Light Industry, Guangdong University of Technology, Guangzhou, 510006, China*

2 *Faculty of Chemical ＆ Environmental Engineering, Wuyi University, Jiangmen, 529020, China*

3 *International Healthcare Innovation Institute (Jiangmen), Jiangmen, 529020, China*

**Corresponding Authors: sqzhao@gdut.edu.cn* (*S.-Q. Zhao), kzhang@gdut.edu.cn (K. Zhang)*

Supplementary Data

Copies of ^{1}H NMR and ^{13}C NMR spectra of compounds **UA-01~UA-05** and **UA-O-a~UA-O-j**.

Compound code: UA-01

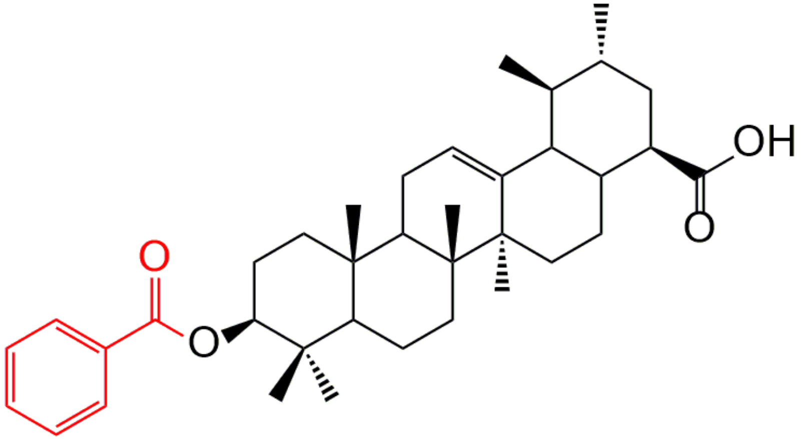

Figure 1. The structure of compound UA-01.

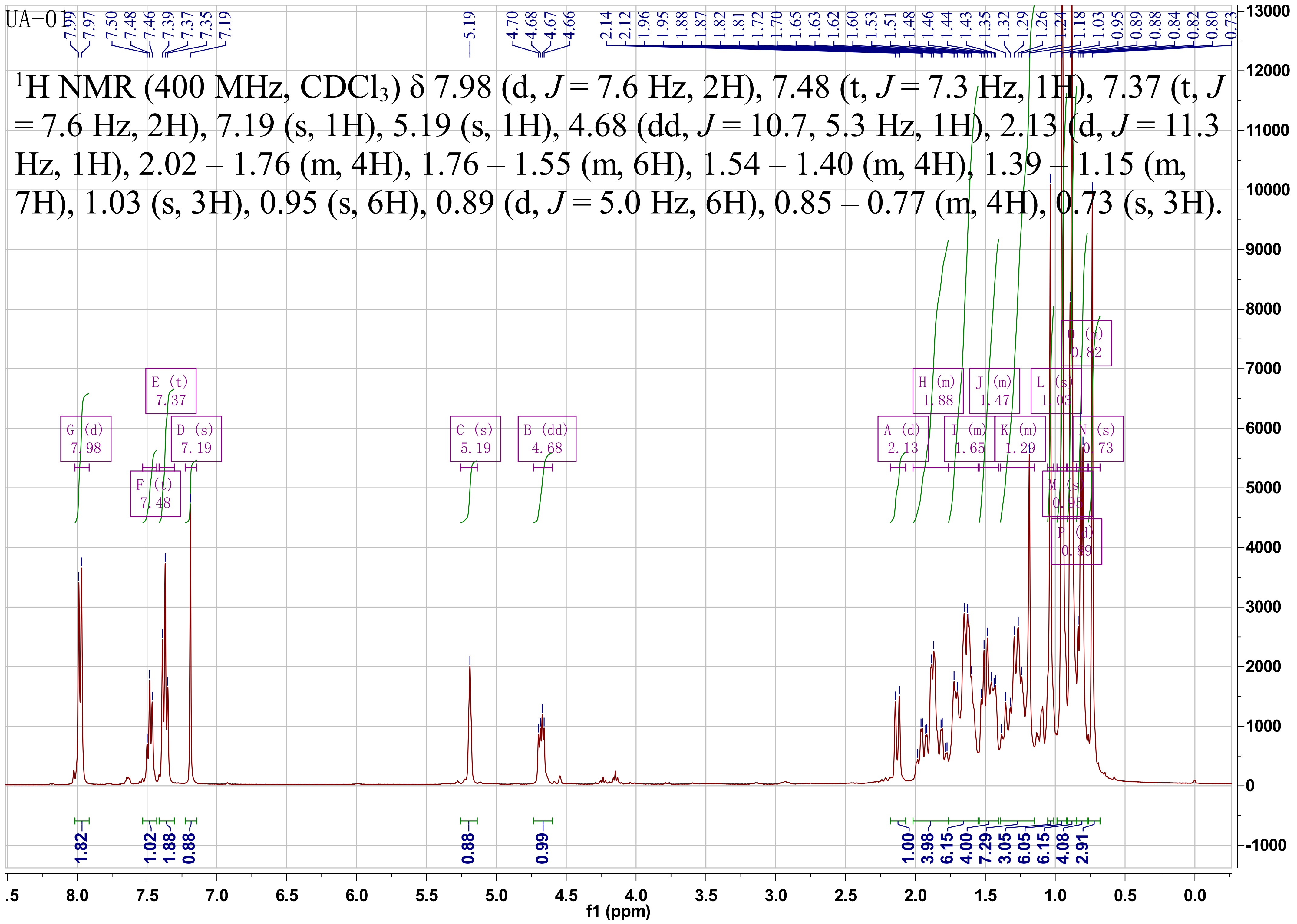
UA-01
1H NMR (400 MHz, CDCl3) δ 7.98 (d, J = 7.6 Hz, 2H), 7.48 (t, J = 7.3 Hz, 1H), 7.37 (t, J = 7.6 Hz, 2H), 7.19 (s, 1H), 5.19 (s, 1H), 4.68 (dd, J = 10.7, 5.3 Hz, 1H), 2.13 (d, J = 11.3 Hz, 1H), 2.02 – 1.76 (m, 4H), 1.76 – 1.55 (m, 6H), 1.54 – 1.40 (m, 4H), 1.39 – 1.15 (m, 7H), 1.03 (s, 3H), 0.95 (s, 6H), 0.89 (d, J = 5.0 Hz, 6H), 0.85 – 0.77 (m, 4H), 0.73 (s, 3H).
f1 (ppm)

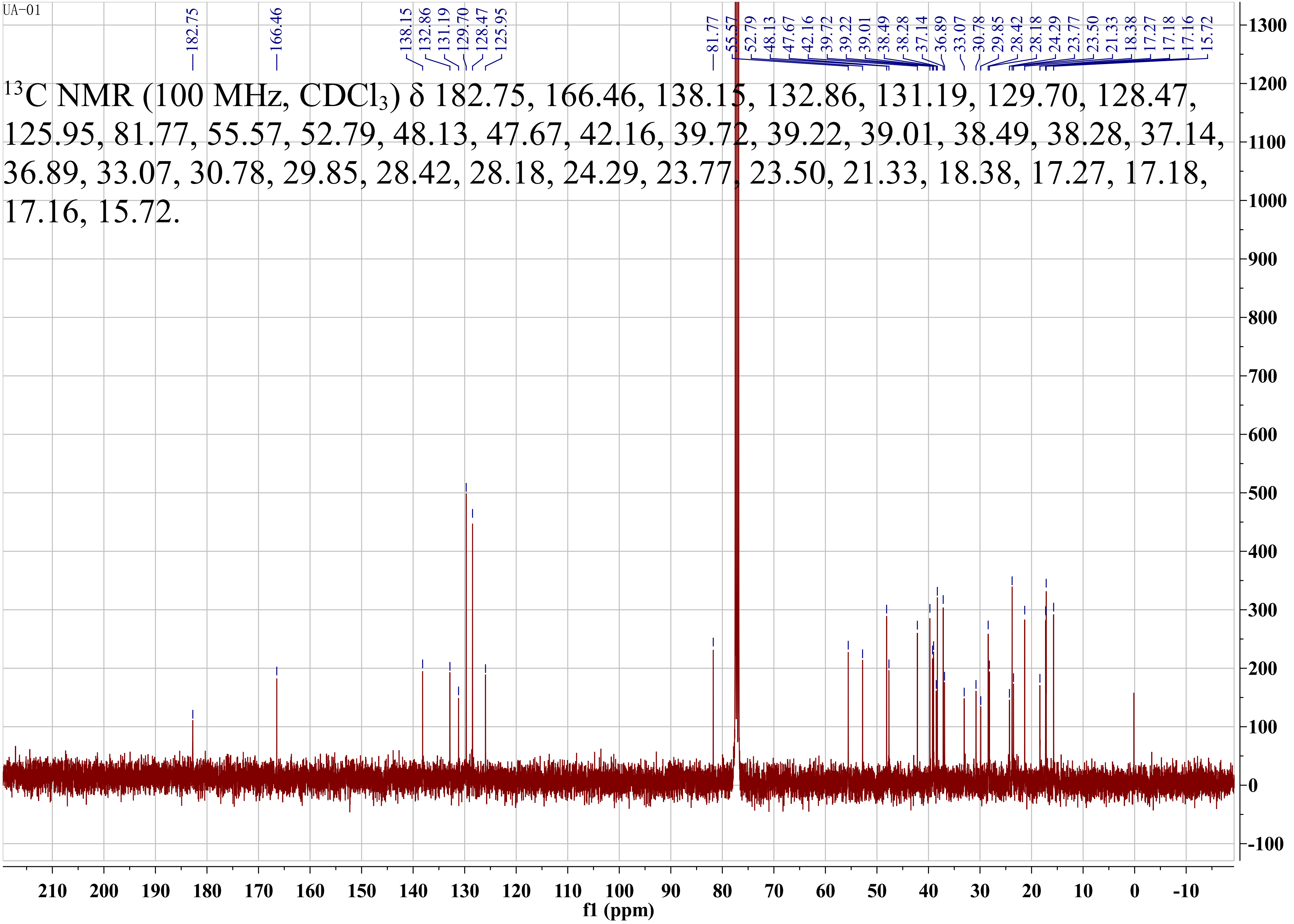

^{13}C NMR (100 MHz, $CDCl_3$) δ 182.75, 166.46, 138.15, 132.86, 131.19, 129.70, 128.47, 125.95, 81.77, 55.57, 52.79, 48.13, 47.67, 42.16, 39.72, 39.22, 39.01, 38.49, 38.28, 37.14, 36.89, 33.07, 30.78, 29.85, 28.42, 28.18, 24.29, 23.77, 23.50, 21.33, 18.38, 17.27, 17.18, 17.16, 15.72.

Compound code: UA-02

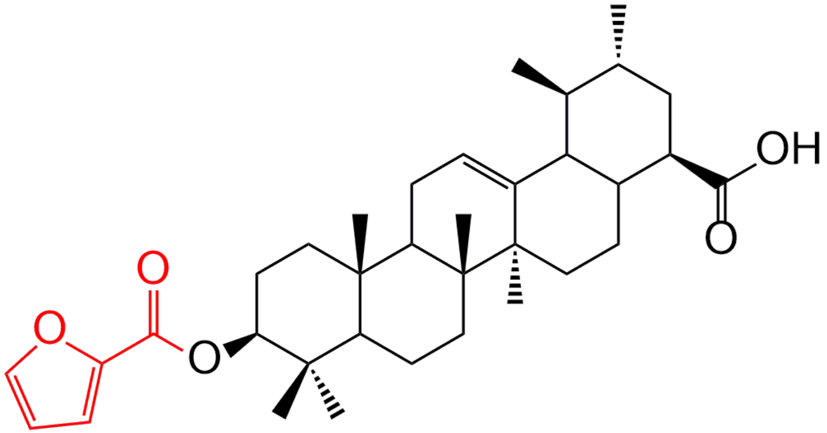

Figure 2. The structure of compound UA-02.

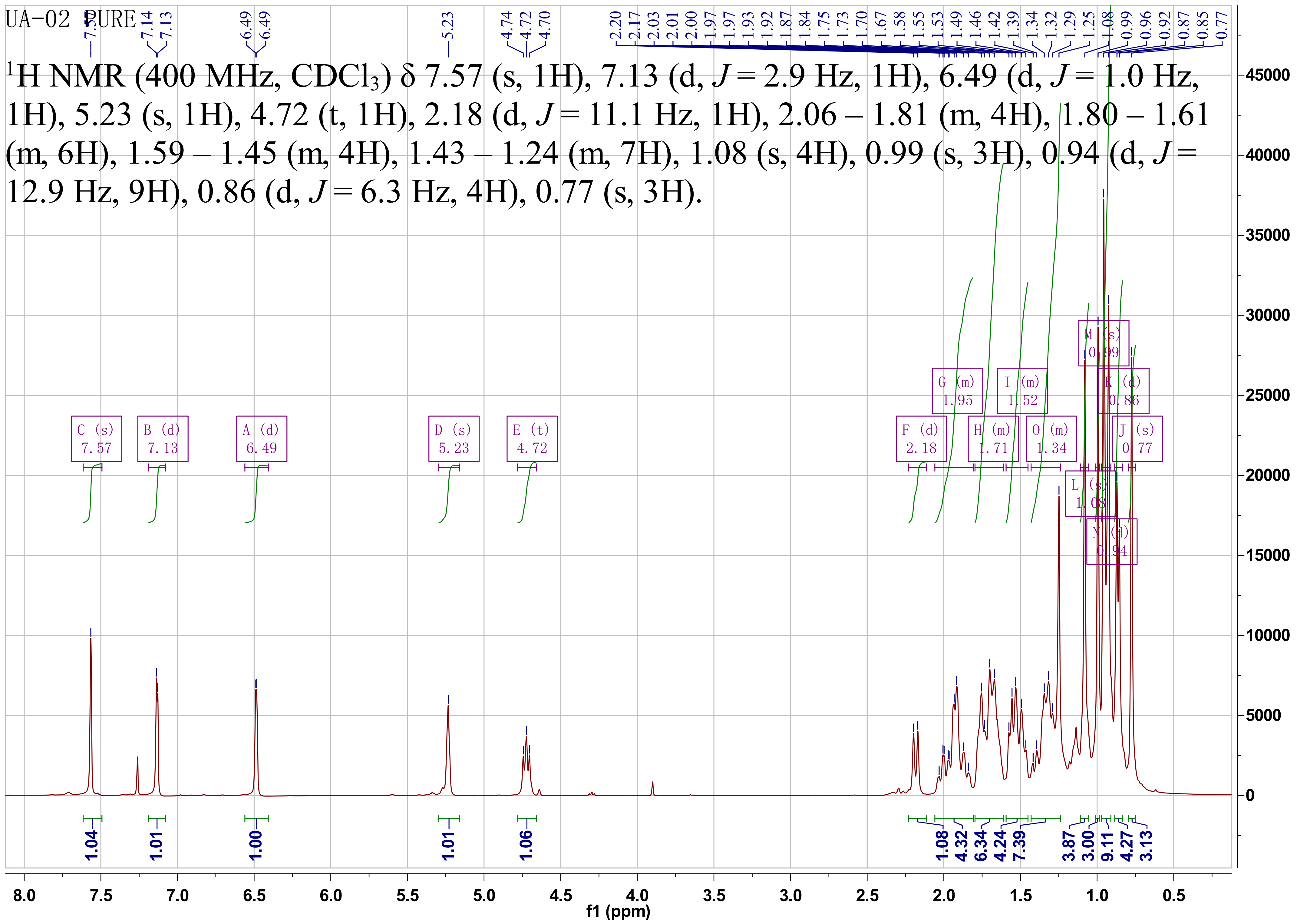

UA-02 PURE
^{1}H NMR (400 MHz, $CDCl_3$) δ 7.57 (s, 1H), 7.13 (d, J = 2.9 Hz, 1H), 6.49 (d, J = 1.0 Hz, 1H), 5.23 (s, 1H), 4.72 (t, 1H), 2.18 (d, J = 11.1 Hz, 1H), 2.06 – 1.81 (m, 4H), 1.80 – 1.61 (m, 6H), 1.59 – 1.45 (m, 4H), 1.43 – 1.24 (m, 7H), 1.08 (s, 4H), 0.99 (s, 3H), 0.94 (d, J = 12.9 Hz, 9H), 0.86 (d, J = 6.3 Hz, 4H), 0.77 (s, 3H).
C (s) 7.57
B (d) 7.13
A (d) 6.49
D (s) 5.23
E (t) 4.72
F (d) 2.18
G (m) 1.95
H (m) 1.71
I (m) 1.52
O (m) 1.34
L (s) 1.08
M (s) 0.99
N (d) 0.94
K (d) 0.86
J (s) 0.77
1.04
1.01
1.00
1.01
1.06
1.08
4.32
6.34
4.24
7.39
3.87
3.00
9.11
4.27
3.13
f1 (ppm)

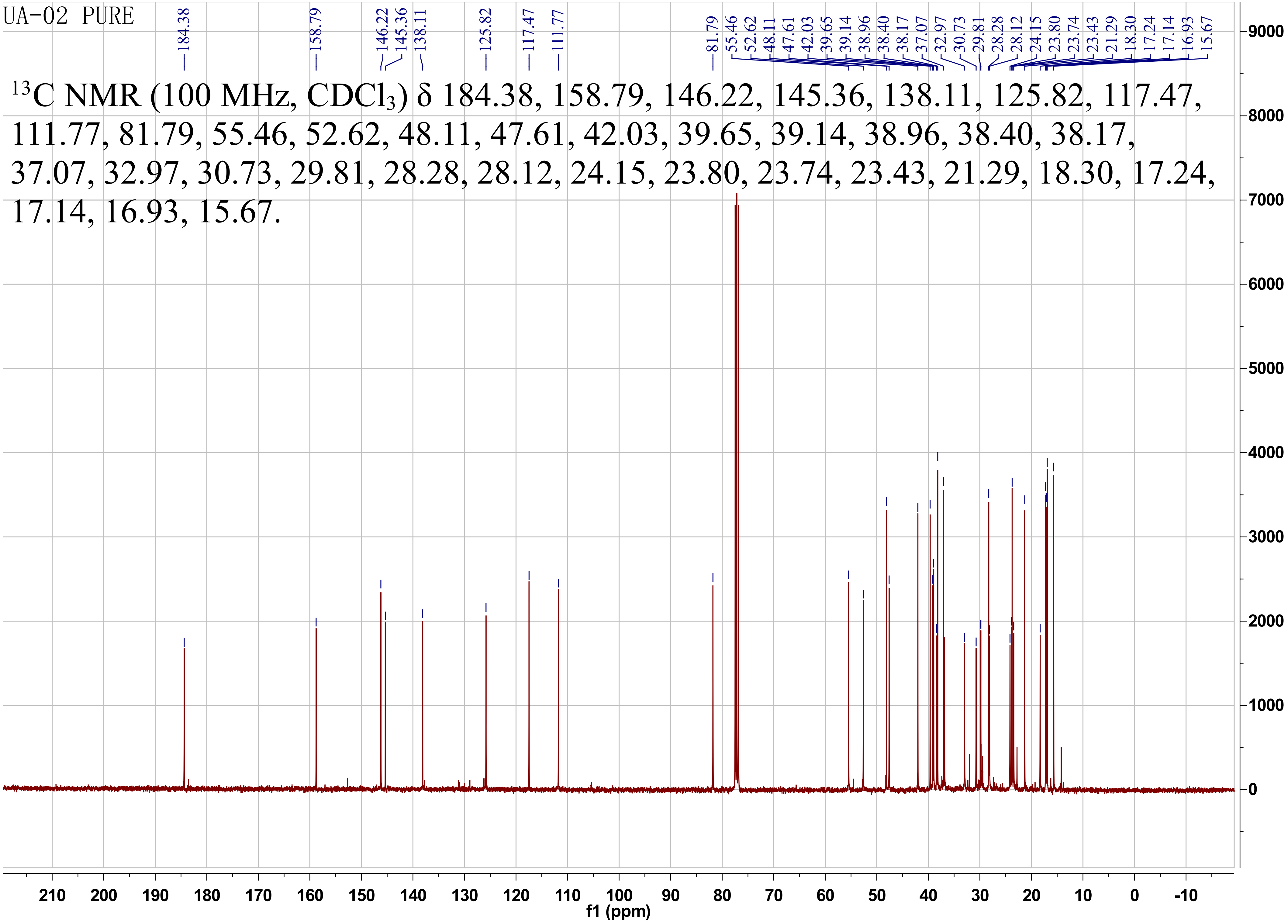

^{13}C NMR (100 MHz, $CDCl_3$) δ 184.38, 158.79, 146.22, 145.36, 138.11, 125.82, 117.47, 111.77, 81.79, 55.46, 52.62, 48.11, 47.61, 42.03, 39.65, 39.14, 38.96, 38.40, 38.17, 37.07, 32.97, 30.73, 29.81, 28.28, 28.12, 24.15, 23.80, 23.74, 23.43, 21.29, 18.30, 17.24, 17.14, 16.93, 15.67.

Compound code: UA-03

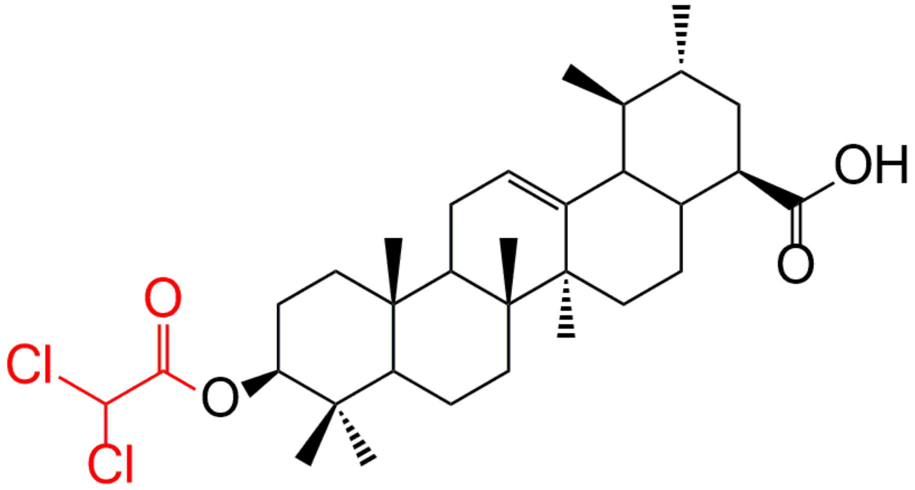

Figure 3. The structure of compound UA-03.

UA-03

^{1}H NMR (400 MHz, $CDCl_3$) δ 5.94 (s, 1H), 5.24 (s, 1H), 4.62 (dd, J = 10.6, 4.7 Hz, 1H), 2.19 (d, J = 11.1 Hz, 1H), 2.06 – 1.87 (m, 4H), 1.76 – 1.65 (m, 6H), 1.59 – 1.48 (m, 4H), 1.36 – 1.24 (m, 10H), 1.08 (s, 3H), 0.97 (d, J = 8.3 Hz, 5H), 0.92 (d, J = 7.8 Hz, 6H), 0.86 (d, J = 6.0 Hz, 4H), 0.78 (s, 3H).

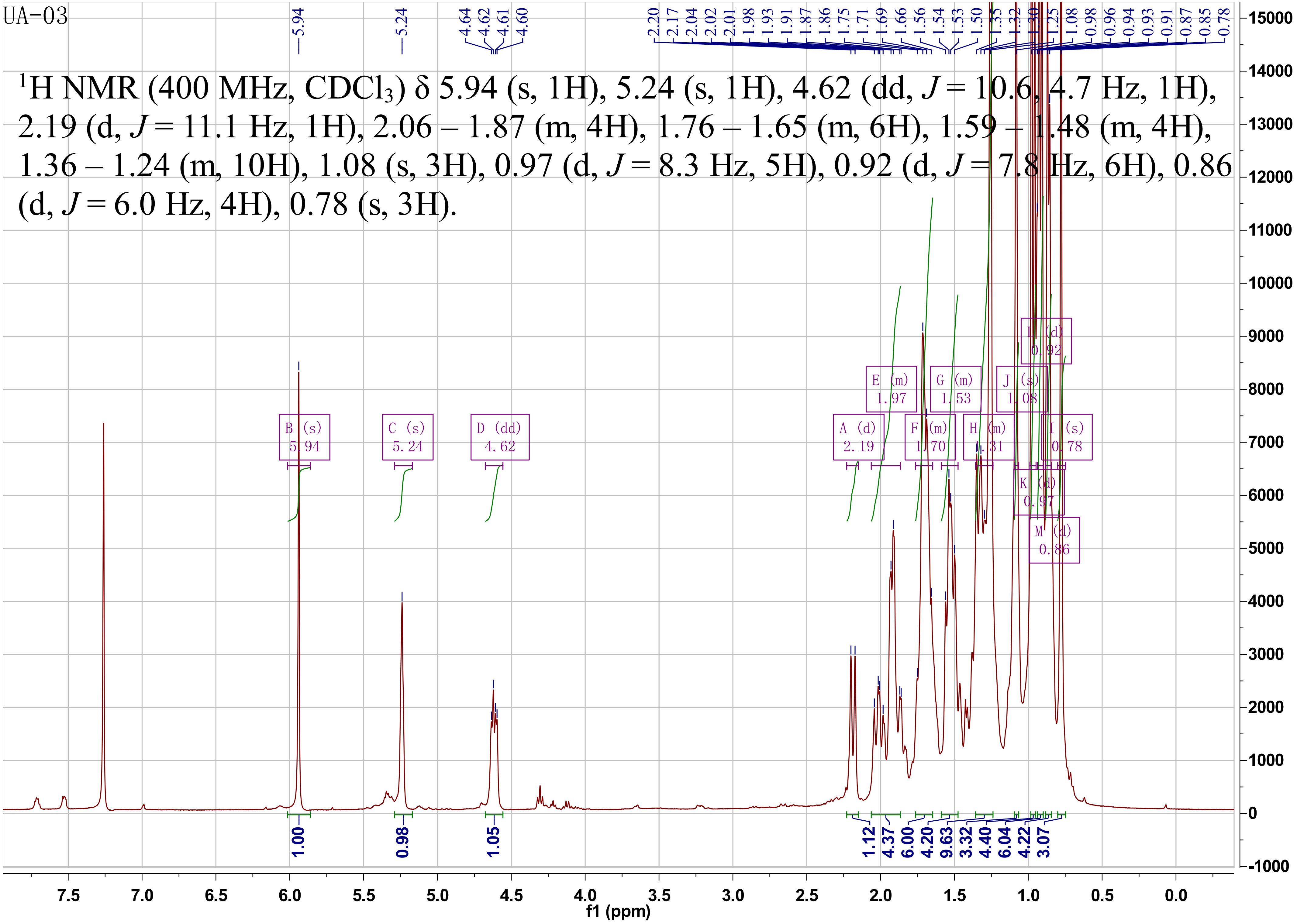

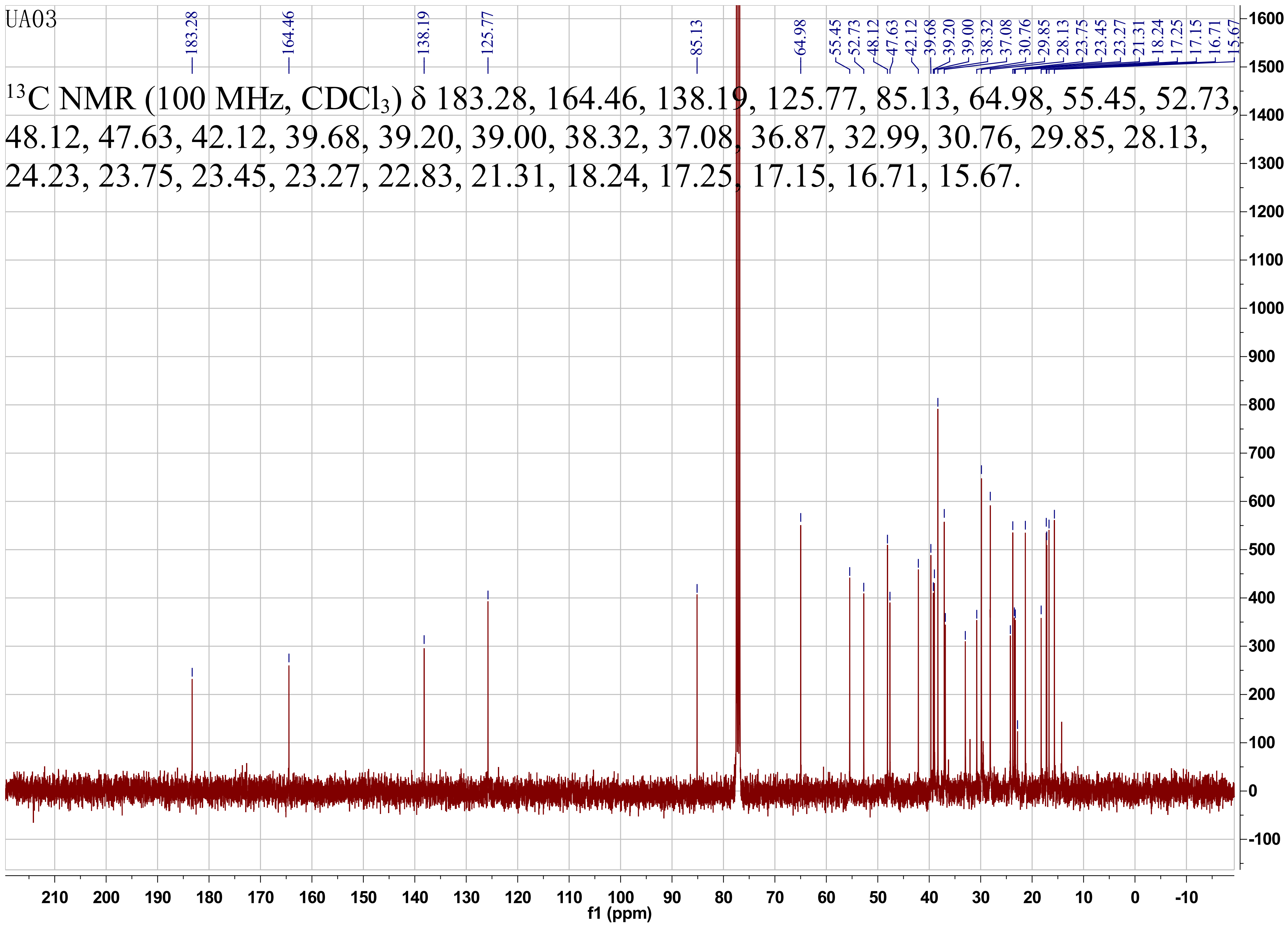

^{13}C NMR (100 MHz, $CDCl_3$) δ 183.28, 164.46, 138.19, 125.77, 85.13, 64.98, 55.45, 52.73, 48.12, 47.63, 42.12, 39.68, 39.20, 39.00, 38.32, 37.08, 36.87, 32.99, 30.76, 29.85, 28.13, 24.23, 23.75, 23.45, 23.27, 22.83, 21.31, 18.24, 17.25, 17.15, 16.71, 15.67.

Compound code: UA-04

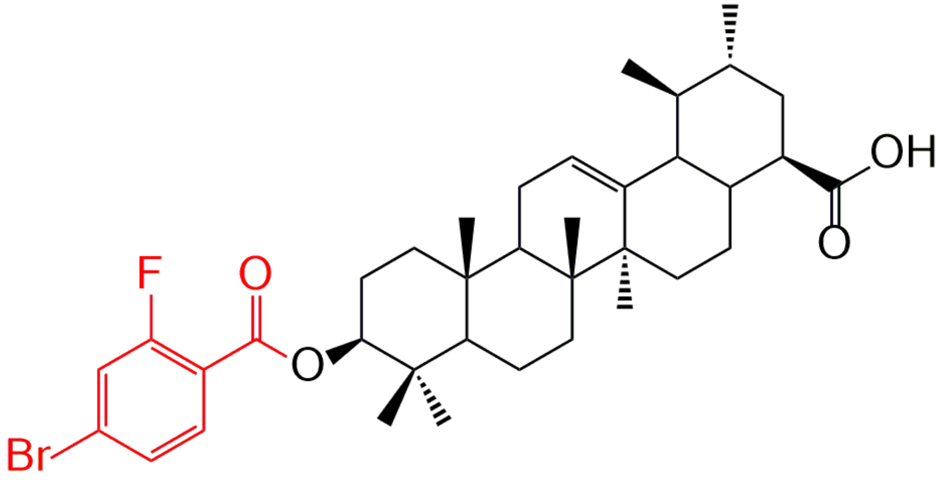

Figure 4. The structure of compound UA-04.

UA-09

^{1}H NMR (400 MHz, $CDCl_3$) δ 7.81 (t, *J* = 7.9 Hz, 1H), 7.34 (t, *J* = 9.8 Hz, 2H), 5.25 (s, 1H), 4.74 (dd, *J* = 11.0, 4.6 Hz, 1H), 2.20 (d, *J* = 11.2 Hz, 1H), 2.07 – 1.98 (m, 1H), 1.97 – 1.86 (m, 3H), 1.81 – 1.67 (m, 5H), 1.60 – 1.46 (m, 4H), 1.42 (d, *J* = 5.7 Hz, 1H), 1.39 – 1.29 (m, 4H), 1.26 (s, 5H), 1.10 (s, 3H), 1.00 (s, 3H), 0.96 (d, *J* = 5.9 Hz, 8H), 0.87 (d, *J* = 6.3 Hz, 4H), 0.80 (s, 3H).

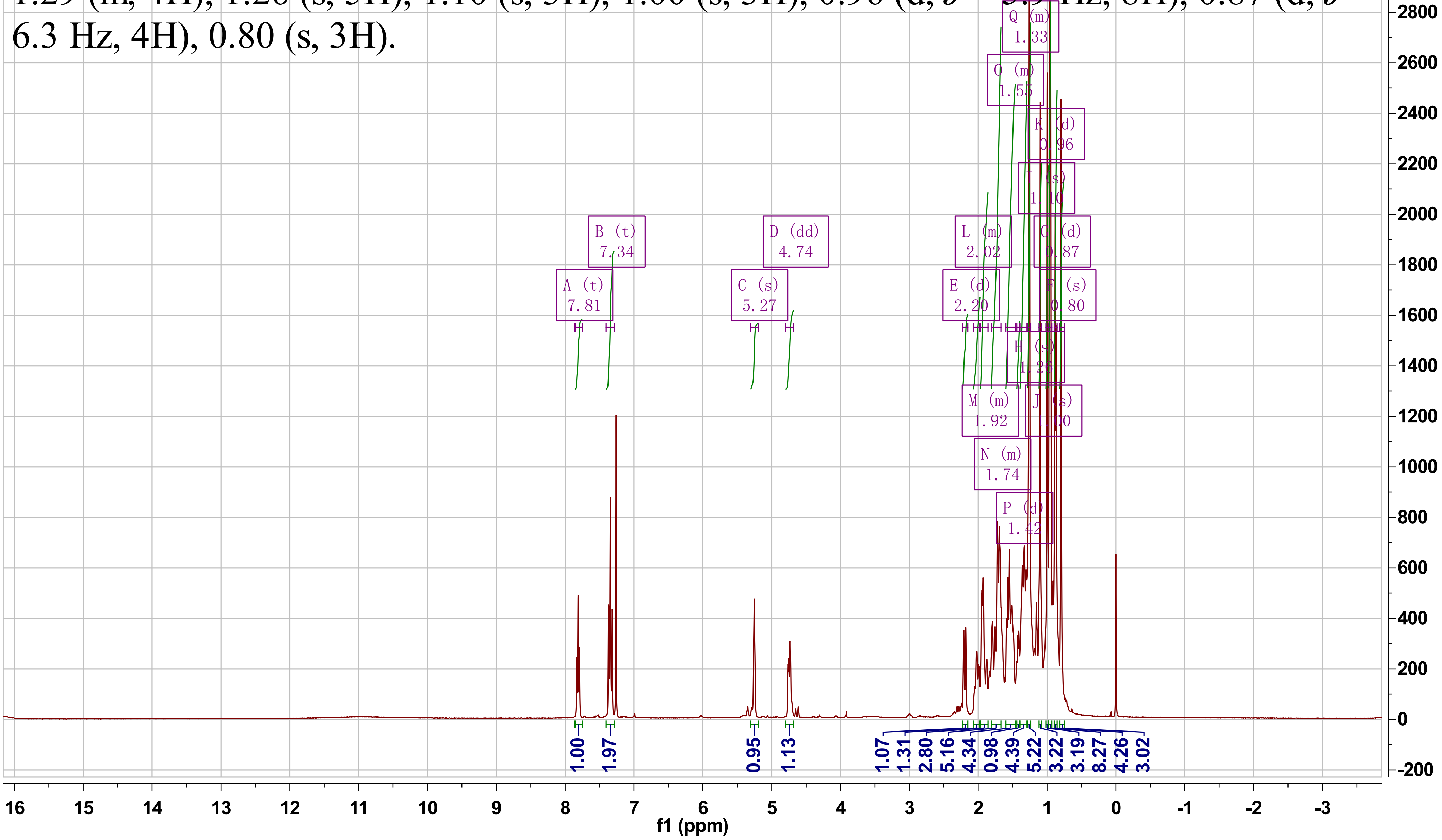

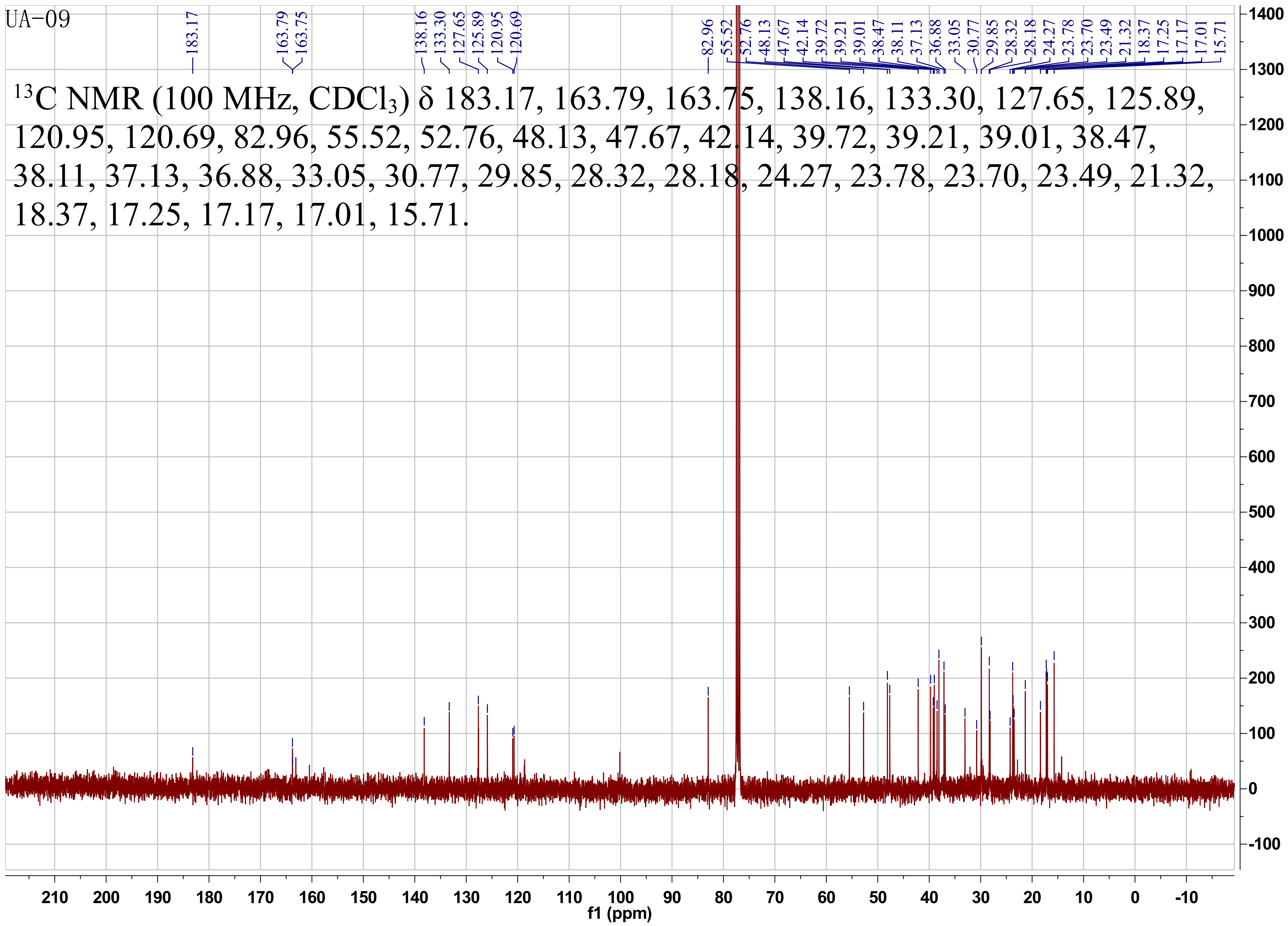

UA-09
183.17
163.79
163.75
138.16
133.30
127.65
125.89
120.95
120.69
82.96
55.52
52.76
48.13
47.67
42.14
39.72
39.21
39.01
38.47
38.11
37.13
36.88
33.05
30.77
29.85
28.32
28.18
24.27
23.78
23.70
23.49
21.32
18.37
17.25
17.17
17.01
15.71
^{13}C NMR (100 MHz, $CDCl_3$) δ 183.17, 163.79, 163.75, 138.16, 133.30, 127.65, 125.89, 120.95, 120.69, 82.96, 55.52, 52.76, 48.13, 47.67, 42.14, 39.72, 39.21, 39.01, 38.47, 38.11, 37.13, 36.88, 33.05, 30.77, 29.85, 28.32, 28.18, 24.27, 23.78, 23.70, 23.49, 21.32, 18.37, 17.25, 17.17, 17.01, 15.71.
f1 (ppm)

Compound code: UA-05

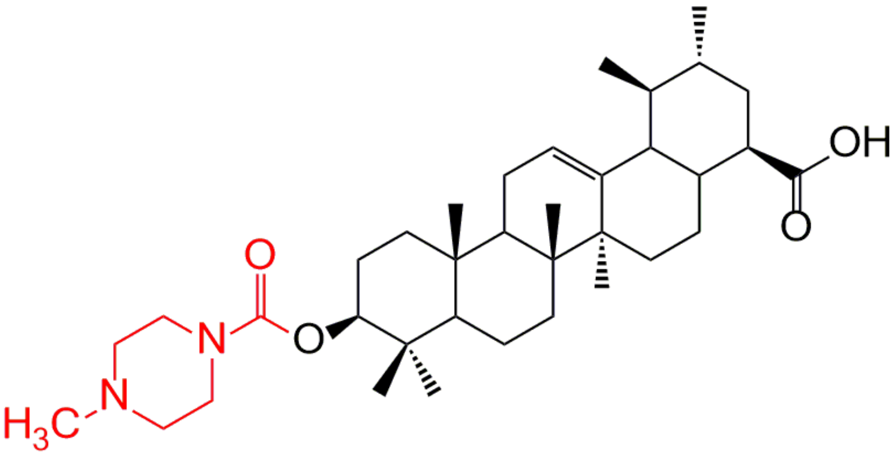

Figure 5. The structure of compound UA-05.

UA-06

^{1}H NMR (400 MHz, $CDCl_3$) δ 5.26 (s, 1H), 3.68 – 3.47 (m, 2H), 3.47 – 3.32 (m, 2H), 3.18 (dd, J = 10.1, 5.2 Hz, 1H), 2.49 – 2.31 (m, 4H), 2.28 (s, 3H), 2.18 (d, J = 11.2 Hz, 1H), 2.14 – 1.97 (m, 2H), 1.96 – 1.82 (m, 3H), 1.80 – 1.43 (m, 10H), 1.39 – 1.22 (m, 4H), 1.18 – 1.04 (m, 4H), 0.98 – 0.91 (m, 7H), 0.89 (s, 3H), 0.84 (d, J = 6.3 Hz, 6H), 0.75 (s, 3H).

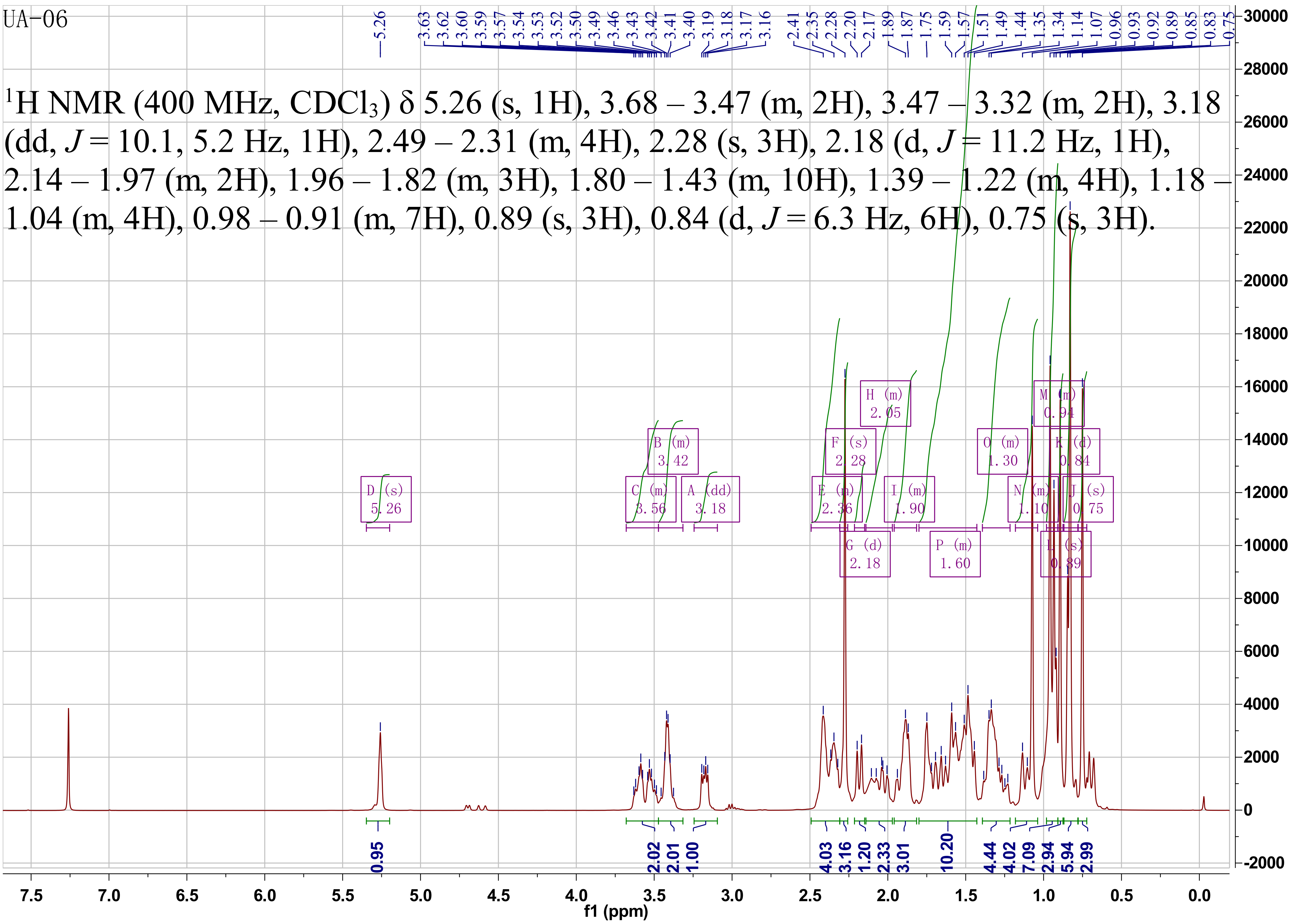

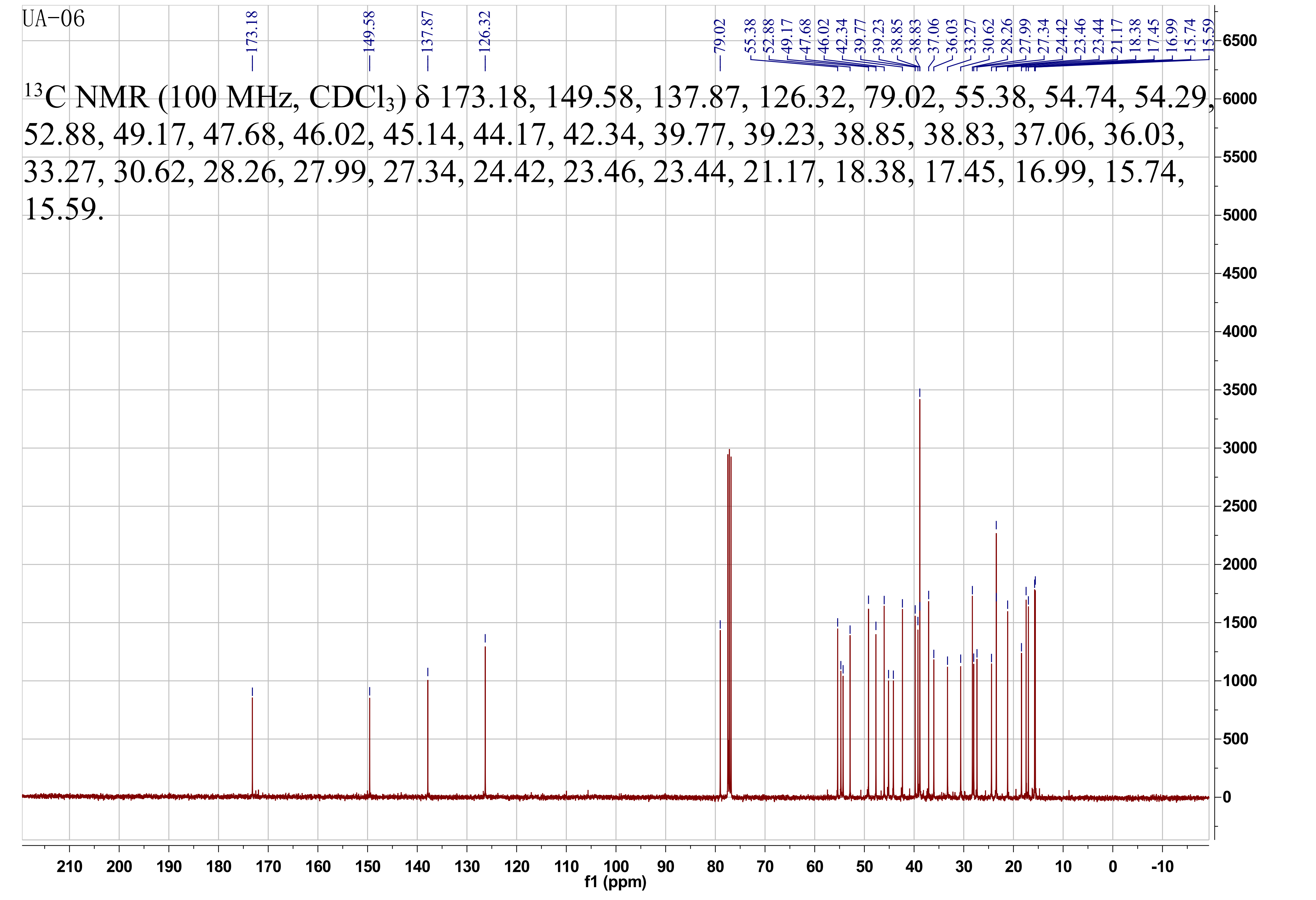

^{13}C NMR (100 MHz, $CDCl_3$) δ 173.18, 149.58, 137.87, 126.32, 79.02, 55.38, 54.74, 54.29, 52.88, 49.17, 47.68, 46.02, 45.14, 44.17, 42.34, 39.77, 39.23, 38.85, 38.83, 37.06, 36.03, 33.27, 30.62, 28.26, 27.99, 27.34, 24.42, 23.46, 23.44, 21.17, 18.38, 17.45, 16.99, 15.74, 15.59.

Compound code: UA-O-a

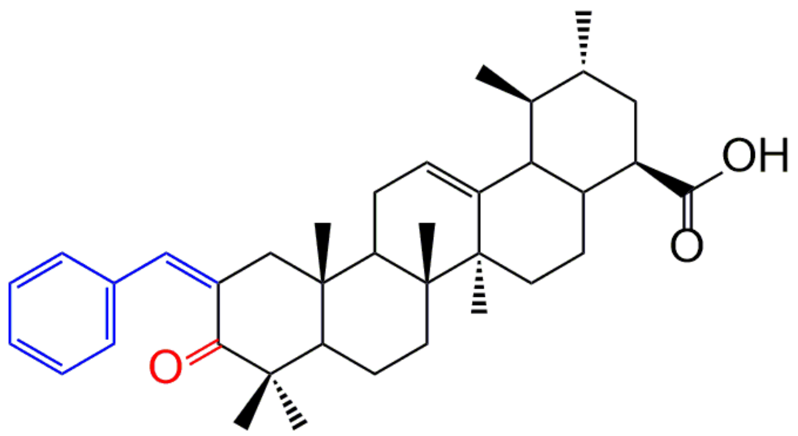

Figure 6. The structure of compound UA-O-a.

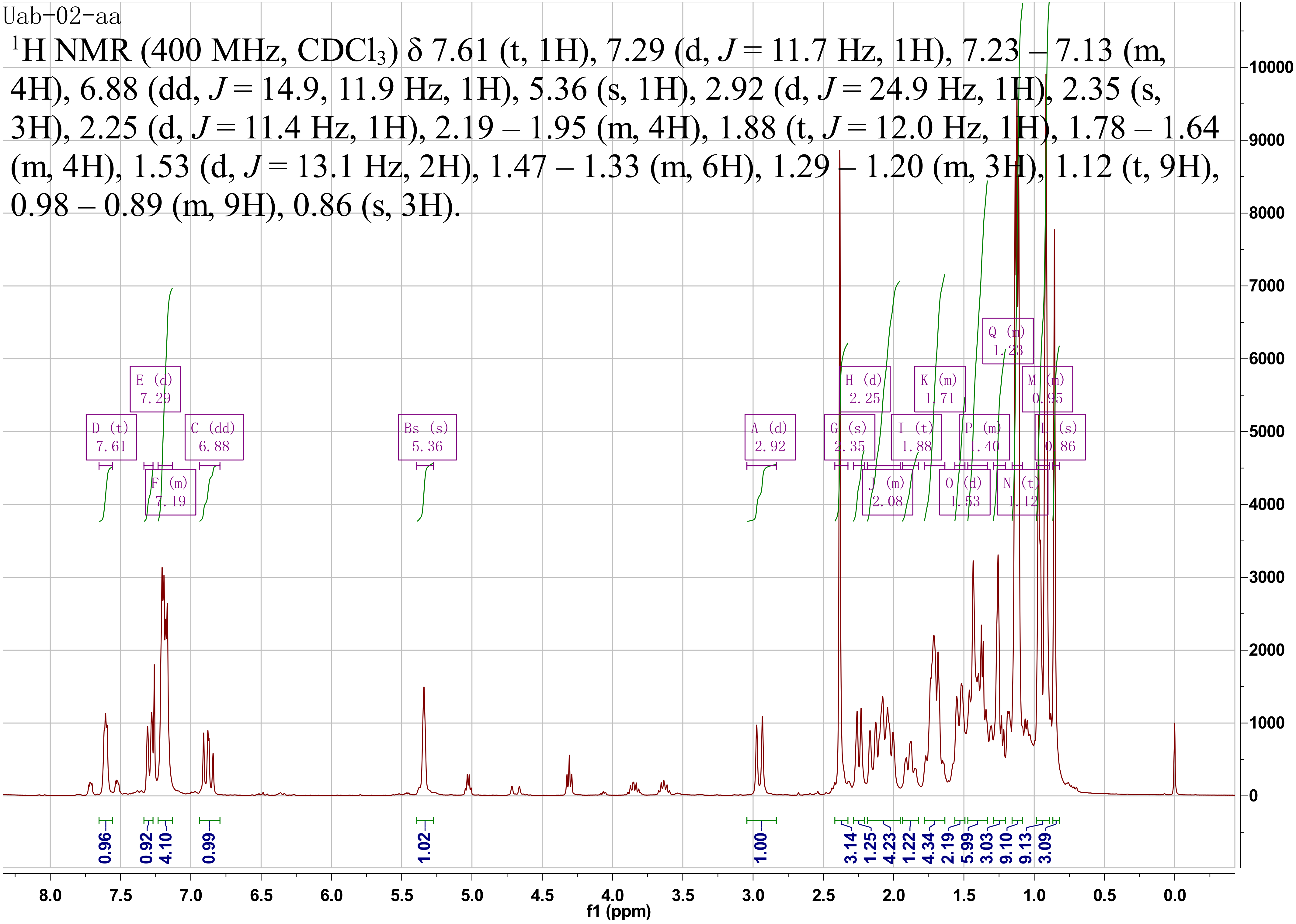
Uab-02-aa
¹H NMR (400 MHz, CDCl₃) δ 7.61 (t, 1H), 7.29 (d, J = 11.7 Hz, 1H), 7.23 – 7.13 (m, 4H), 6.88 (dd, J = 14.9, 11.9 Hz, 1H), 5.36 (s, 1H), 2.92 (d, J = 24.9 Hz, 1H), 2.35 (s, 3H), 2.25 (d, J = 11.4 Hz, 1H), 2.19 – 1.95 (m, 4H), 1.88 (t, J = 12.0 Hz, 1H), 1.78 – 1.64 (m, 4H), 1.53 (d, J = 13.1 Hz, 2H), 1.47 – 1.33 (m, 6H), 1.29 – 1.20 (m, 3H), 1.12 (t, 9H), 0.98 – 0.89 (m, 9H), 0.86 (s, 3H).
D (t) 7.61
E (d) 7.29
F (m) 7.19
C (dd) 6.88
Bs (s) 5.36
A (d) 2.92
G (s) 2.35
H (d) 2.25
J (m) 2.08
I (t) 1.88
K (m) 1.71
O (d) 1.53
P (m) 1.40
Q (m) 1.23
N (t) 1.12
M (m) 0.95
L (s) 0.86
0.96
0.92
4.10
0.99
1.02
1.00
3.14
1.25
4.23
1.22
4.34
2.19
5.99
3.03
9.10
9.13
3.09
f1 (ppm)

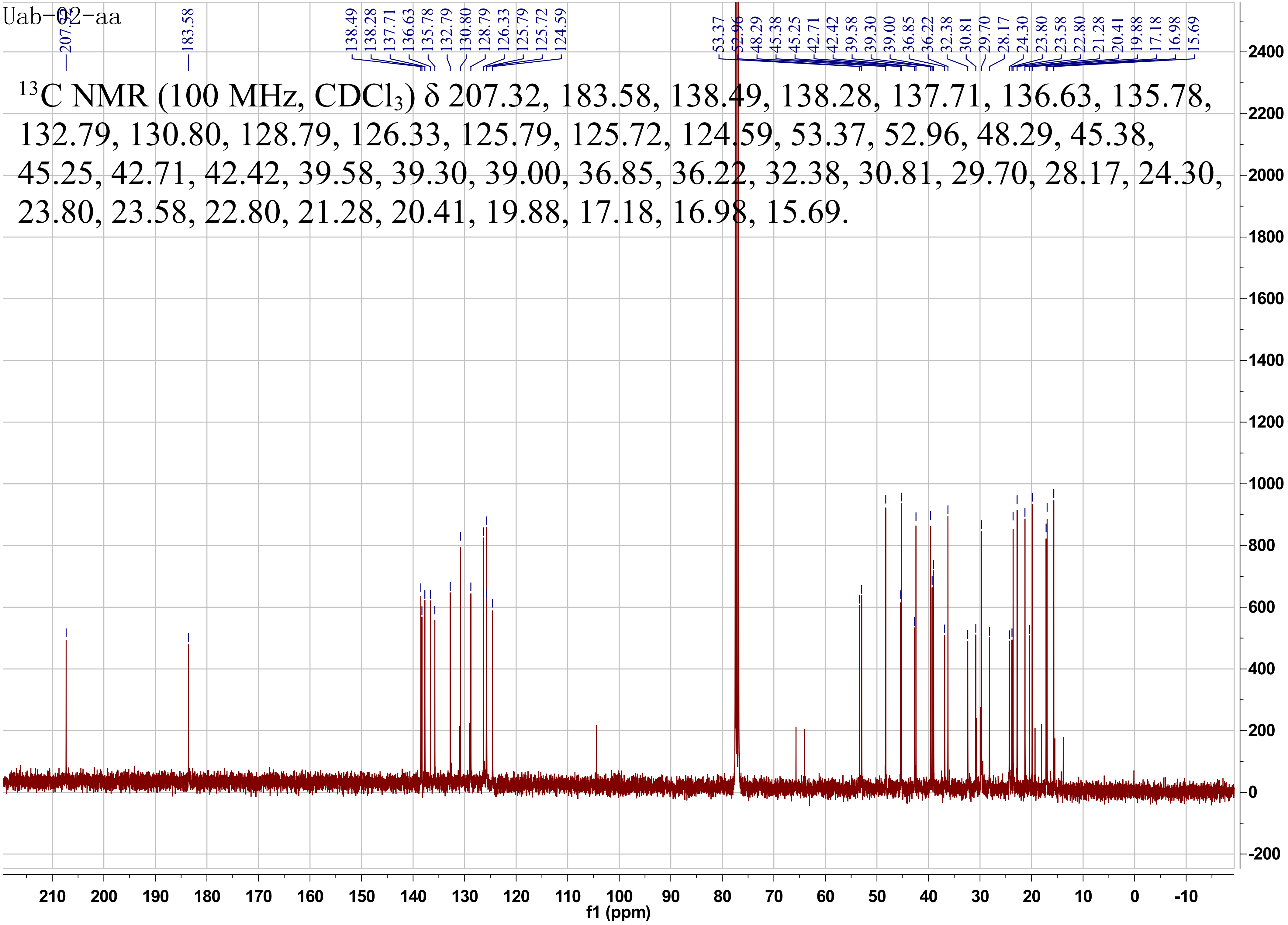

^{13}C NMR (100 MHz, $CDCl_3$) δ 207.32, 183.58, 138.49, 138.28, 137.71, 136.63, 135.78, 132.79, 130.80, 128.79, 126.33, 125.79, 125.72, 124.59, 53.37, 52.96, 48.29, 45.38, 45.25, 42.71, 42.42, 39.58, 39.30, 39.00, 36.85, 36.22, 32.38, 30.81, 29.70, 28.17, 24.30, 23.80, 23.58, 22.80, 21.28, 20.41, 19.88, 17.18, 16.98, 15.69.

Compound code: UA-O-b

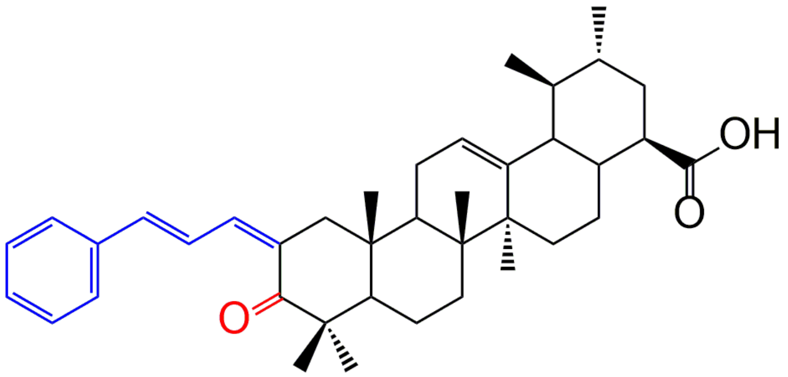

Figure 7. The structure of compound UA-O-b.

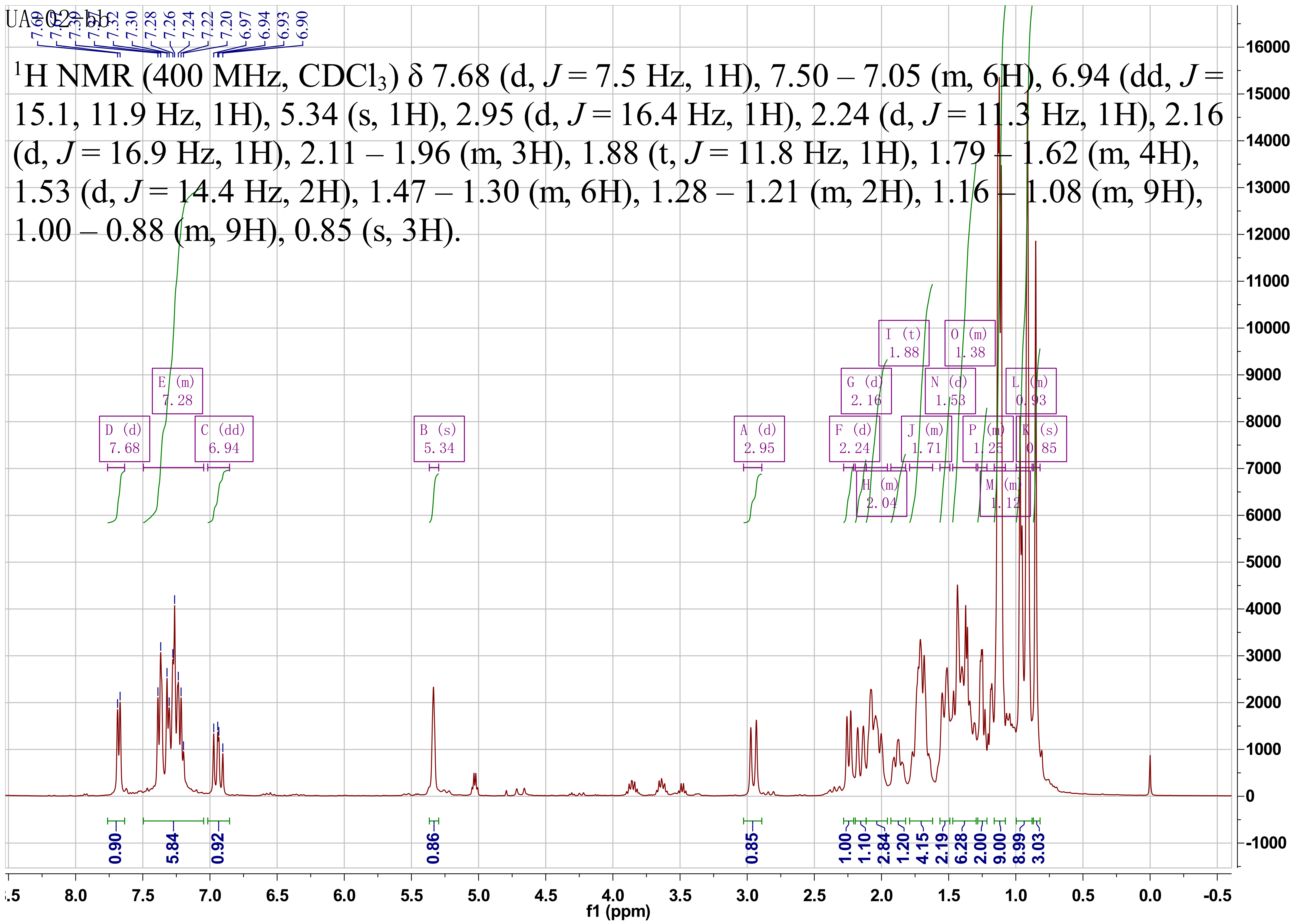
UA-02-bb
1H NMR (400 MHz, CDCl3) δ 7.68 (d, J = 7.5 Hz, 1H), 7.50 – 7.05 (m, 6H), 6.94 (dd, J = 15.1, 11.9 Hz, 1H), 5.34 (s, 1H), 2.95 (d, J = 16.4 Hz, 1H), 2.24 (d, J = 11.3 Hz, 1H), 2.16 (d, J = 16.9 Hz, 1H), 2.11 – 1.96 (m, 3H), 1.88 (t, J = 11.8 Hz, 1H), 1.79 – 1.62 (m, 4H), 1.53 (d, J = 14.4 Hz, 2H), 1.47 – 1.30 (m, 6H), 1.28 – 1.21 (m, 2H), 1.16 – 1.08 (m, 9H), 1.00 – 0.88 (m, 9H), 0.85 (s, 3H).
f1 (ppm)

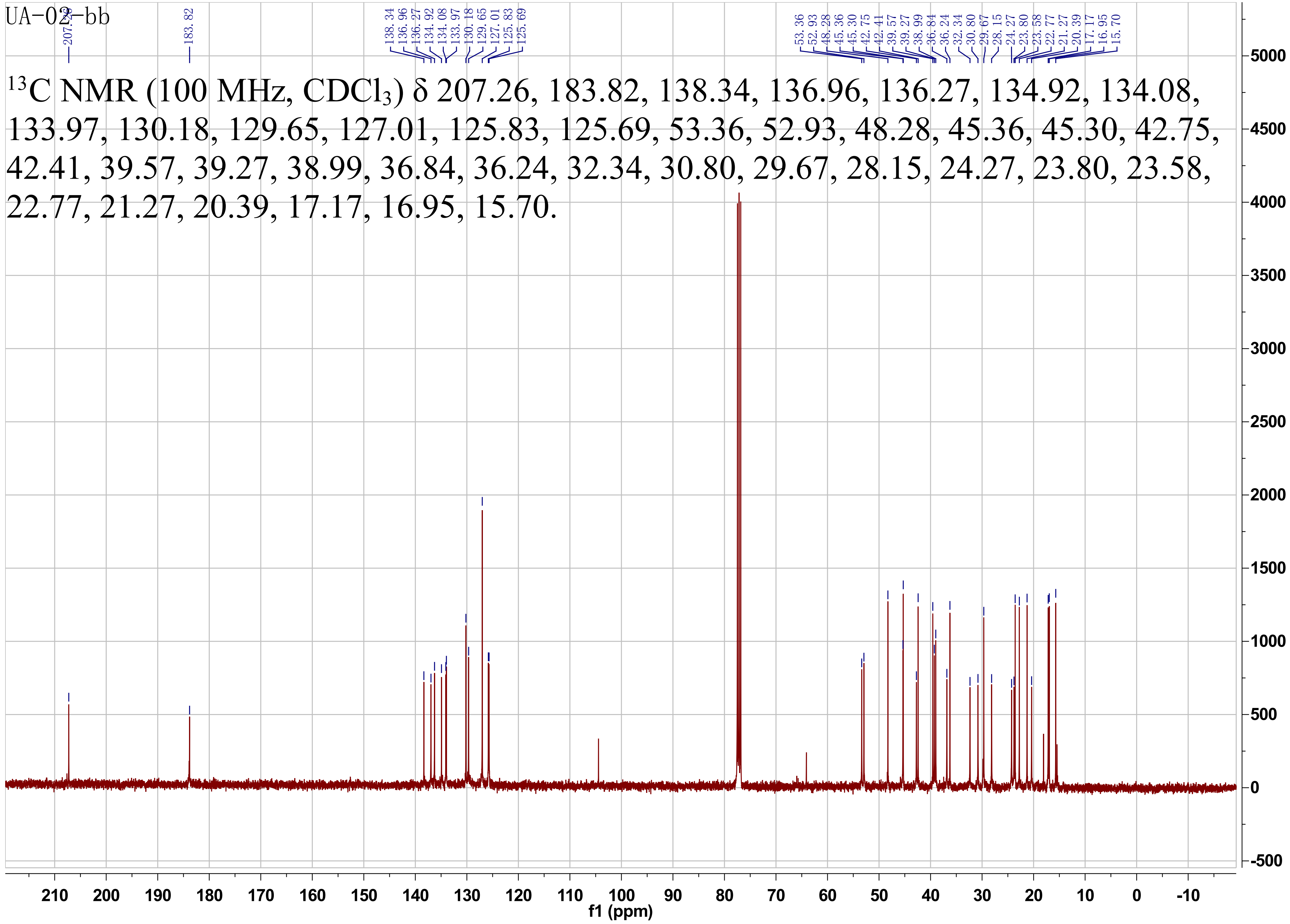
UA-02-bb
207.26
183.82
138.34
136.96
136.27
134.92
134.08
133.97
130.18
129.65
127.01
125.83
125.69
53.36
52.93
48.28
45.36
45.30
42.75
42.41
39.57
39.27
38.99
36.84
36.24
32.34
30.80
29.67
28.15
24.27
23.80
23.58
22.77
21.27
20.39
17.17
16.95
15.70
^{13}C NMR (100 MHz, $CDCl_3$) δ 207.26, 183.82, 138.34, 136.96, 136.27, 134.92, 134.08, 133.97, 130.18, 129.65, 127.01, 125.83, 125.69, 53.36, 52.93, 48.28, 45.36, 45.30, 42.75, 42.41, 39.57, 39.27, 38.99, 36.84, 36.24, 32.34, 30.80, 29.67, 28.15, 24.27, 23.80, 23.58, 22.77, 21.27, 20.39, 17.17, 16.95, 15.70.
f1 (ppm)

Compound code: UA-O-c

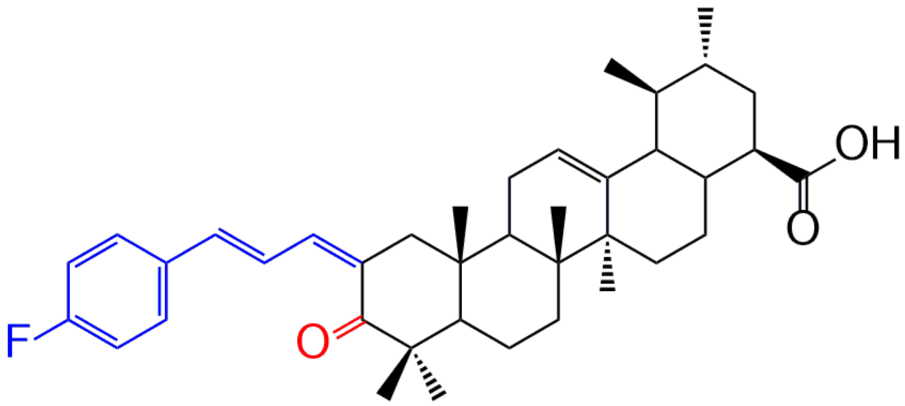

Figure 8. The structure of compound UA-O-c.

UA-02-dd

^{1}H NMR (400 MHz, $CDCl_3$) δ 7.47 (dt, J = 14.5, 7.3 Hz, 2H), 7.22 (t, J = 10.9, 5.2 Hz, 1H), 7.04 (t, J = 8.6 Hz, 2H), 6.90 – 6.84 (m, 2H), 5.33 (t, J = 14.7 Hz, 1H), 2.91 (d, J = 17.9 Hz, 1H), 2.25 (d, J = 11.1 Hz, 1H), 2.14 (d, J = 15.5 Hz, 1H), 2.11 – 1.99 (m, 3H), 1.92 – 1.83 (m, 1H), 1.75 – 1.66 (m, 4H), 1.53 (d, J = 12.5 Hz, 2H), 1.45 – 1.36 (m, 5H), 1.29 – 1.23 (m, 3H), 1.14 – 1.09 (m, 10H), 0.97 (d, J = 6.2 Hz, 3H), 0.92 (d, J = 8.9 Hz, 6H), 0.85 (s, 3H).

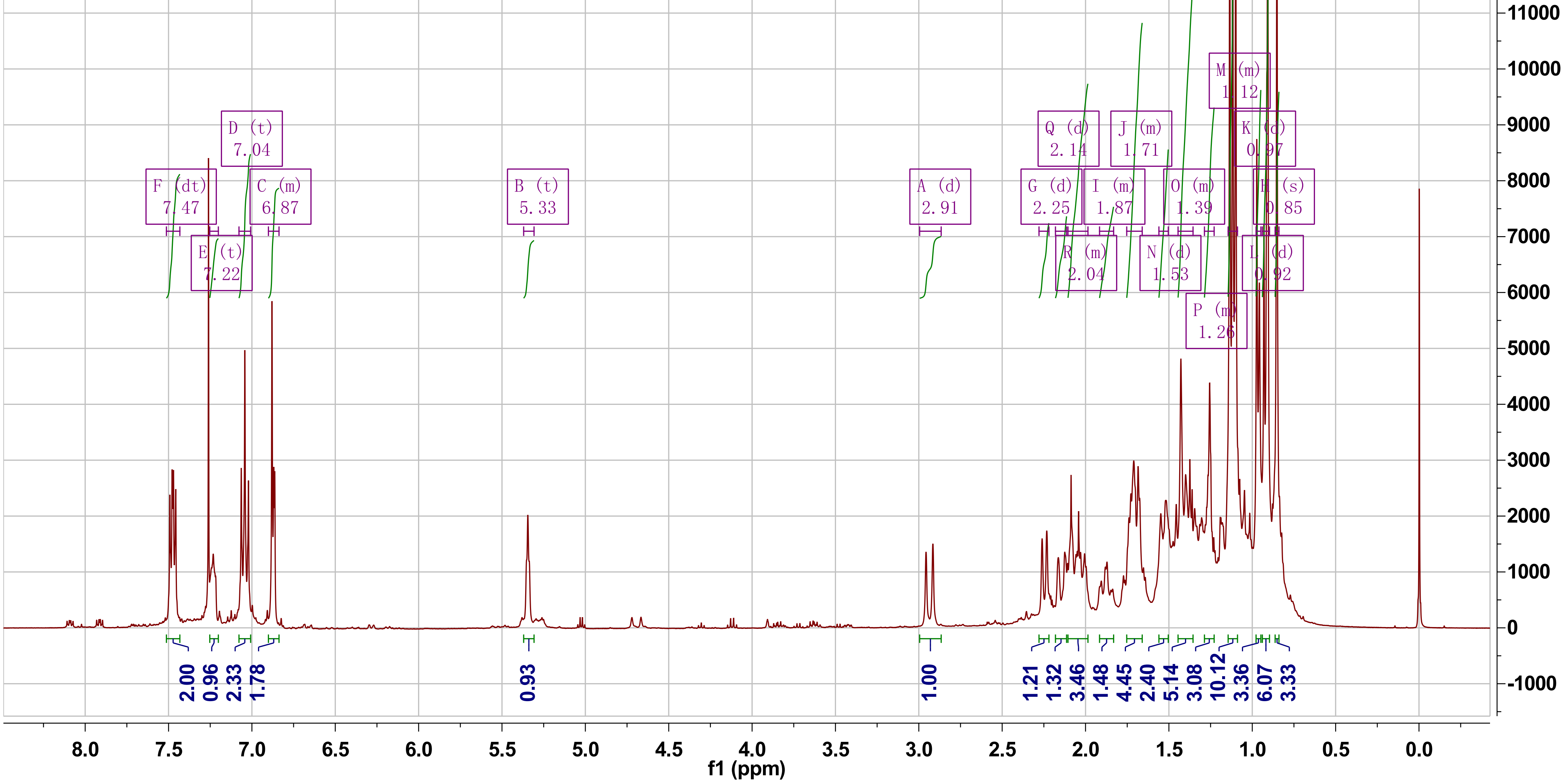

^{13}C NMR (100 MHz, $CDCl_3$) δ 207.28, 183.62, 163.1, 139.44, 138.33, 137.22, 133.10, 132.93, 129.00, 128.92, 125.72, 123.31, 116.07, 115.85, 53.30, 52.95, 48.28, 45.33, 45.22, 42.69, 42.42, 39.55, 39.27, 38.98, 36.83, 36.18, 32.35, 30.79, 29.72, 28.14, 24.27, 23.81, 23.58, 22.78, 21.28, 20.40, 17.17, 16.95, 15.69.

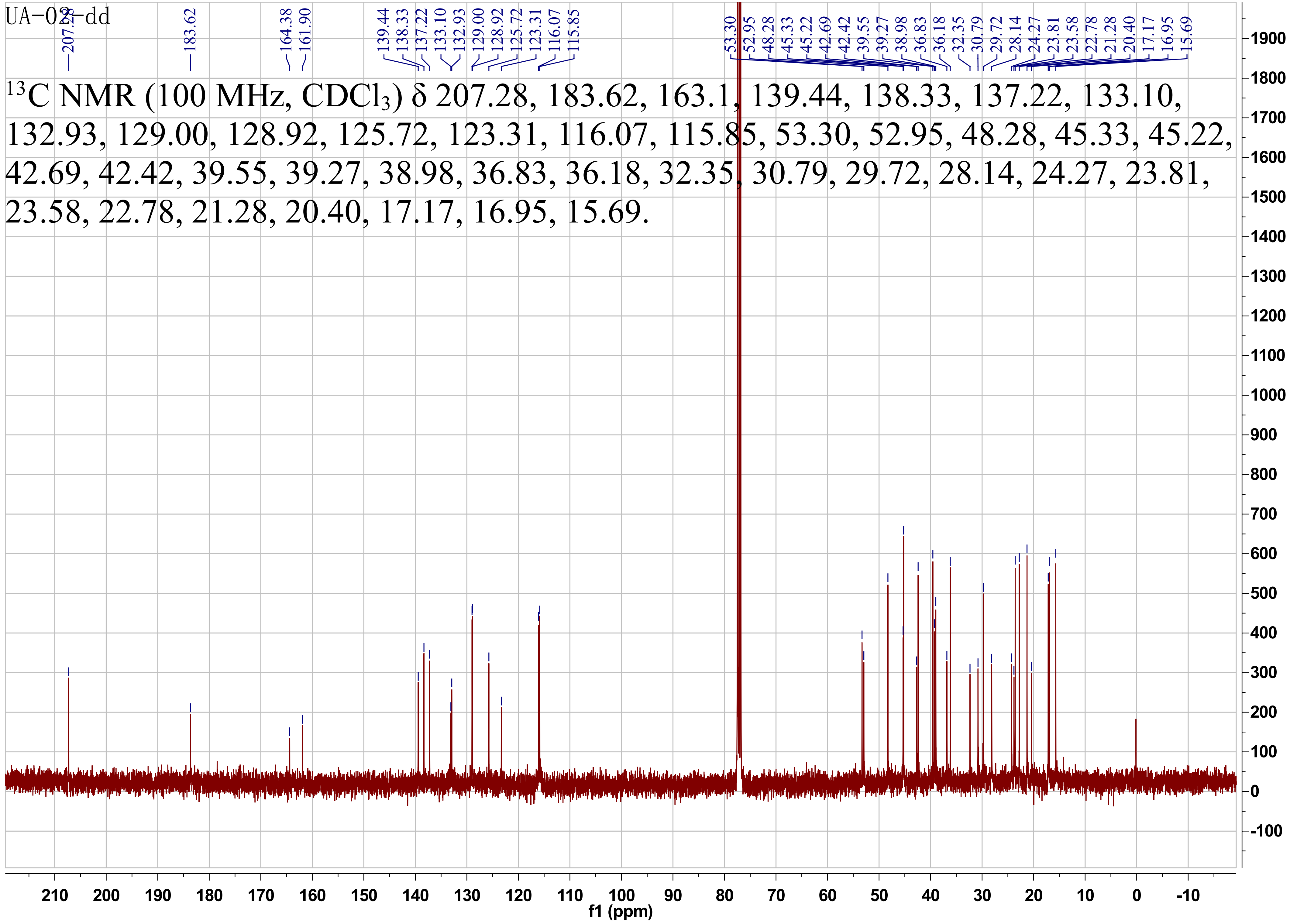

Compound code: UA-O-d

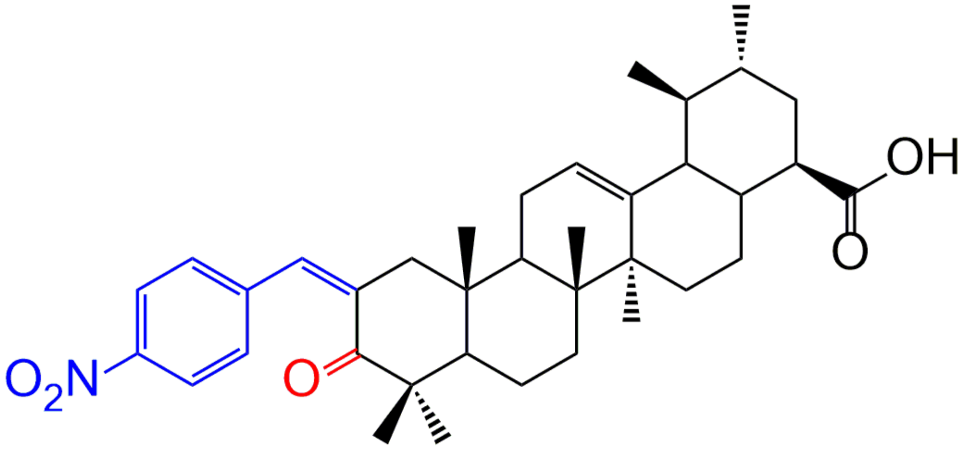

Figure 9. The structure of compound UA-O-d.

UA-02-f

^{1}H NMR (400 MHz, $CDCl_3$) δ 8.27 (d, *J* = 8.5 Hz, 2H), 7.56 (s, 1H), 7.53 (s, 2H), 5.27 (s, 1H), 2.96 (d, *J* = 16.3 Hz, 1H), 2.29 (d, *J* = 16.7 Hz, 1H), 2.21 (d, *J* = 11.3 Hz, 1H), 2.07 – 1.80 (m, 4H), 1.78 – 1.60 (m, 4H), 1.60 – 1.46 (m, 4H), 1.45 – 1.23 (m, 6H), 1.19 – 1.07 (m, 10H), 0.98 – 0.93 (m, 3H), 0.90 (d, *J* = 6.2 Hz, 3H), 0.87 (s, 3H), 0.82 (d, *J* = 6.8 Hz, 3H).

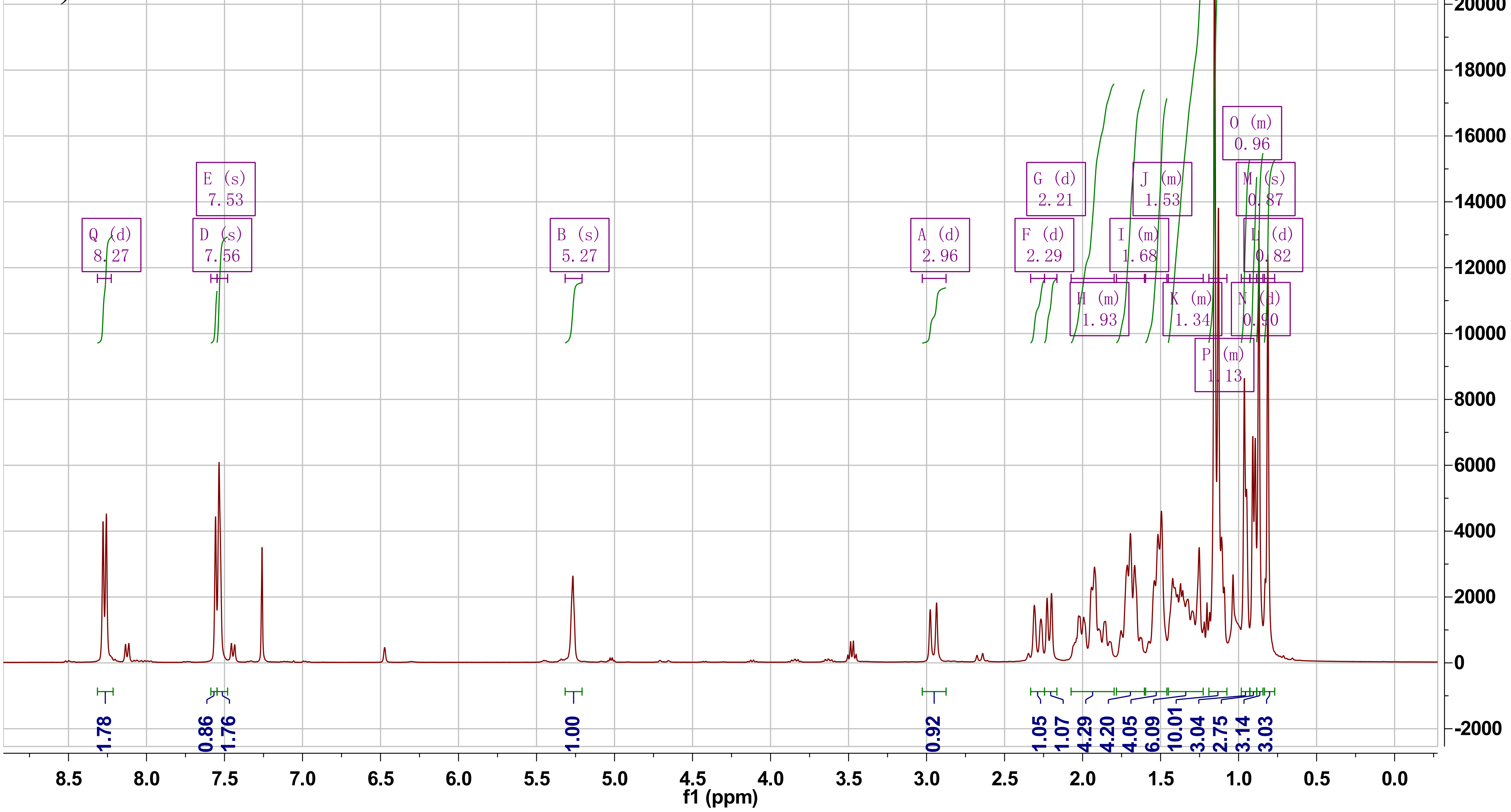

^{13}C NMR (100 MHz, $CDCl_3$) δ 207.38, 183.43, 147.35, 142.64, 138.39, 137.39, 134.83, 130.77, 125.43, 123.83, 53.37, 52.86, 48.22, 45.58, 45.39, 44.15, 42.38, 39.60, 39.29, 38.97, 36.83, 36.61, 32.23, 30.76, 29.66, 28.14, 24.23, 23.74, 23.61, 22.87, 21.27, 20.39, 17.21, 16.87, 15.62.

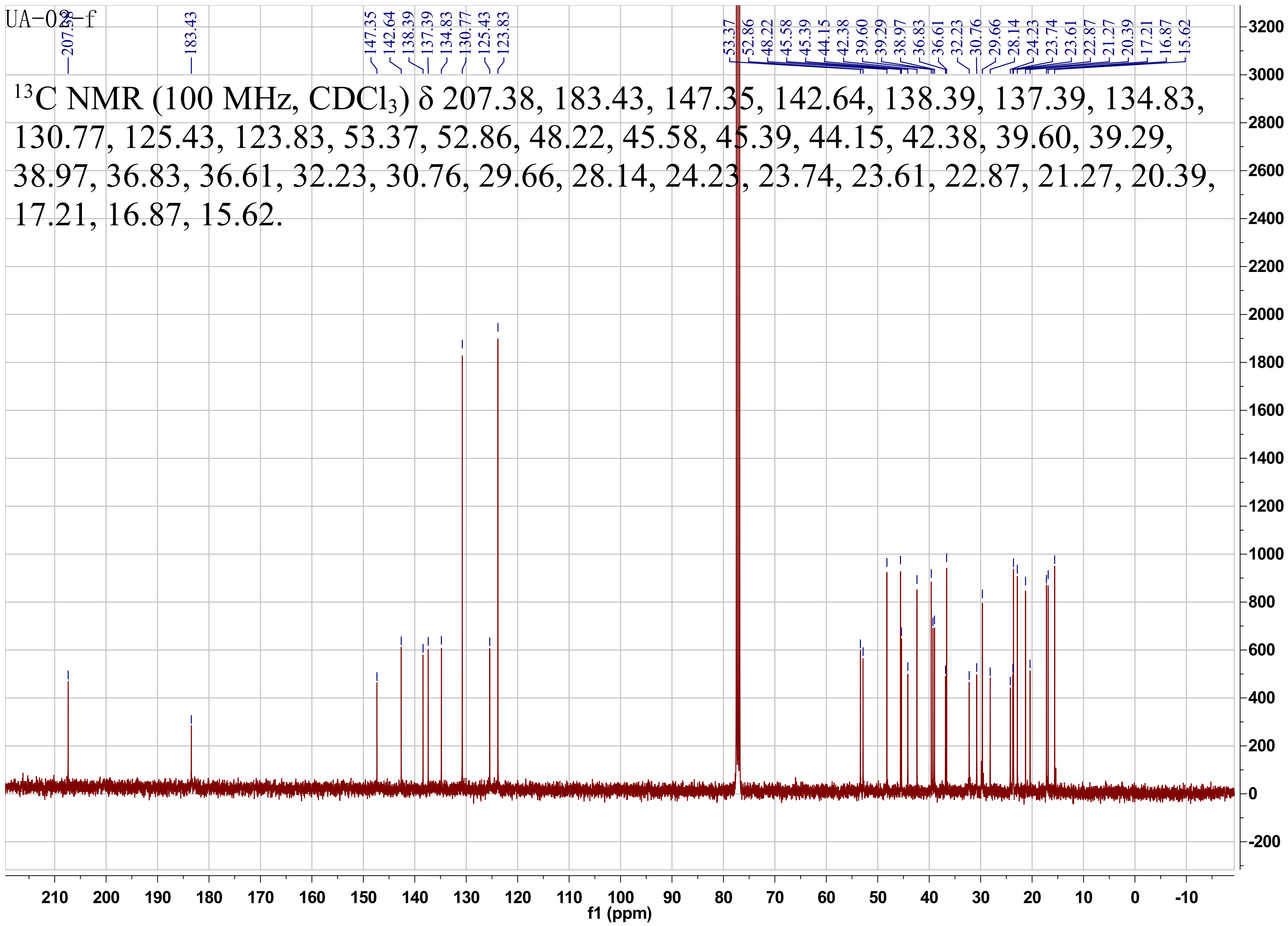

Compound code: UA-O-e

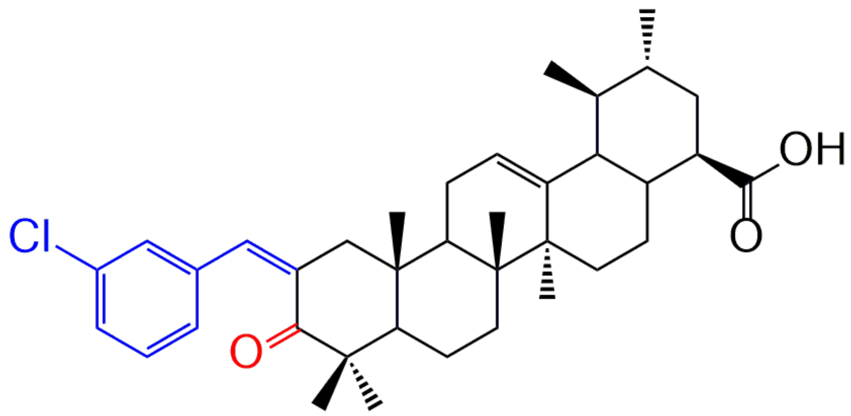

Figure 10. The structure of compound UA-O-e.

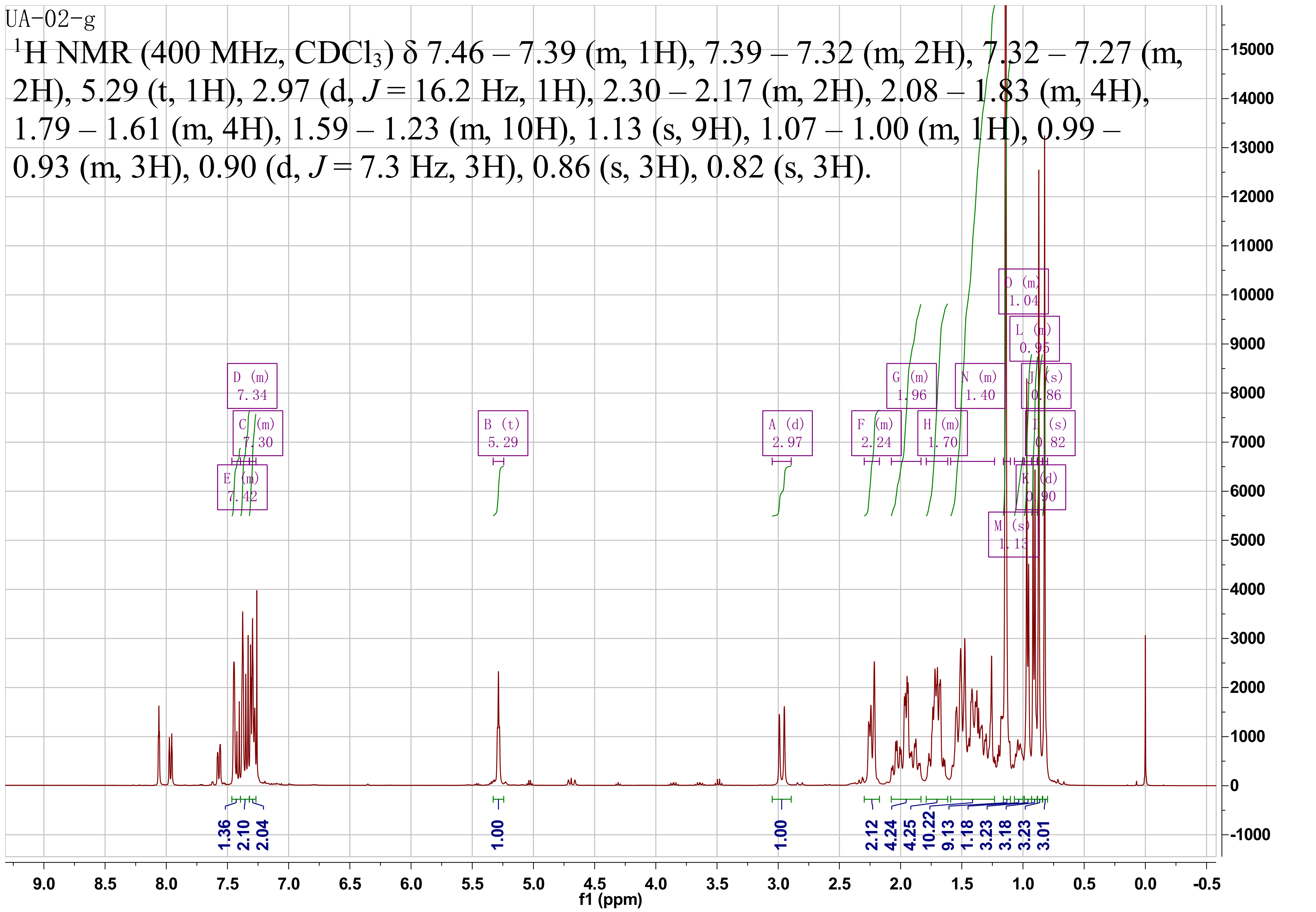
UA-02-g
^{1}H NMR (400 MHz, $CDCl_3$) δ 7.46 – 7.39 (m, 1H), 7.39 – 7.32 (m, 2H), 7.32 – 7.27 (m, 2H), 5.29 (t, 1H), 2.97 (d, J = 16.2 Hz, 1H), 2.30 – 2.17 (m, 2H), 2.08 – 1.83 (m, 4H), 1.79 – 1.61 (m, 4H), 1.59 – 1.23 (m, 10H), 1.13 (s, 9H), 1.07 – 1.00 (m, 1H), 0.99 – 0.93 (m, 3H), 0.90 (d, J = 7.3 Hz, 3H), 0.86 (s, 3H), 0.82 (s, 3H).
f1 (ppm)
1.36 2.10 2.04 1.00 1.00 2.12 4.24 4.25 10.22 9.13 1.18 3.23 3.18 3.23 3.01

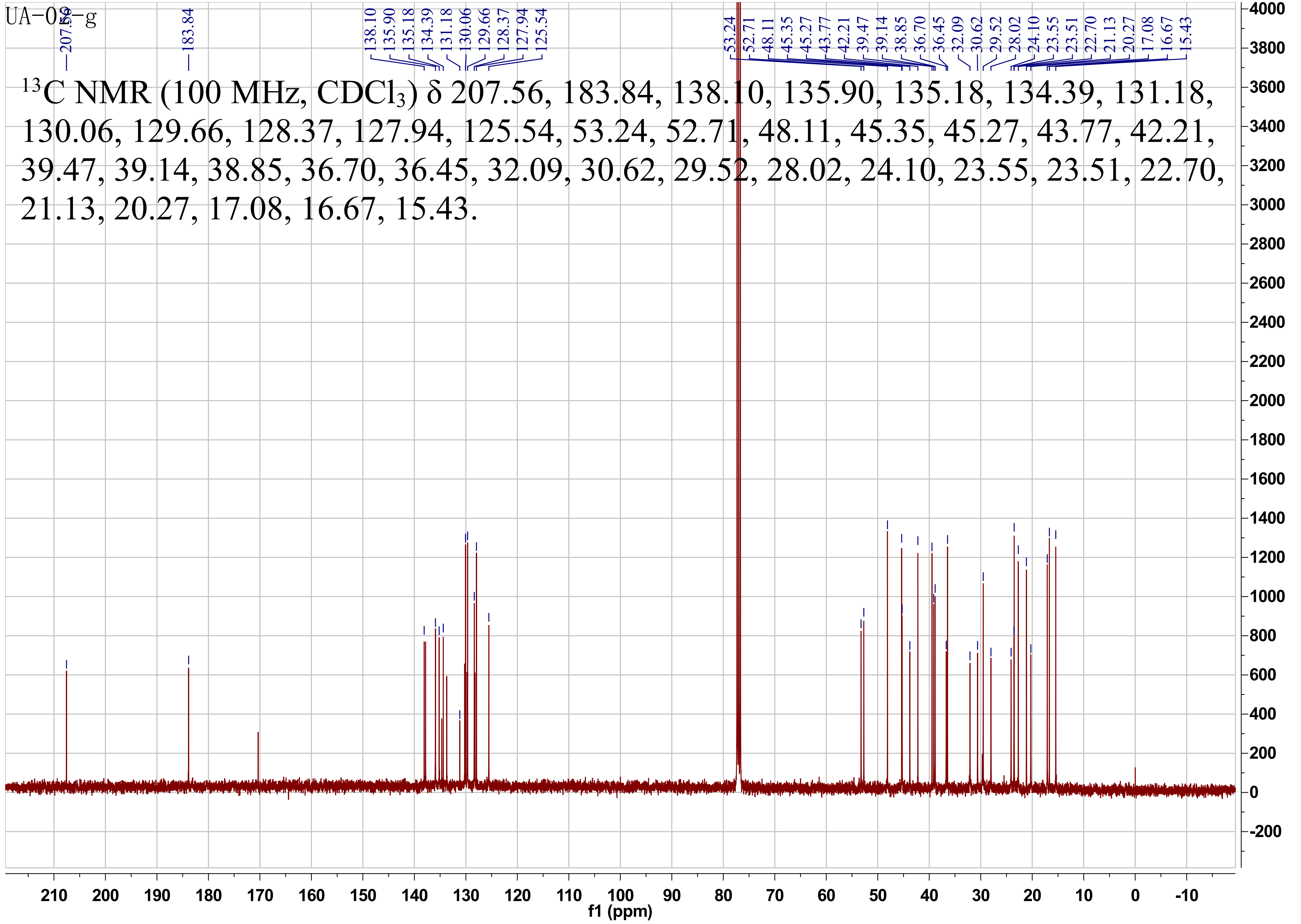

^{13}C NMR (100 MHz, $CDCl_3$) δ 207.56, 183.84, 138.10, 135.90, 135.18, 134.39, 131.18, 130.06, 129.66, 128.37, 127.94, 125.54, 53.24, 52.71, 48.11, 45.35, 45.27, 43.77, 42.21, 39.47, 39.14, 38.85, 36.70, 36.45, 32.09, 30.62, 29.52, 28.02, 24.10, 23.55, 23.51, 22.70, 21.13, 20.27, 17.08, 16.67, 15.43.

Compound code: UA-O-f

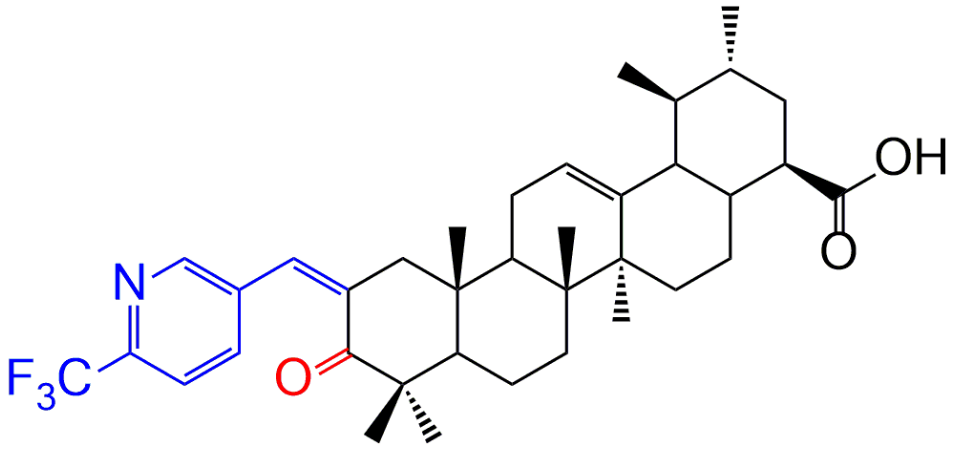

Figure 11. The structure of compound UA-O-f.

UA-02-h

^{1}H NMR (400 MHz, $CDCl_3$) δ 8.83 (s, 1H), 7.90 (d, *J* = 8.1 Hz, 1H), 7.76 (d, *J* = 8.1 Hz, 1H), 7.52 (s, 1H), 5.30 (t, *J* = 3.2 Hz, 1H), 3.00 (d, *J* = 15.4 Hz, 1H), 2.34 (d, *J* = 17.3 Hz, 1H), 2.24 (d, 1H), 2.10 – 1.93 (m, 3H), 1.88 (td, *J* = 13.3, 3.6 Hz, 1H), 1.80 – 1.64 (m, 4H), 1.59 – 1.48 (m, 4H), 1.48 – 1.26 (m, 6H), 1.19 (d, *J* = 6.2 Hz, 6H), 1.15 (s, 3H), 1.09 – 1.03 (m, 1H), 0.98 (d, *J* = 6.0 Hz, 3H), 0.93 (d, *J* = 6.3 Hz, 3H), 0.90 (s, 3H), 0.84 (s, 3H).

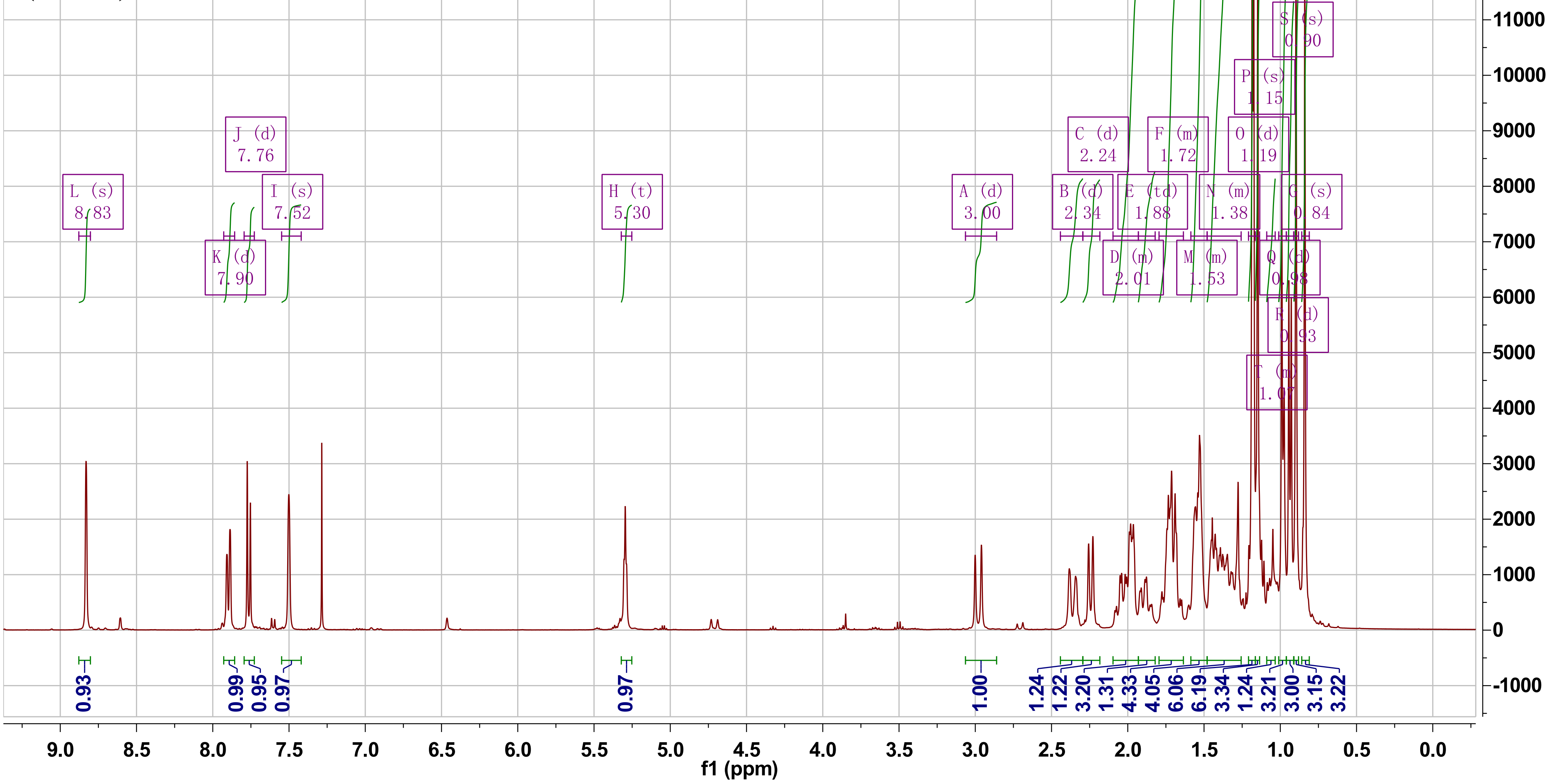

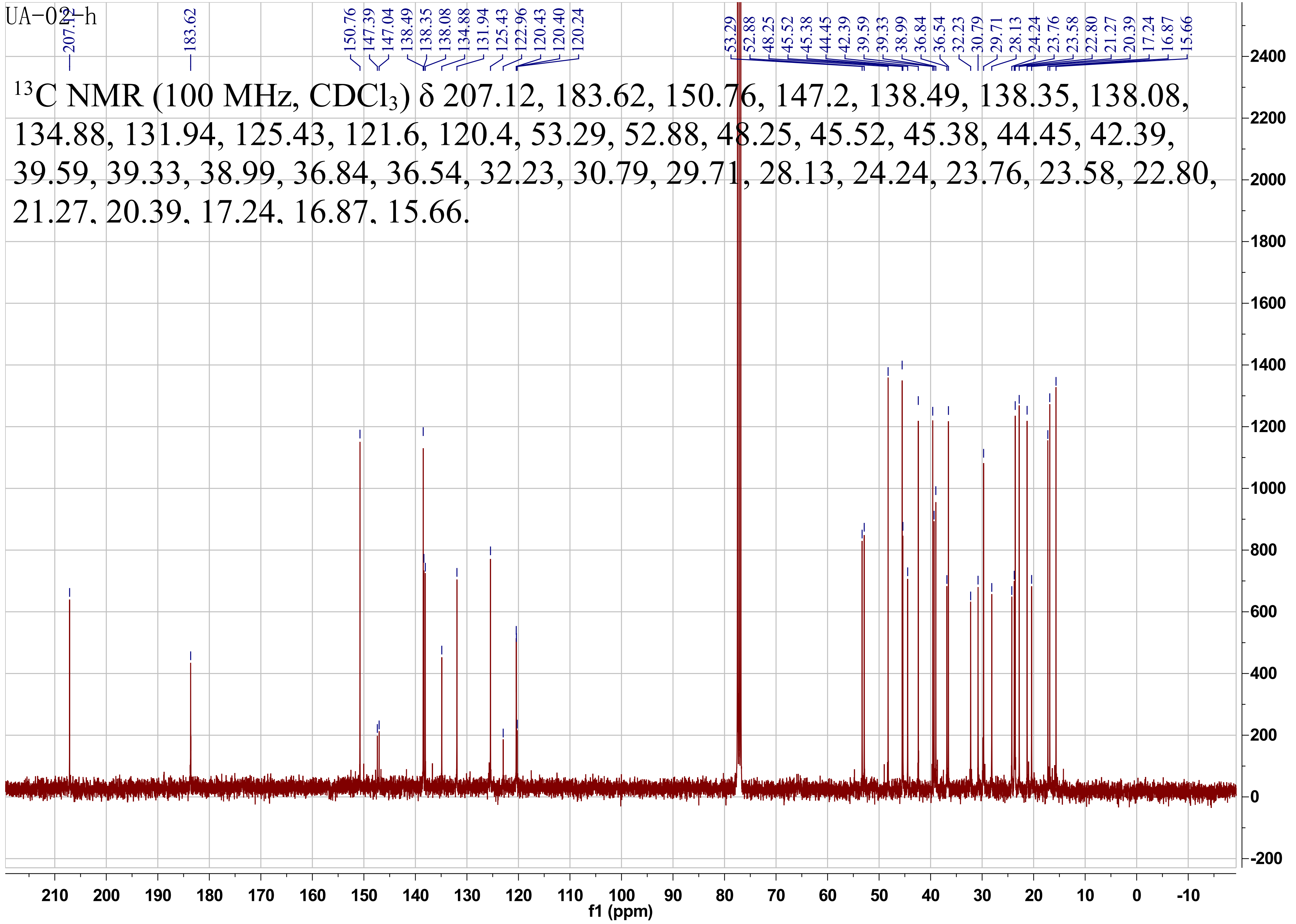

^{13}C NMR (100 MHz, $CDCl_3$) δ 207.12, 183.62, 150.76, 147.2, 138.49, 138.35, 138.08, 134.88, 131.94, 125.43, 121.6, 120.4, 53.29, 52.88, 48.25, 45.52, 45.38, 44.45, 42.39, 39.59, 39.33, 38.99, 36.84, 36.54, 32.23, 30.79, 29.71, 28.13, 24.24, 23.76, 23.58, 22.80, 21.27, 20.39, 17.24, 16.87, 15.66.

Compound code: UA-O-g

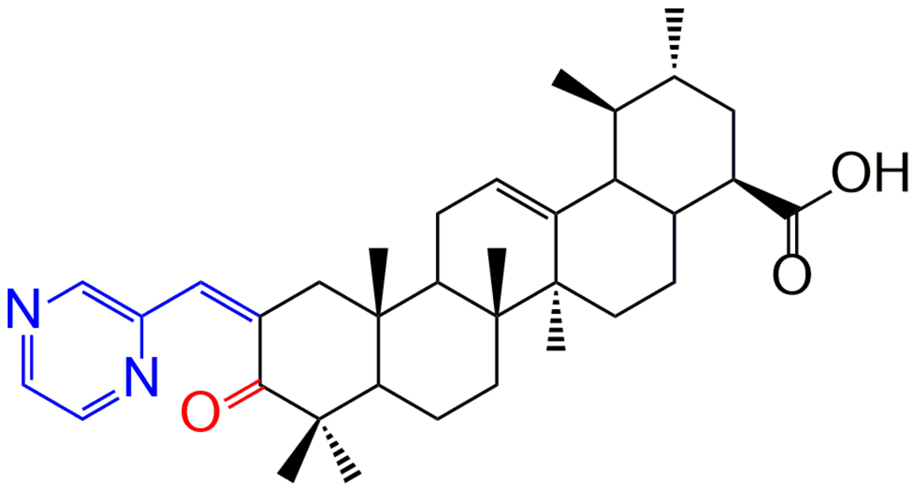

Figure 12. The structure of compound UA-O-g.

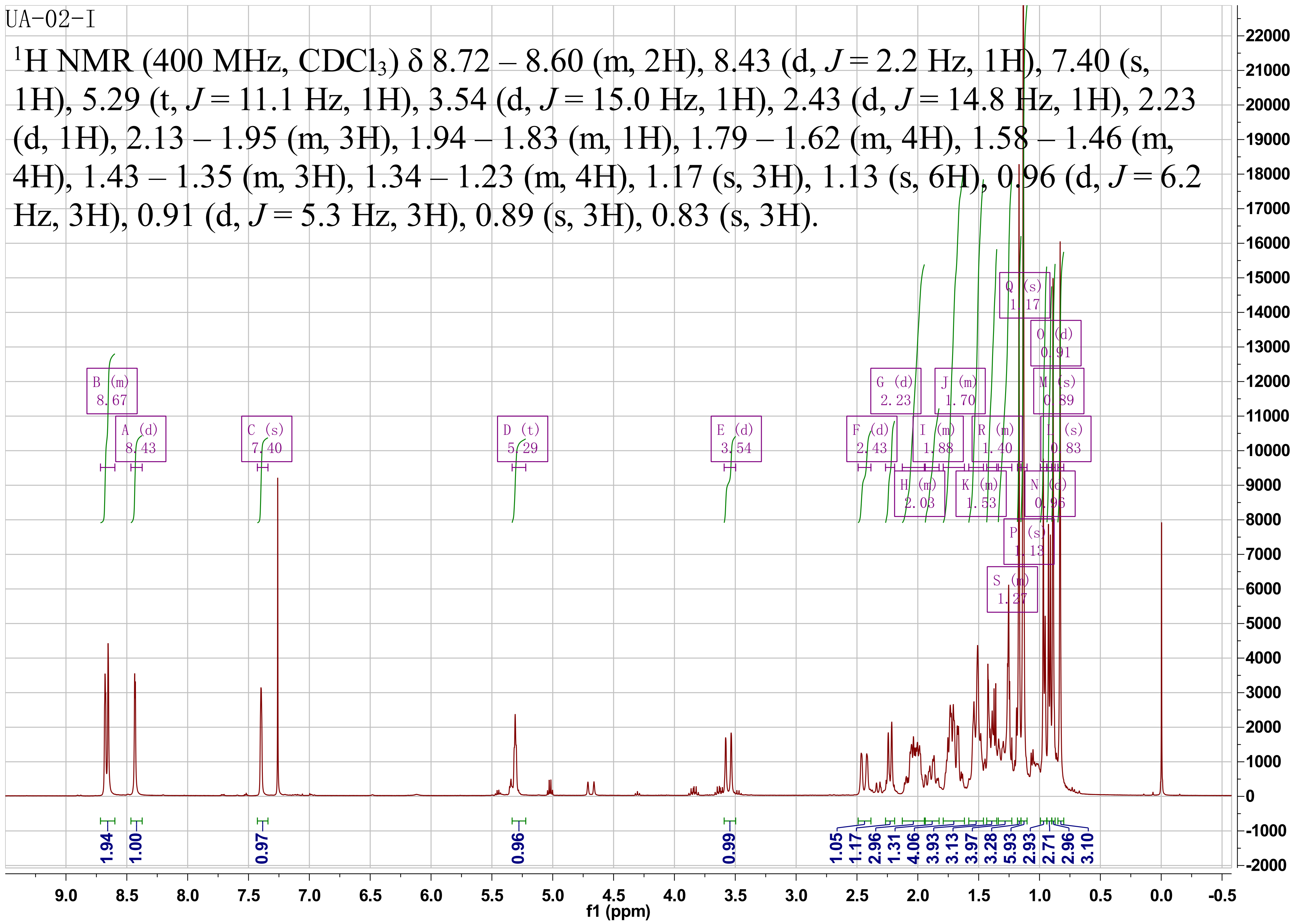
UA-02-I
1H NMR (400 MHz, CDCl3) δ 8.72 – 8.60 (m, 2H), 8.43 (d, J = 2.2 Hz, 1H), 7.40 (s, 1H), 5.29 (t, J = 11.1 Hz, 1H), 3.54 (d, J = 15.0 Hz, 1H), 2.43 (d, J = 14.8 Hz, 1H), 2.23 (d, 1H), 2.13 – 1.95 (m, 3H), 1.94 – 1.83 (m, 1H), 1.79 – 1.62 (m, 4H), 1.58 – 1.46 (m, 4H), 1.43 – 1.35 (m, 3H), 1.34 – 1.23 (m, 4H), 1.17 (s, 3H), 1.13 (s, 6H), 0.96 (d, J = 6.2 Hz, 3H), 0.91 (d, J = 5.3 Hz, 3H), 0.89 (s, 3H), 0.83 (s, 3H).
f1 (ppm)

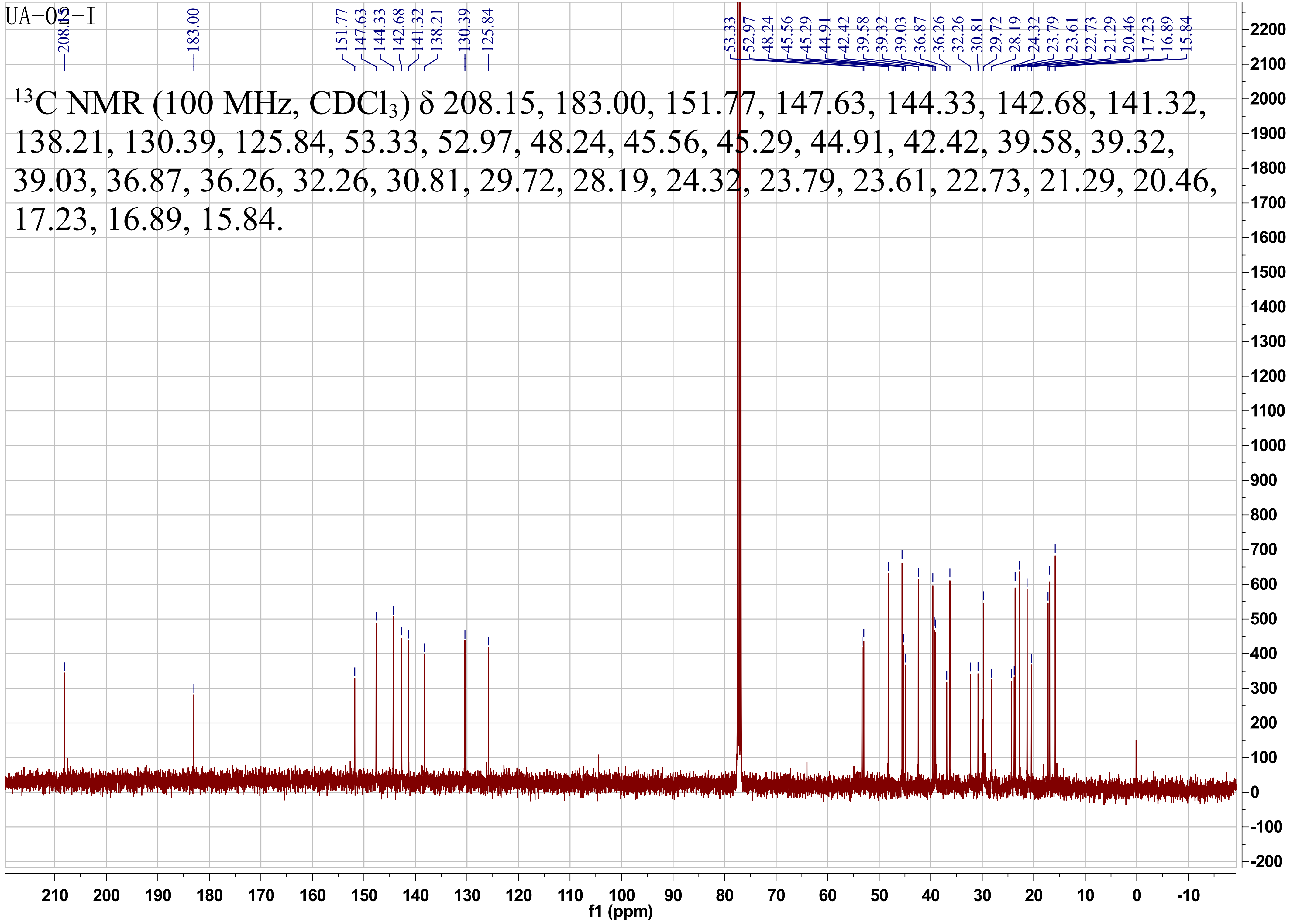
UA-02-I
^{13}C NMR (100 MHz, $CDCl_3$) δ 208.15, 183.00, 151.77, 147.63, 144.33, 142.68, 141.32, 138.21, 130.39, 125.84, 53.33, 52.97, 48.24, 45.56, 45.29, 44.91, 42.42, 39.58, 39.32, 39.03, 36.87, 36.26, 32.26, 30.81, 29.72, 28.19, 24.32, 23.79, 23.61, 22.73, 21.29, 20.46, 17.23, 16.89, 15.84.
f1 (ppm)

Compound code: UA-O-h

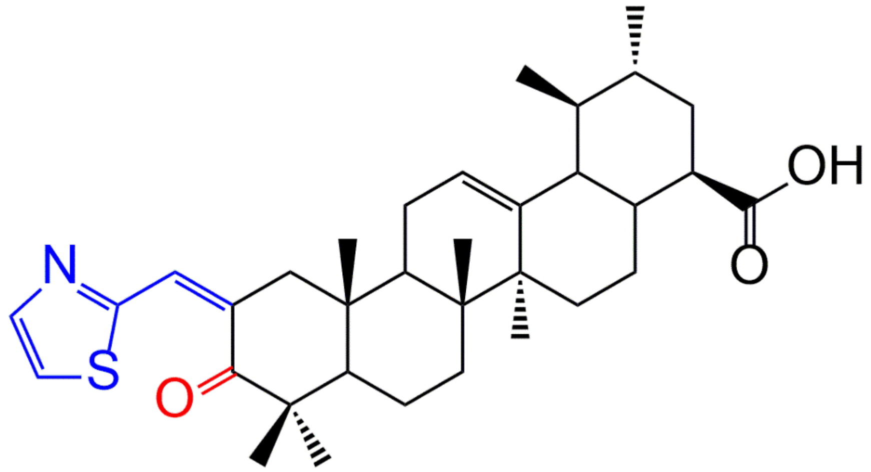

Figure 13. The structure of compound UA-O-h.

UA-02-j

^{1}H NMR (400 MHz, $CDCl_3$) δ 8.01 (d, *J* = 3.2 Hz, 1H), 7.64 (s, 1H), 7.50 (d, *J* = 5.3 Hz, 1H), 5.33 (s, 1H), 3.37 (d, *J* = 17.8 Hz, 1H), 2.34 (d, *J* = 17.2 Hz, 1H), 2.24 (d, *J* = 11.3 Hz, 1H), 2.20 – 2.10 (m, 1H), 2.09 – 1.96 (m, 2H), 1.88 (td, *J* = 13.6, 3.9 Hz, 1H), 1.81 – 1.65 (m, 4H), 1.59 – 1.48 (m, 4H), 1.44 – 1.24 (m, 7H), 1.17 (s, 3H), 1.12 (d, *J* = 7.2 Hz, 6H), 0.96 (d, *J* = 6.1 Hz, 3H), 0.92 (d, *J* = 3.5 Hz, 6H), 0.84 (s, 3H).

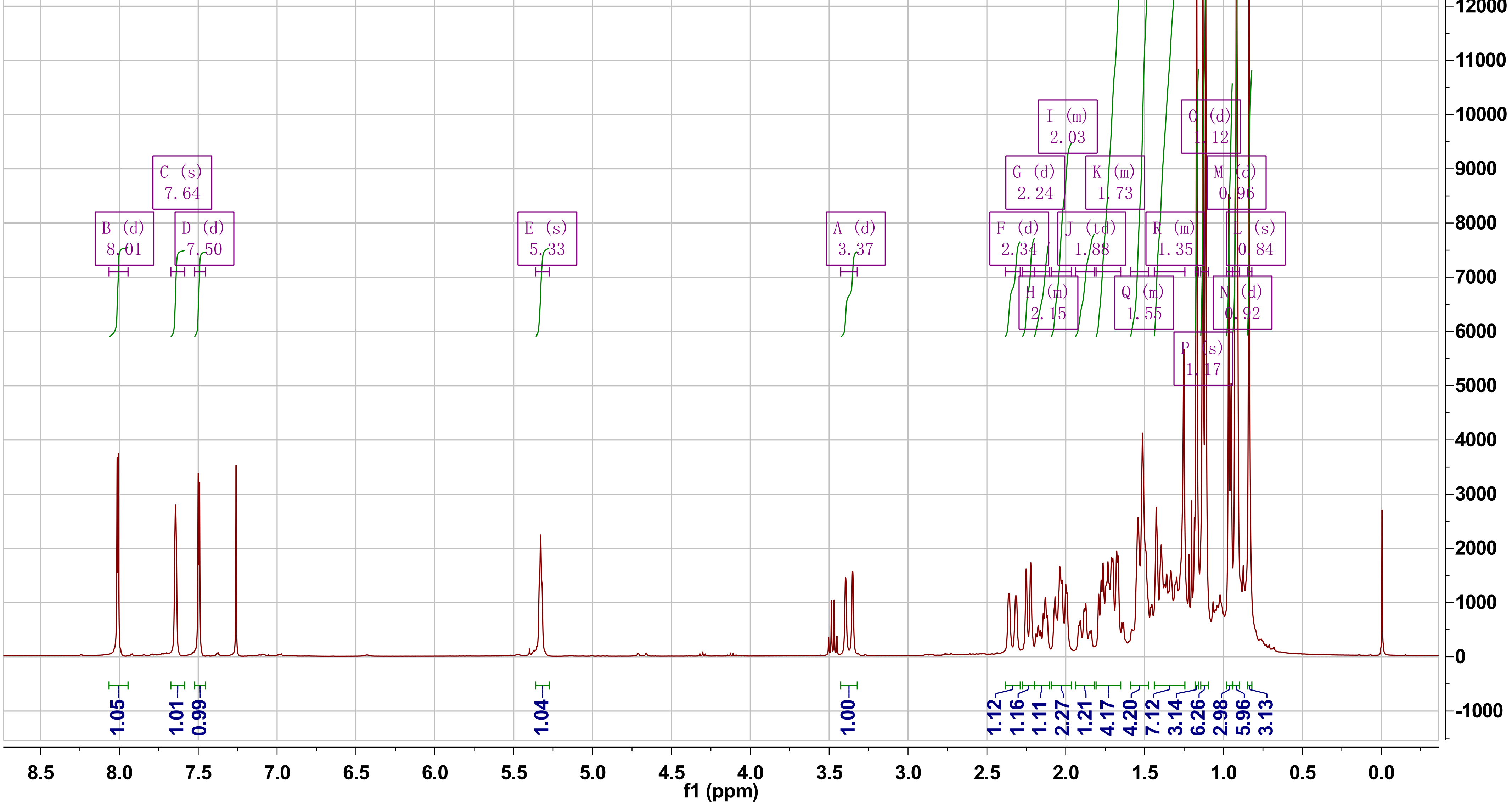

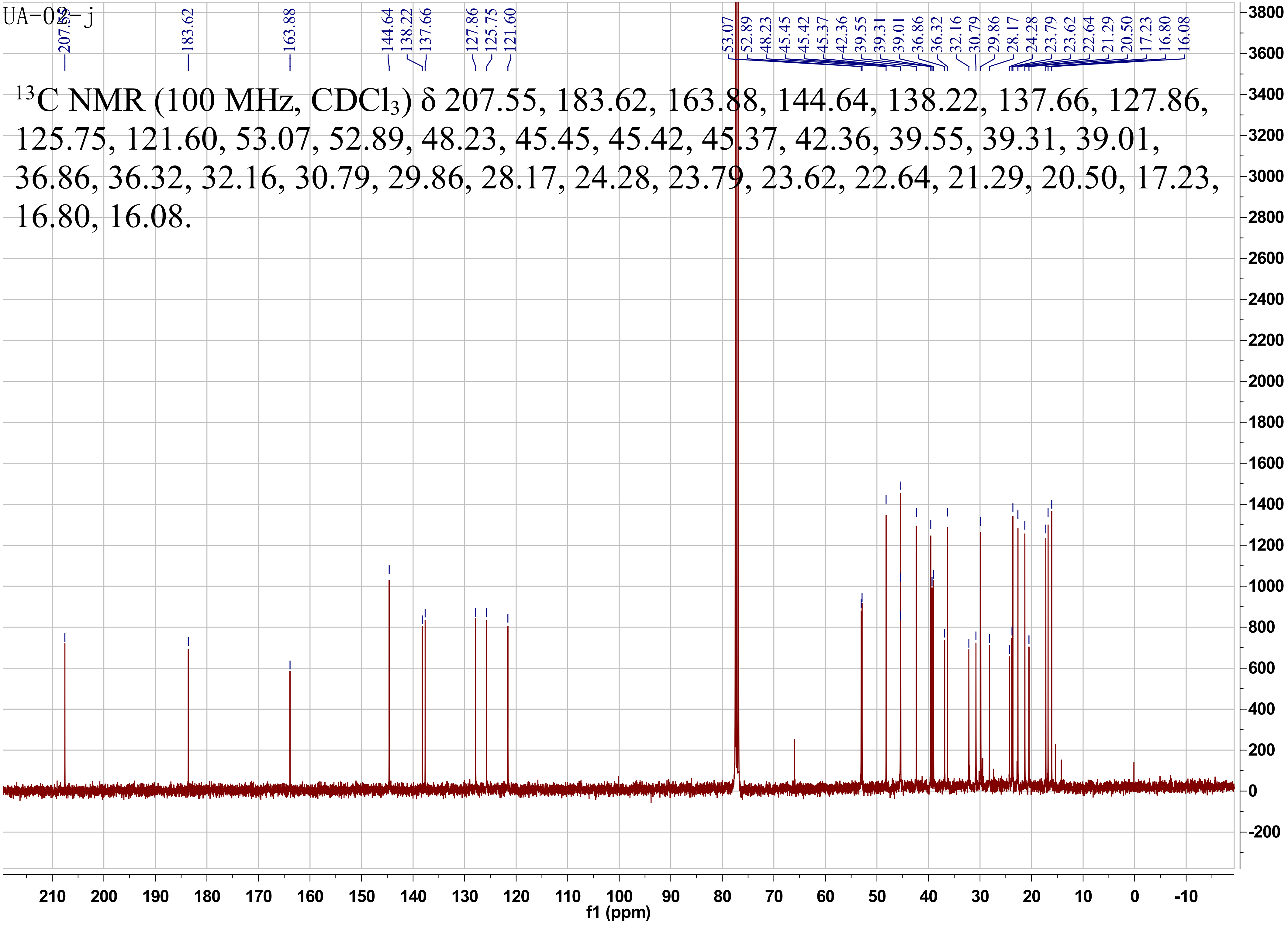

^{13}C NMR (100 MHz, $CDCl_3$) δ 207.55, 183.62, 163.88, 144.64, 138.22, 137.66, 127.86, 125.75, 121.60, 53.07, 52.89, 48.23, 45.45, 45.42, 45.37, 42.36, 39.55, 39.31, 39.01, 36.86, 36.32, 32.16, 30.79, 29.86, 28.17, 24.28, 23.79, 23.62, 22.64, 21.29, 20.50, 17.23, 16.80, 16.08.

Compound code: UA-O-i

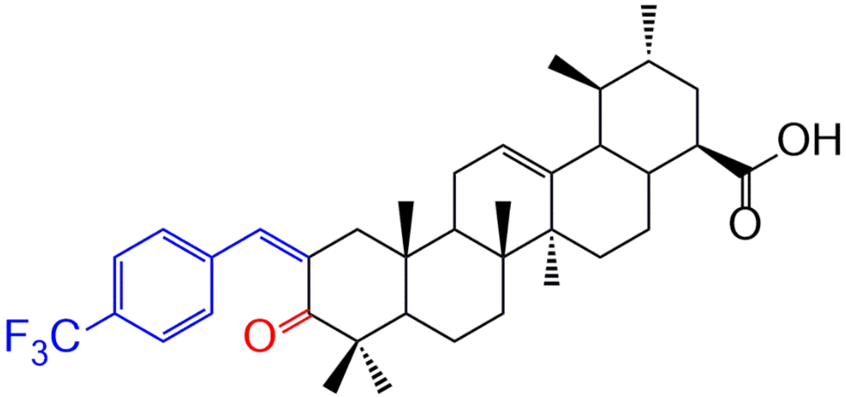

Figure 14. The structure of compound UA-O-i.

UA-02-K

^{1}H NMR (400 MHz, $CDCl_3$) δ 7.67 (d, *J* = 8.2 Hz, 2H), 7.51 (d, *J* = 8.5 Hz, 3H), 5.27 (t, *J* = 3.2 Hz, 1H), 2.98 (d, *J* = 16.3 Hz, 1H), 2.34 – 2.14 (m, 2H), 2.09 – 1.97 (m, 1H), 1.94 (dd, *J* = 8.6, 3.2 Hz, 2H), 1.86 (td, *J* = 13.7, 4.0 Hz, 1H), 1.78 – 1.61 (m, 4H), 1.59 – 1.46 (m, 4H), 1.46 – 1.18 (m, 6H), 1.14 (d, *J* = 7.9 Hz, 9H), 1.08 – 0.99 (m, 1H), 0.96 (d, *J* = 6.1 Hz, 3H), 0.90 (d, *J* = 5.7 Hz, 3H), 0.87 (s, 3H), 0.81 (s, 3H).

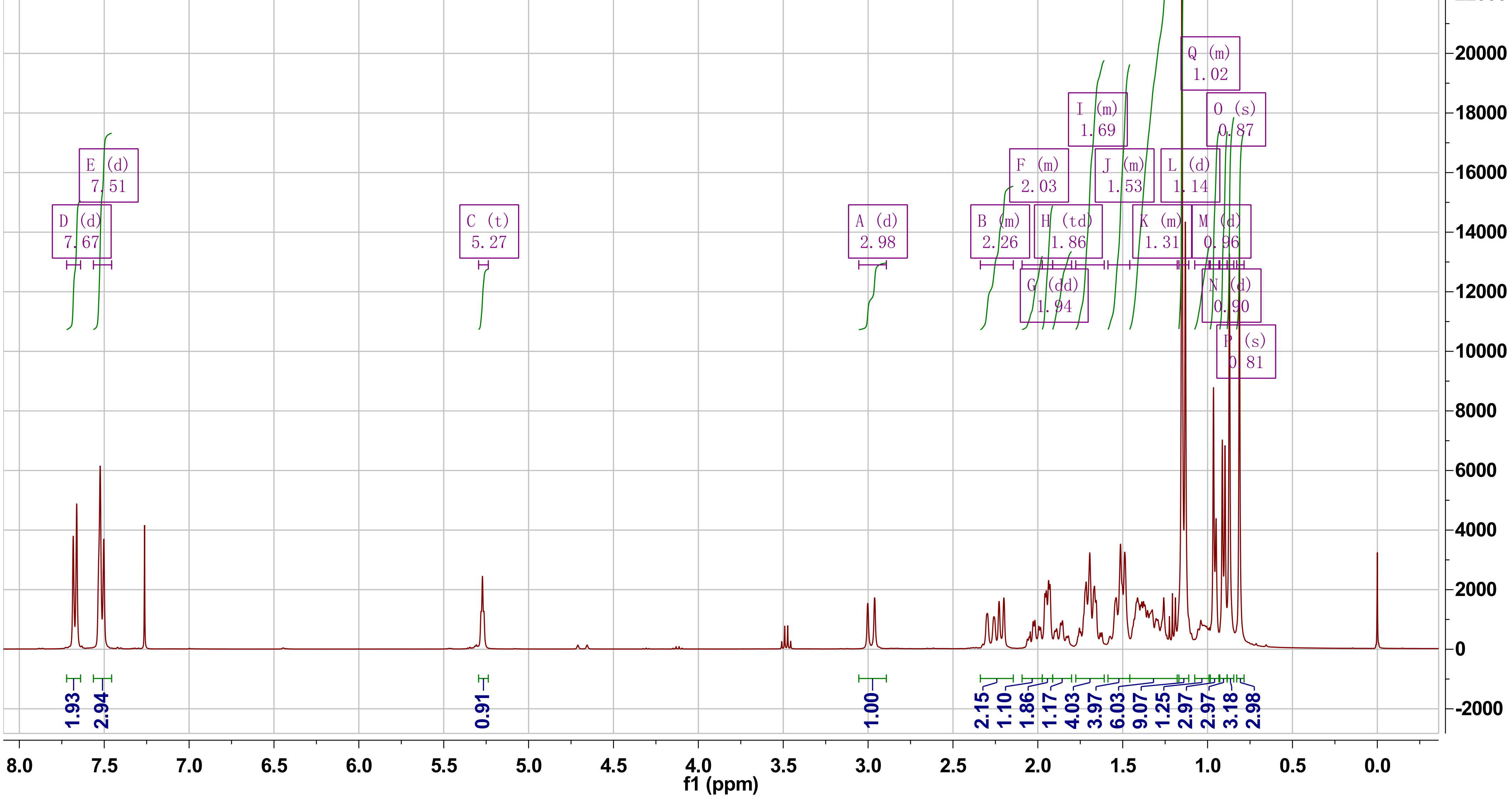

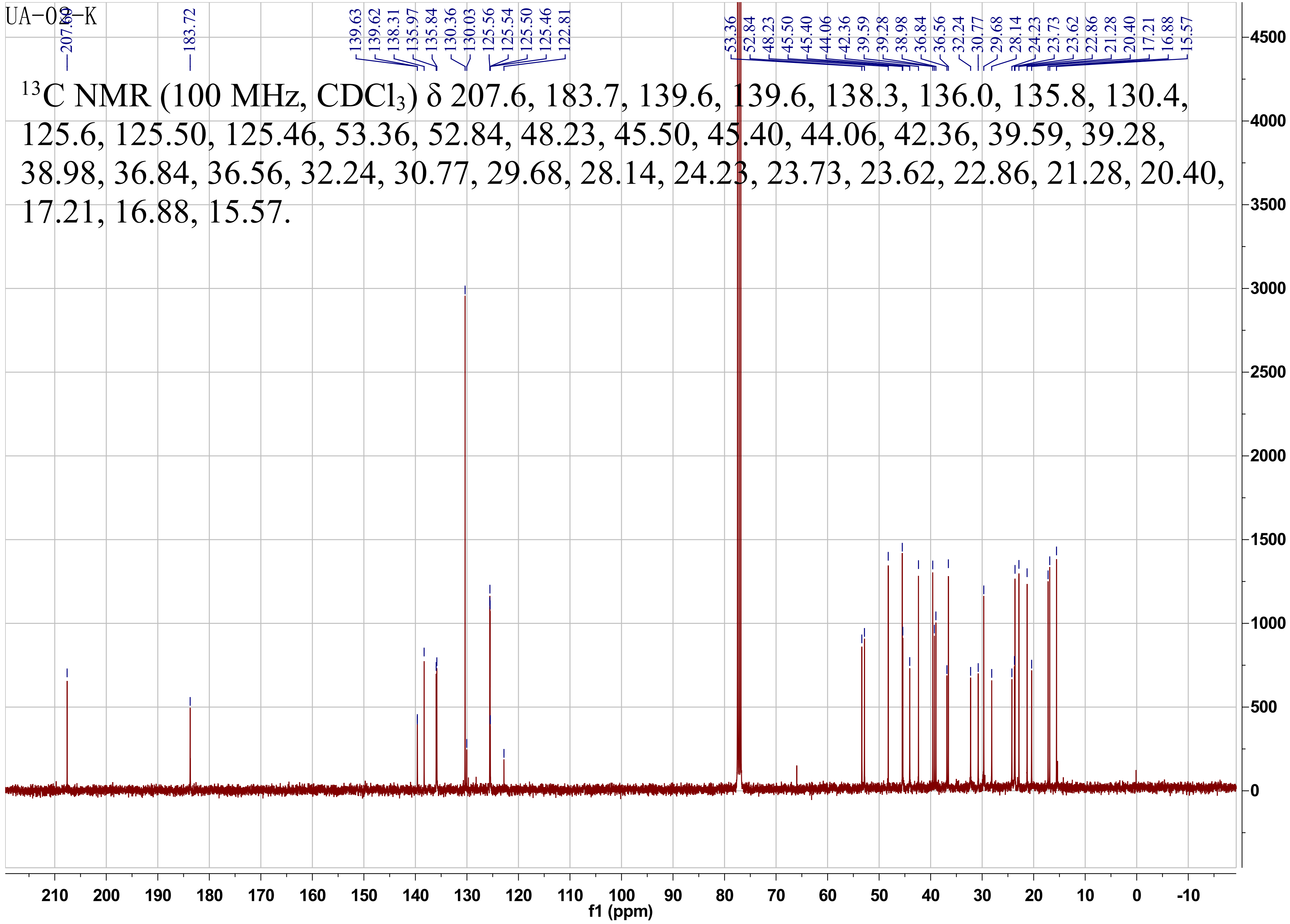

^{13}C NMR (100 MHz, $CDCl_3$) δ 207.6, 183.7, 139.6, 139.6, 138.3, 136.0, 135.8, 130.4, 125.6, 125.50, 125.46, 53.36, 52.84, 48.23, 45.50, 45.40, 44.06, 42.36, 39.59, 39.28, 38.98, 36.84, 36.56, 32.24, 30.77, 29.68, 28.14, 24.23, 23.73, 23.62, 22.86, 21.28, 20.40, 17.21, 16.88, 15.57.

Compound code: UA-O-j

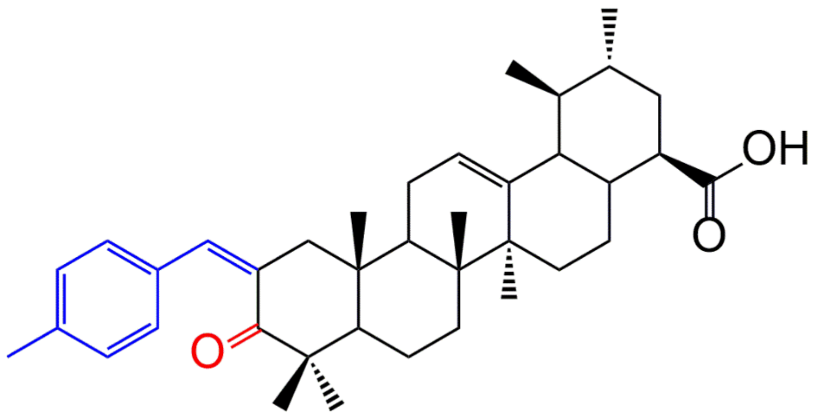

Figure 15. The structure of compound UA-O-j.

UA-02-L

^{1}H NMR (400 MHz, $CDCl_3$) δ 7.53 (s, 1H), 7.35 (d, J = 8.0 Hz, 2H), 7.23 (d, J = 7.9 Hz, 2H), 5.28 (s, 1H), 3.03 (d, J = 16.2 Hz, 1H), 2.38 (s, 3H), 2.31 – 2.24 (m, 1H), 2.22 (d, J = 11.4 Hz, 1H), 2.09 – 1.93 (m, 3H), 1.86 (td, J = 13.4, 3.8 Hz, 1H), 1.78 – 1.61 (m, 4H), 1.59 – 1.45 (m, 4H), 1.45 – 1.33 (m, 4H), 1.26 (s, 3H), 1.14 (d, J = 3.3 Hz, 9H), 0.96 (d, J = 6.0 Hz, 3H), 0.91 (d, J = 6.4 Hz, 3H), 0.87 (s, 3H), 0.81 (s, 3H).

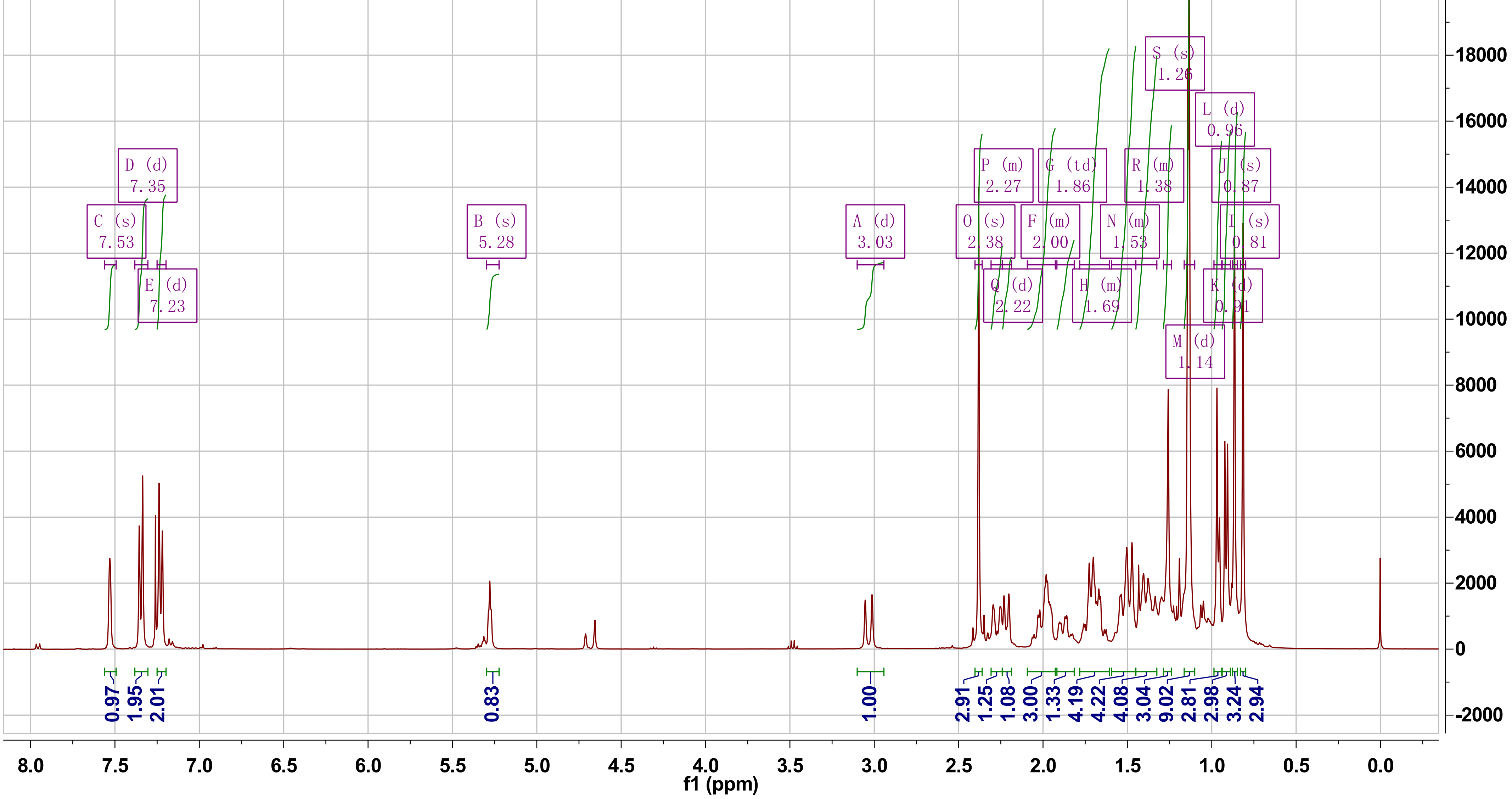

UA-02-L

^{13}C NMR (100 MHz, $CDCl_3$) δ 207.88, 183.75, 138.79, 138.22, 137.81, 133.32, 133.07, 130.55, 129.35, 125.77, 53.25, 52.87, 48.24, 45.47, 45.29, 44.29, 42.35, 39.59, 39.29, 39.00, 36.85, 36.44, 32.27, 30.78, 29.84, 28.16, 24.27, 23.75, 23.63, 22.83, 21.52, 21.29, 20.45, 17.22, 16.87, 15.56.

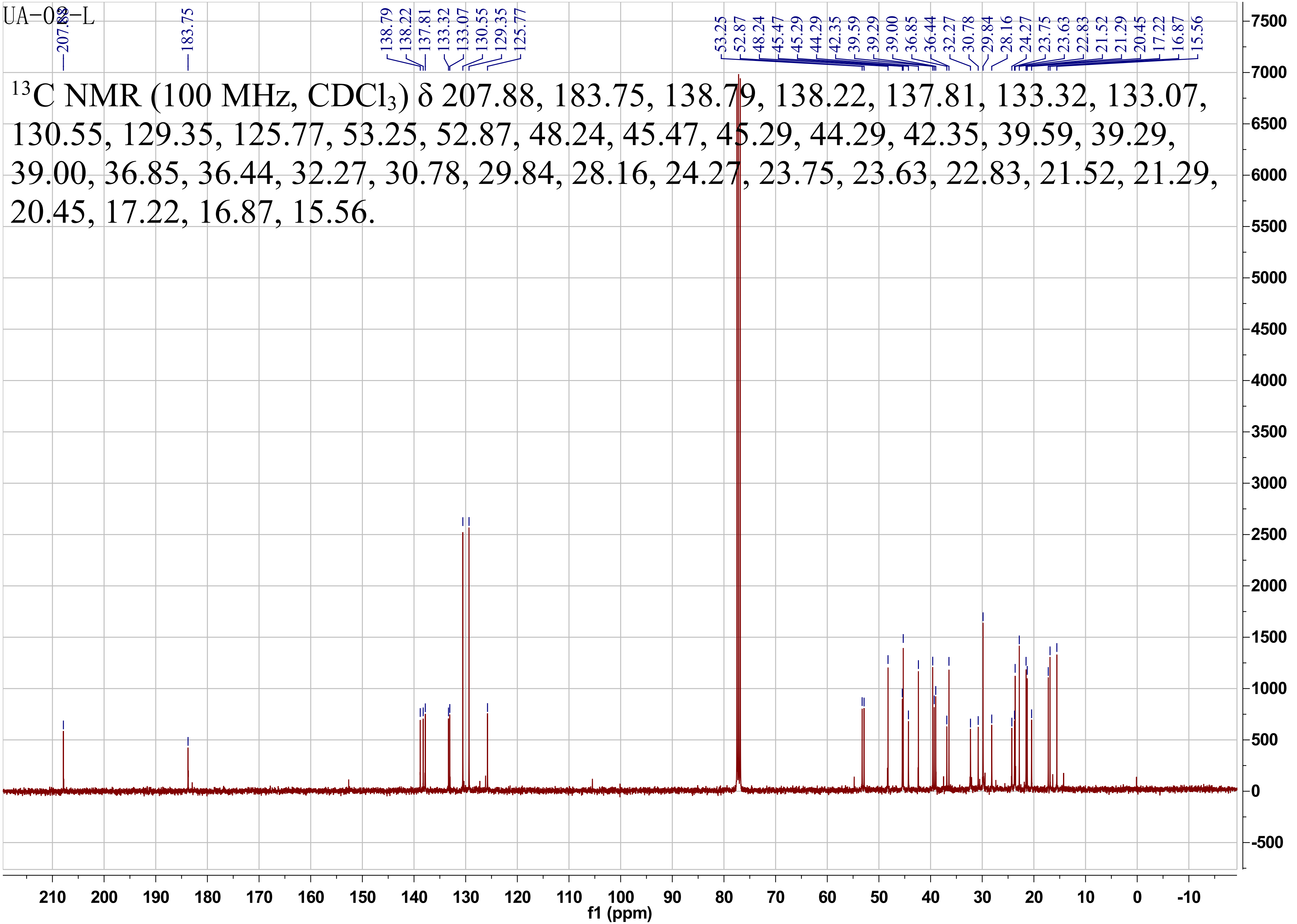

Supplement: Supplementary Information [file srep45578-s1.pdf]
